# LINE retrotransposons characterize mammalian tissue-specific and evolutionarily dynamic regulatory regions

**DOI:** 10.1186/s13059-021-02260-y

**Published:** 2021-02-18

**Authors:** Maša Roller, Ericca Stamper, Diego Villar, Osagie Izuogu, Fergal Martin, Aisling M. Redmond, Raghavendra Ramachanderan, Louise Harewood, Duncan T. Odom, Paul Flicek

**Affiliations:** 1 European Molecular Biology Laboratory, European Bioinformatics Institute, Wellcome Genome Campus, Hinxton, Cambridge, CB10 1SD UK; 2 Cancer Research UK Cambridge Institute, University of Cambridge, Robinson Way, Cambridge, CB2 0RE UK; 3 Present address: Harriet L. Wilkes Honors College, Florida Atlantic University, Jupiter, FL 33458 USA; 4 Present address: Blizard Institute, Barts and The London School of Medicine and Dentistry, Queen Mary University of London, London, E1 2AT UK; 5 Present address: MRC Cancer Unit, Hutchison-MRC Research Centre, University of Cambridge, Cambridge, CB2 0XZ UK; 6 Present address: Precision Medicine Centre of Excellence, Queen’s University Belfast, Belfast, BT9 7AE UK; 7 German Cancer Research Center (DKFZ), Division of Regulatory Genomics and Cancer Evolution, Im Neuenheimer Feld 280, 69120 Heidelberg, Germany; 8 Wellcome Sanger Institute, Wellcome Genome Campus, Hinxton, Cambridge, CB10 1SD UK

**Keywords:** Regulatory evolution, Gene regulation, Promoters, Enhancers, Transposable elements, Long Interspersed Nuclear Elements (LINEs), LINE L1, LINE L2, Mammals

## Abstract

**Background:**

To investigate the mechanisms driving regulatory evolution across tissues, we experimentally mapped promoters, enhancers, and gene expression in the liver, brain, muscle, and testis from ten diverse mammals.

**Results:**

The regulatory landscape around genes included both tissue-shared and tissue-specific regulatory regions, where tissue-specific promoters and enhancers evolved most rapidly. Genomic regions switching between promoters and enhancers were more common across species, and less common across tissues within a single species. Long Interspersed Nuclear Elements (LINEs) played recurrent evolutionary roles: LINE L1s were associated with tissue-specific regulatory regions, whereas more ancient LINE L2s were associated with tissue-shared regulatory regions and with those switching between promoter and enhancer signatures across species.

**Conclusions:**

Our analyses of the tissue-specificity and evolutionary stability among promoters and enhancers reveal how specific LINE families have helped shape the dynamic mammalian regulome.

**Supplementary Information:**

The online version contains supplementary material available at 10.1186/s13059-021-02260-y.

## Background

Mammalian tissues are composed of hundreds of cell types, each with its own tissue-specific gene expression program. These programs are controlled by proximal promoters and distal enhancer regions [1].

Promoters and enhancers are traditionally considered distinct and minimally overlapping categories, although specific genomic regions can show both promoter and enhancer activity between cell types of a species [2]. Some promoters show characteristics of enhancers, such as impacting expression of distal genes [3, 4], showing chromatin signatures of enhancers [3], or contacting another promoter [5]. Conversely, some enhancers show characteristics of promoters by driving transcription [6–8] or functioning as alternative promoters [9]. Evolutionary studies on a limited number of lineages and regulatory regions have suggested that a subset of enhancers can be repurposed to promoters across species [10].

While transcriptional divergence has been extensively characterized in mammalian tissues [11–13], the evolution of the associated regulatory regions is not well understood. Enhancer and promoter evolution has mostly been studied by comparing one mammalian tissue or cell type across several species [14–17]. This approach is unable to compare evolutionary trends across tissues. A second approach comparing the regulatory landscapes among various tissues of mouse and human [18–21] affords limited insights into the rate and lineage-specificity of regulatory evolution.

Nevertheless, these studies revealed that enhancers have a high rate of evolutionary turnover [14, 16–19]. For example, less than 5% of human embryonic stem cell enhancers are conserved in mouse [16]. Promoter regions are more evolutionarily stable [14, 18, 19], although only around half of the transcription start sites are precisely conserved between mouse and human [21, 22].

Tissue-specific promoter and enhancer evolution in mammals is partly shaped by transposable elements, which can contribute novel transcription factor binding sites [23]. To date, most studies have focused on the regulatory contribution of the endogenous retrovirus (ERV) superfamily of the long terminal repeat (LTR) subclass [24–26] and the short interspersed nuclear element (SINE, sometime represented as Short INterspersed Element) superfamily of the non-LTR subclass [19, 27–29]. Less is known about regulatory contributions of the long interspersed nuclear element (LINE, sometimes represented as Long INterspersed Element) superfamily, which makes up around 20% of mammalian genomes [30]. Both LINE L1s and L2s evolved before the mammalian radiation, although the L2 family is more ancient [31]. In many mammalian genomes, L1s are the only elements still actively retrotransposing [32]. L1s are often transcribed in a cell-type-specific manner [33–36] and there is limited evidence for their direct contribution to gene regulation [37]. In human cells, LINE L2s are expressed as miRNAs [38] and may have regulatory activity [29, 39], but it is unknown whether L2s play a regulatory role in other mammalian lineages.

Here, by comparing the epigenetic and transcriptional landscapes of multiple tissues and species across nearly 160 million years of mammalian evolution, we revealed new insight into the molecular mechanisms underlying tissue-specific and tissue-shared regulatory evolution. Our analyses demonstrated how promoters and enhancers can interchange regulatory signatures between species and discovered how different LINE families help shape tissue-specificity and regulatory signatures.

## Results

### Mapping regulatory evolution across four tissues in ten mammals

The species selected for mapping active regulatory regions represent several mammalian clades including primates (macaque and marmoset), Glires (mouse, rat, and rabbit), Laurasiatheria (pig, horse, cat, and dog), and marsupials (opossum) (Additional file 1: Table S1); all species have high-quality reference genomes with extensive annotation [40].

**Fig. 1:**
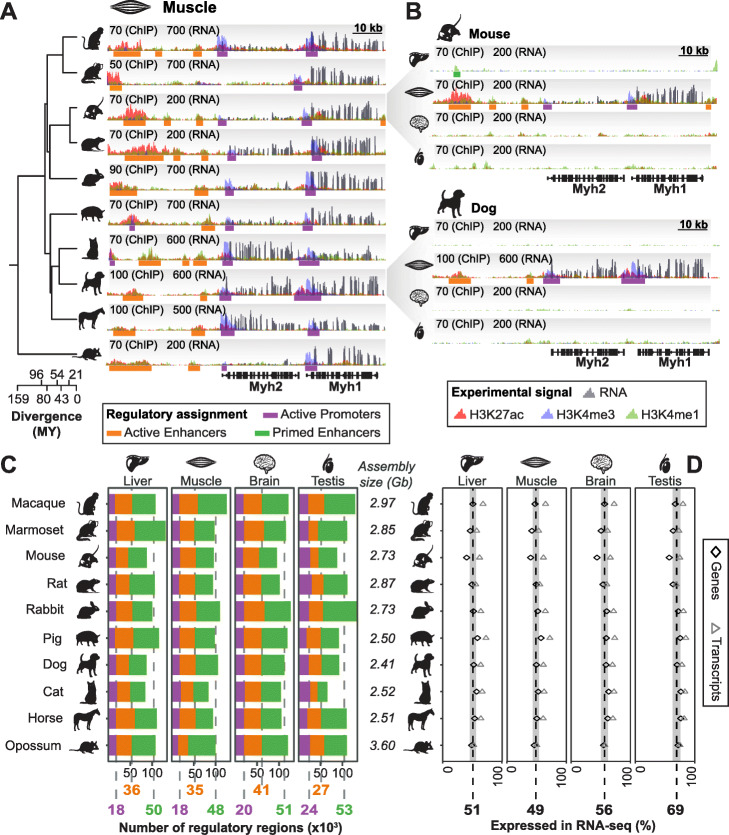
Promoter, enhancer and gene expression mapping demonstrates consistent tissue-level gene regulation in mammals. **a** Example conserved, tissue-specific regulatory landscape around Myosin Heavy Chain 1 and 2 genes (*Myh1* and *Myh2*) in muscle tissue from ten mammalian species. Inset numbers are maximum read depths for ChIP-seq and RNA-seq, while phylogenetic relationships and species divergences are shown on the left (see Additional file 1: Figure S1 for experimental workflow). **b** For the same locus as in **a**, regulatory landscapes in the liver, muscle, brain, and testis are shown for mouse and dog. **c** For each tissue, the number of biologically reproducible regulatory regions identified is consistent across species. Within a species, data is shown as stacked bar charts and the cross-species averages are similarly stacked below the graph. The average number of active promoters (purple), active enhancers (orange), and primed enhancers (green) across all species is shown below each column. The size of the underlying assemblies in Gigabases (Gb) is shown for all species. **d** The fraction of genes (diamonds) and transcripts (triangles) expressed in each tissue is stable across ten species. The average percentage of expressed genes across all species is shown as a dotted line with the confidence interval corresponding to +/− one standard deviation shaded. Species with larger differences between the fraction of expressed genes and transcripts have more comprehensive annotation

We profiled the regulatory landscape of adult liver, muscle, brain, and testis. Samples taken from these organs represent three somatic tissues originating from distinct developmental germ layers and one germline tissue. In each tissue, matched functional genomics experiments were performed in biological triplicate (with one exception, see the “Materials and methods” section, Additional file 2: Table S2). Chromatin immunoprecipitation followed by high-throughput DNA sequencing (ChIP-seq) was used to map three histone modifications associated with regulatory activity: histone 3 lysine 4 trimethylation (H3K4me3), histone 3 lysine 4 monomethylation (H3K4me1), and histone 3 lysine 27 acetylation (H3K27ac) (Fig. 1a, b; Additional file 1: Figure S1). Libraries were sequenced to saturation: 20 million reads were sufficient to saturate the signal for H3K4me3 and H3K27ac across all tissues, while 40 million reads were needed to saturate H3K4me1 signal (Additional file 1: Figures S1 and S2A; Additional file 3: Table S3; Materials and methods). We called peaks for each replicate with MACS2 and kept those enriched for each mark based on reported q-value (Materials and methods). All following analyses used highly reproducible peaks present in at least two biological replicates (Additional file 1: Figures S1 and S2B).

Within each tissue, active promoters were defined as regions enriched for both H3K4me3 and H3K27ac [41, 42] (Additional file 1: Table S4; Figure S1; Fig. 1a and b). Active enhancers were defined as regions enriched for both H3K4me1 and H3K27ac, but not H3K4me3 [41, 43]. Primed enhancers, or intermediate enhancers, were defined as regions enriched for H3K4me1 only [44, 45]. These are thought to be “primed” with H3K4me1 and may become readily active in response to specific stimuli [46]. The median of the H3K27ac peak enrichment is lower for active enhancers than for active promoters although the distributions overlap (Additional file 1: Figure S2C). A similar trend was observed for H3K4me1 enrichment distributions for active and primed enhancers with greater overlap (Additional file 1: Figure S2C).

To quantify genome-wide transcriptional activity, we generated matched total RNA-seq for the same samples used to map histone modifications (with rare exceptions, see the “Materials and methods” section, Additional file 2: Table S2). RNA-seq libraries were generally sequenced to a minimum of 40 million mapped reads for somatic tissues and 100 million for testis (Additional file 1: Figure S1; Additional file 4: Table S5). We used these data to improve the publicly released Ensembl genome annotations for eight species (Materials and methods) [40, 47].

From these nearly 500 matched experiments across four adult tissues and ten mammalian species, we annotated more than 2.8 million regulatory regions. This dataset captured a substantial proportion of known regulatory regions genome-wide and identified thousands of novel regulatory regions in each tissue (Additional file 1: Figures S3A, B and C). This dataset provides a comprehensive and consistent resource for inter-tissue and inter-species analyses of regulatory evolution, especially for species that have not been extensively studied.

### Tissue-level regulatory and transcriptional landscapes are consistent across mammals

The number of regulatory regions identified for each tissue using consensus peaks (Additional file 1: Figure S1 and Figure S2B) was largely consistent across species (Fig. 1c). Liver and muscle are relatively homogeneous somatic tissues consisting mostly of hepatocytes and myocytes, respectively. Each of these two tissues expressed approximately half of all annotated genes (Fig. 1d) and had on average 18,000 active promoters, 36,000 active enhancers, and 49,000 primed enhancers (Fig. 1c). In the brain, we identified more active regulatory regions on average: 20,000 active promoters and 41,000 active enhancers. This increase is consistent with the higher gene expression we observed (56% of genes are transcribed), as well as with the greater cellular heterogeneity of brain [48]. Indeed, the number of regulatory regions we identified in whole brain was comparable to the combined total found from profiling individual primate brain regions [49], suggesting that we effectively captured the brain regulome (Materials and methods). Consistent with previous reports, there were twice as many active enhancers as active promoters for all three somatic tissues [14, 41].

Testis is distinct from somatic tissues in that it is primarily composed of germ cells at different stages of spermatogenesis [50]. Testis had more active promoters compared to other tissues (24,000; Fig. 1c) and expressed the highest portion of annotated genes and transcripts (69%, Fig. 1d), consistent with known testis transcriptome diversity [50, 51]. Testis also had a lower ratio of enhancers to promoters compared to somatic tissues. Overall, testis regulatory regions were similarly enriched to those in other tissues (Fig. 1c; Additional file 1: Figures S2A and B) albeit with fewer average H3K4me3 reads per active promoter because of a larger number of promotors and our decision to use the same number of reads across tissues (Additional file 1: Figure S4). Additionally, the H3K27ac enrichment was comparable between the somatic tissues and the testis (Additional file 1: Figure S4). Thus, the distinct regulatory landscape of testis is not the result of technical differences.

Taken together, we found that promoter and enhancer landscapes correspond to gene expression, depend on tissue identity, and are consistent across species.

### Distinctive regulatory landscapes characterize somatic tissues and testis

**Fig. 2:**
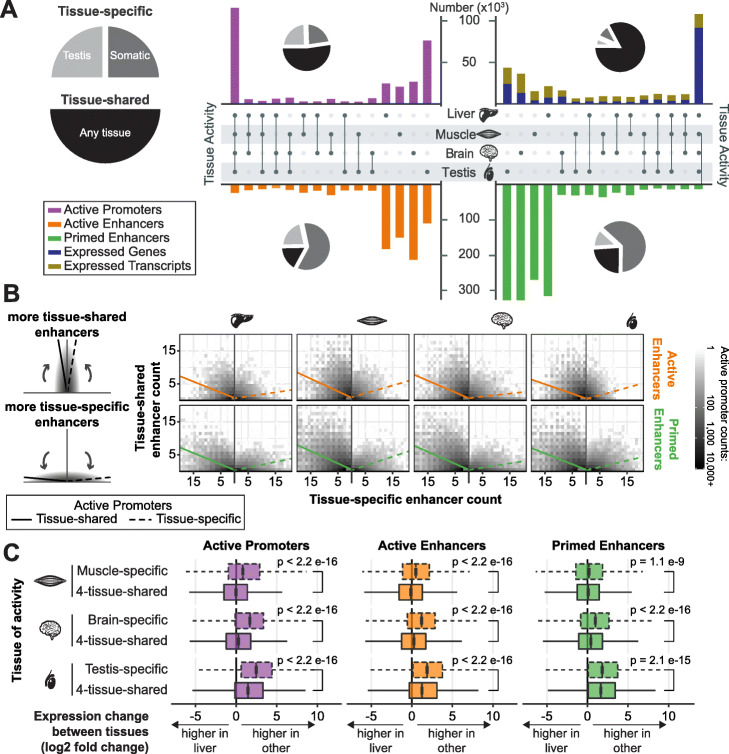
Tissue-specific enhancers are associated with tissue-specific and tissue-shared promoters. **a** Within each species, promoter activity and gene expression were distributed between tissue-specific and tissue-shared, while enhancer activity was mostly tissue-specific (see Additional file 1: Figure S5). The bars are a summation of numbers across all ten study species while and the pie charts show the portions of tissue-shared (across any two tissues) and tissue-specific regions split by testis-specific and somatic-specific; mouse as a representative species is shown in Additional file 1: Figure S6A. **b** Numbers of tissue-shared (*y*-axis) and tissue-specific (*x*-axis) enhancers associated with each active promoter are shown for the four tissues. The schematic (right) represents two extreme examples: promoters predominantly associated with tissue-shared enhancers (top) or tissue-specific enhancers (bottom). Tissue-shared promoters (left panels) are associated with a higher ratio of tissue-shared versus tissue-specific enhancers; whereas tissue-specific promoters (right panels) are predominantly associated with tissue-specific enhancers. A linear regression was fitted to the ratio of tissue-specific to tissue-shared enhancers per promoter and is shown as a line. For a distribution of all the tissue-specific to tissue-shared ratios see Additional file 1: Figure S6B. **c** Observed expression changes between tissues for genes associated with regulatory regions in muscle, brain, and testis. The plots show the distribution of differential expression with liver as a reference (DESeq2 adjusted *p* value < 0.05), of genes nearest to 4-tissue-shared regulatory regions and tissue-specific regulatory regions (*p* values calculated using one sided Wilcoxon test; tissue-specific expression change is greater than tissue-shared)

Within each species, we analyzed the tissue-specificity (Fig. 2a; Additional file 1: Figure S6A) of enhancers, promoters, and gene expression and then combined these into an overview for all species (Fig. 2a). Consistent with previous studies [41, 52], enhancers were mostly tissue-specific: 76% of active enhancers and 83% of primed enhancers were found in only one of the four tissues profiled. The largest group of active promoters were shared across all four tissues (37%; Fig. 2a) and almost half of active promoters were tissue-specific, split between those that are testis-specific or specific to any of the three somatic tissues (25% and 23%, respectively). The tissue-specificity of transcripts mirrored that of active promoters. The numbers of genes and the numbers of transcripts expressed in all four tissues were similar. In contrast, the number of tissue-specific expressed transcripts was 2–4 times higher than tissue-specific expressed genes, and more closely matched the number of active promoters (Fig. 2a). This trend is especially evident in mouse, where the annotation is most comprehensive (Additional file 1: Figure S6A). This suggests that tissue-specific promoters modulate alternative transcript usage.

We investigated the association between promoters and enhancers by assigning enhancers to the nearest promoter within 1 Mb, in line with studies that have shown that a substantial majority of enhancers act on the nearest gene [53, 54] (Materials and methods). We examined ratios of the number of tissue-specific and tissue-shared enhancers for each active promoter. Tissue-shared active promoters typically associated with both tissue-shared enhancers and a larger number of tissue-specific enhancers (Fig. 2b, left), reflecting the overall tissue-specificity of enhancers (Fig. 2a). In contrast, tissue-specific active promoters associated with fewer tissue-shared enhancers and more tissue-specific active enhancers (Fig. 2b, right), compared to tissue-shared promoters. Both active and primed enhancers showed similar, and statistically significant, trends (Additional file 1: Figure S6B, *p* value < 2.2e^−16^). In testis, tissue-specific promoters associated with fewer tissue-specific active enhancers than did tissue-specific promoters in somatic tissues (Fig. 2b), reflecting the overall fewer active enhancers active in testis (Figs. 1c, 2a).

We assigned regulatory regions to their nearest gene and compared gene expression levels across tissues using liver as a reference (Fig. 2c). Genes near tissue-shared regulatory regions showed similar expression levels across somatic tissues. In contrast, genes near muscle- and brain-specific regulatory regions had significantly higher expression in those tissues than in liver (*p* values < 1.1e^−9^). This effect was strongest for promoters and is evident for enhancers (Fig. 2c). For testis, genes associated with tissue-shared regulatory regions are more highly expressed than in liver (Fig. 2c). Additionally, genes associated with testis-specific regulatory regions are significantly more expressed in testis than genes associated with tissue-shared regulatory regions (*p* values < 2.1e^−15^).

These results demonstrate that regulatory landscapes differ between somatic tissues and testis and that the number of tissue-specific active promoters corresponds to the number of genes with tissue-specific expression.

### Tissue-shared promoters and enhancers display enhanced evolutionary stability

**Fig. 3:**
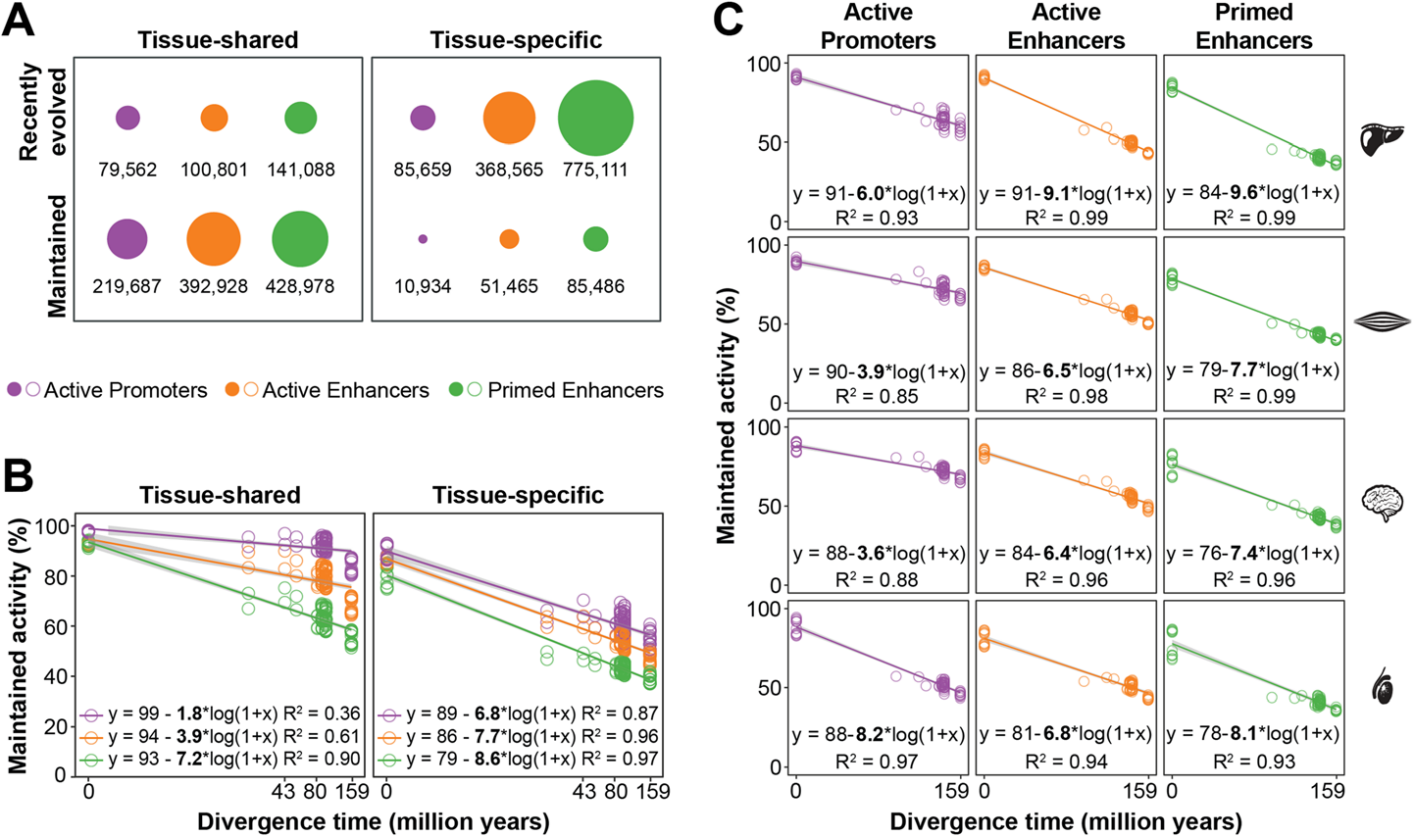
Tissue-specific regulatory regions have higher evolutionary turnover than tissue-shared regions. **a** The number of tissue-shared and tissue-specific regulatory regions that are either maintained or recently evolved across all ten species (see Additional file 1: Figure S5). The majority of tissue-shared regulatory regions are maintained across species: 73% of active promoters, 80% of active enhancers, and 75% of primed enhancers. The majority of tissue-specific regions are recently evolved, although 11% of active promoters, 12% of active enhancers and 10% of primed enhancers are maintained. **b** Evolutionary rates of alignable tissue-shared and tissue-specific regulatory regions estimated by linear regression of activity maintenance between all pairs of species and zero points estimated from interindividual variation (Materials and methods). For tissue-shared regions, the slope of the regression line for active promoters is lower than that of active enhancers or primed enhancers (two-way ANOVA of linear regression: active promoters vs active enhancers *p* value 0.0063; active promoters vs primed enhancers *p* value 0.0056). For all tissue-specific regions, the rates of evolution are either indistinguishable or greater than that for tissue-specific primed enhancers (two-way ANOVA of linear regression: active promoters slope vs primed enhancer slope, *p* value 0.011; active enhancers slope vs primed enhancers slope, *p* value 0.021). **c** Evolutionary rates of tissue-specific regulatory regions further stratified by tissue of activity. The slope of the regression line for testis-specific active promoters is significantly higher than for promoters with activity specific to the liver, muscle, or brain (two-way ANOVA of linear regression: testis-specific active promoters vs all other tissue-specific active promoters *p* value 3 × 10^−8^). However, all tissue-specific promoters evolve more rapidly than tissue-shared promoters, regardless of their tissue of activity (two-way ANOVA of linear regression: all tissue-specific active promoters (Fig. 3b) vs tissue-shared active promoters (Fig. 3b) *p* value 4 × 10^−8^)

The association between tissue specificity and evolutionary stability of enhancers and promoters has remained largely unexplored. Previous work in single tissues demonstrated that few enhancers are conserved across mammals [14, 41], and those conserved are more likely to be active in multiple cellular contexts [55]. Here, we exploited matched enhancer and promoter landscapes to identify evolutionarily maintained and recently evolved regulatory regions (Fig. 3a; Additional file 1: Figure S5; Materials and methods). Across all ten species, we found 1.6 million recently evolved regulatory regions and 1.2 million maintained regulatory regions. Most tissue-shared regulatory regions (76%) were maintained in evolution across two or more study species, although there were also many tissue-shared regions that were recently evolved. In contrast, most tissue-specific regulatory regions were recently evolved (89%; Fig. 3a).

We quantified evolutionary rates for tissue-shared and tissue-specific regulatory regions. Through pairwise comparisons of alignable regions, we calculated the fraction of promoters and enhancers maintained between each pair of species, and then used the slope of a linear fit to estimate the evolutionary rate of change (Fig. 3b, Materials and methods). Studies in single mammalian tissues have found that enhancers evolve more rapidly than promoters, but without differentiating between active and primed enhancers [14, 18]. We demonstrate that primed enhancers evolve much faster than active enhancers for both tissue-shared and tissue-specific regulatory elements.

More importantly, we consistently found that tissue-specific regulatory regions evolved more rapidly than their tissue-shared counterparts (Fig. 3b, *p* value < 0.021). This result is unaffected by enrichment of the histone modifications used to define the regions (Additional file 1: Figure S7A). Interestingly, tissue-specific active promoters evolved at rates comparable to enhancers, which may partly explain previous observations of fast rates of transcription start site evolution [19, 22].

We then asked whether regulatory regions evolve faster in particular tissues (Fig. 3c). Among promoters, those with testis-specific activity evolved most quickly, followed by liver-specific ones. In contrast, among both active and primed enhancers, those with liver-specific activity were the fastest evolving. Brain-specific regulatory regions evolved the most slowly (Fig. 3c). These tissue-specific rates of regulatory evolution give new insight into previously reported gene expression evolution rates, which found relatively small changes in brain and accelerated evolution in testis and liver [11, 12]. Our results suggest that both enhancers and promoters underlie the previously observed evolutionary rates of gene expression across tissues.

In sum, tissue-shared regulatory activity is a trait predictive of slower evolutionary turnover, regardless of the class of regulatory region or tissue of activity.

### Regions that switch promoter and enhancer signatures within a species are uncommon and are not evolutionarily maintained

**Fig. 4:**
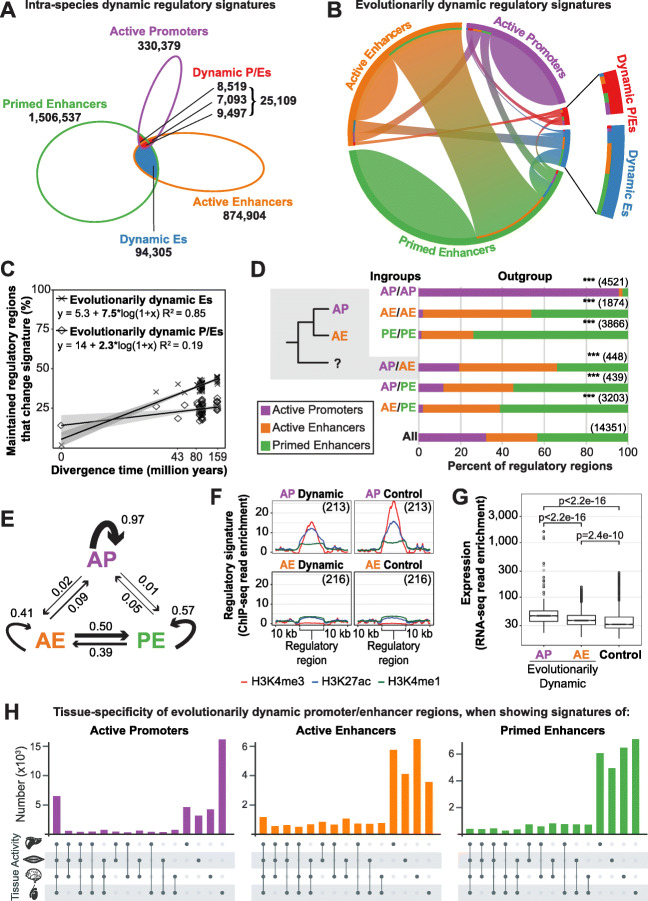
Promoter and enhancer signature is highly dynamic across species, but not within species. **a** Within a species, dynamic P/Es (red) were regions identified as an active promoter in one tissue and an enhancer in another tissue, and dynamic Es (blue) were an active enhancer in one tissue and primed in another. Numbers for each category are summed across all ten species. Within a species and across tissues, only 4% of the regulome is composed of intra-species dynamic regions. **b** Pairwise comparisons between maintained regulatory regions show how often regulatory signature changes between species. A substantial proportion of regulatory regions align to a region with a different regulatory signature in another species: 20% of pairwise comparisons with active promoters, 58% with active enhancers and 40% with primed enhancers are evolutionarily dynamic. Almost half of active enhancers (44%) aligned to primed enhancers. Dynamic P/Es (red) and dynamic Es (blue) almost always align to non-dynamic categories in other species (73% and 88% respectively), illustrating the evolutionary instability of this regulatory assignment. An enlargement of the intra-species dynamic regions is shown on the right for clarity. For changes of regulatory changes within the same tissue between species see Additional file 1: Figure S8B. **c** Evolutionary rates of changing regulatory signatures among maintained regulatory regions estimated by linear regression of pairwise comparisons. Across evolution, maintained active promoters (crosses) and active enhancers (diamonds) were more likely to change regulatory signature as evolutionary distance between species increased. **d** Evolutionary directionality of dynamic regulatory signatures estimated by outgroup analysis of mouse/rat/rabbit and cat/dog/horse triads. Gray inset example: in 448 cases when a genomic region is an active promoter in one ingroup species and an active enhancer in the other, the outgroup species was most likely to be an active enhancer (46%), and least likely to be an active promoter (20%). The distributions of outgroup active promoters, active enhancers and primed enhancers for each ingroup combination is statistically different from the background (All) distribution (chi-square two-tailed test, ***p < 0.001). Outgroup analysis was performed separately for each triad group, and then combined (see Additional file 1: Figure S8C and D). **e** Composite model based on the observed likelihood of regulatory regions changing or maintaining regulatory signatures over evolution. The thickness of the lines reflects the relative likelihood of evolutionary change, as calculated from the most parsimonious evolutionary relationships from the triad data in **d** and normalizing the outgoing lines from each state to one. **f** Validation of regulatory signature assignment using the average ChIP-seq read enrichment for evolutionarily dynamic regulatory regions and equal numbers of randomly selected control regions. Dynamic regions were the AP/AE ingroup regions identified as AE in the outgroup analysis in **d**. Total number of regions used are shown as insets. **g** Distribution of RNA-seq read counts for evolutionarily dynamic active promoters (AP) and active enhancers (AE) shown in **f**, and equal numbers of randomly selected control active enhancers that are not evolutionarily dynamic (*p* values calculated using one sided the *t*-test for greater expression). **h** Tissue distribution of evolutionarily dynamic P/Es in the species where they were an active promoter, active enhancer or primed enhancer. When showing signatures of active promoters (left; purple), they were less likely to be tissue-shared and more likely to be testis-specific than all promoters (compared to Fig. 2b). When showing enhancer signatures, they were more likely to be tissue-shared than all enhancers (Fig. 2b bottom orange and green)

Some genomic regions can act as either promoters or enhancers in different contexts [4], but the evolutionary turnover and maintenance of such dynamic regulatory regions have not been evaluated. We defined intra-species dynamic regulatory regions as those with differing histone modification signatures across tissues within a single species (Additional file 1: Figure S5; Fig. 4a; Materials and methods). Regions identified as active promoters in one tissue and active and/or primed enhancers in another tissue were defined as intra-species dynamic promoter/enhancers (dynamic P/Es). Similarly, regions identified as active enhancers in one tissue and primed enhancers in another were defined as intra-species dynamic enhancers (dynamic Es). Between the four tissues, only a small portion of each species’ regulome was intra-species dynamic on average: 7% of active promoters, 11% of active enhancers, and 7% of primed enhancers (Fig. 4a).

We compared the evolutionary rates of intra-species dynamic P/Es and dynamic Es with that of typical promoters and enhancers (Additional file 1: Figure S7B; Materials and methods). Dynamic P/Es had a higher evolutionary rate than tissue-shared active promoters or active enhancers and were more maintained than tissue-specific active promoters or active enhancers. Similarly, dynamic Es had a higher rate than tissue-shared active enhancers and were more maintained than tissue-specific active enhancers or primed enhancers. Thus, the evolutionary stability of dynamic regulatory regions is between that of their tissue-shared and tissue-specific counterparts.

We investigated the evolutionary stability of intra-species dynamic P/Es and dynamic Es by asking how often they aligned to a similarly dynamic region in another species (Fig. 4b; Additional file 1: Figure S8B; Materials and methods). The majority of intra-species dynamic P/E alignments were to non-dynamic regions in other species (73%) with approximately equal numbers aligning to active promoters, active enhancers, and primed enhancers. Similarly, most alignments that included intra-species dynamic Es (80%) were either to active or primed enhancers, with only 12% aligning to another dynamic E.

In sum, the dynamic regions that switch promoter and enhancer signatures between tissues within one species are relatively rare and are not maintained as intra-species dynamic regions across species.

### Promoter and enhancer signature dynamics are common between species

The functional signatures of regulatory regions may also change across species. Indeed, prior studies have identified a limited set of genomic regions that switch between promoter and enhancer signatures within primates or rodents [10].

We thus investigated the evolutionary stability of histone modification signatures for all pairwise comparisons between species where regulatory activity is maintained (Additional file 1: Figure S5; Materials and methods). For example, we asked how often an active promoter in mouse aligns to an active enhancer in dog—regardless of the tissue of activity. Active promoters were the most stable regulatory class across evolution: for those that were maintained as a regulatory region across species, 80% of pairwise comparisons were identified as promoters in both species (Fig. 4b). The two classes of enhancers were less stable across evolution: only 42% and 60% of pairwise comparisons involving active and primed enhancers, respectively, retained the same enhancer signature between the two species.

Similar to the intra-species dynamic regions, we defined evolutionarily dynamic regions as those with different regulatory signatures between species (Additional file 1: Figure S5). We found that evolutionarily dynamic regions were more common than intra-species dynamic regions. Specifically, 15% of pairwise comparisons involving promoters were evolutionarily dynamic (Fig. 4b) compared to only 7% intra-species dynamic (Fig. 4a). For enhancer comparisons, 44% of active enhancers aligned to a primed enhancer in another species (Fig. 4b), compared to 10% of active enhancers in one species identified as primed enhancers in a different tissue (Fig. 4a). Indeed, almost half of active enhancers were readily interchangeable with primed enhancers across ten mammals, suggesting that enhancer states are in an approximate evolutionary balance.

To explore whether histone modification enrichment influences the evolutionary stability of regulatory signatures, we compared the enrichment of peak calls underlying evolutionarily dynamic and stable regulatory regions (Additional file 1: Figure S8A; Materials and methods). Evolutionarily dynamic active promoters are weaker than their stable counterparts, active enhancers are similar, and primed enhancers are stronger when evolutionarily dynamic. Thus, ChIP enrichment alone cannot explain evolutionary changes between regulatory signatures.

We next limited our evolutionary stability analyses to only those regulatory regions maintained within the same tissue across evolution (Additional file 1: Figure S8B). For evolutionary dynamic regions within the same tissue, a comparable number of active promoters (75–78% of pairwise comparisons; Additional file 1: Figure S8B) are stable compared to those stable between any tissue (80% of pairwise comparisons; Fig. 4b). Enhancers are more stable when only considering evolutionary dynamic regions within the same tissue: 45–49% of active enhancers and 43–45% of primed enhancers (Additional file 1: Figure S8B) are stable, compared to 42% and 60%, respectively, when considering changes between any tissue (Fig. 4b). Together, these results demonstrate that regulatory signature changes do occur within the same tissue across evolution and indicate that enhancers are more dynamic than promoters.

We investigated whether regulatory regions were more likely to change signature with increasing evolutionary distance. We calculated the proportion of maintained promoters that switch between active promoter and any enhancer (evolutionarily dynamic P/Es; Additional file 1: Figure S5) as well as the proportion of maintained active enhancers that switch between active and primed enhancers (evolutionarily dynamic Es; Additional file 1: Figure S5). The proportion of maintained active promoters and active enhancers changing regulatory signature and becoming evolutionarily dynamic increased with greater evolutionary distance (Fig. 4c). The rate of switching between active and primed enhancers was higher than between promoters and enhancers (Fig. 4c). With this, we have quantified two evolutionary trajectories of regulatory regions: the rate of overall loss of regulatory regions (Fig. 3b) and the frequency at which maintained regions change their regulatory signature across species (Fig. 4c).

To examine directionality of changing regulatory signatures, we focused on species in our phylogeny with shorter evolutionary distances and clear ingroup and outgroup relationships. We separately investigated mouse and rat with rabbit as outgroup, and cat and dog with horse as outgroup. For both trios, we considered only regulatory regions that were maintained across all three species. For each regulatory region, we determined the regulatory signature in the outgroup species given the signatures in the two ingroup species, regardless of the tissue of activity (Additional file 1: Figure S5). As expected, when a genomic region was defined as an active promoter in both ingroup species, it was also defined as an active promoter in the outgroup 95% of the time (Fig. 4d; Additional file 1: Figures S8C and D).

When a genomic region was consistently identified as an active enhancer in both ingroup species, it was a primed enhancer 46% of the time in the outgroup (Fig. 4d; Additional file 1: Figures S8C and, D). Correspondingly, when a region was identified as a primed enhancer in both ingroup species, it was an outgroup active enhancer 25% of the time. These results further demonstrate that active and primed enhancers are readily interchangeable throughout evolution. Similarly, when a region was defined as an active enhancer in one ingroup species and a primed enhancer in the other, it was identified as an outgroup active enhancer 37% of the time and as a primed enhancer 61% of the time. This suggests that for evolutionarily dynamic Es, the ancestral state is almost twice as likely to be a primed enhancer than an active enhancer. Thus, primed enhancers are more likely to evolve into active enhancers than the reverse, yet both types of changes are widespread.

### Promoters often arise from ancestral enhancers

Our data enabled us to quantitatively investigate the suggested model that promoters arise from ancestral enhancers [10]. Regions identified as an active promoter in one ingroup species and an active or primed enhancer in the other were identified as an enhancer in the outgroup species more than 80% of the time (Fig. 4d; Additional file 1: Figures S8C and D). The similar contribution of active and primed enhancers in the outgroup is likely due to their rapid evolutionary interchange. At these evolutionary distances, promoters arise from enhancers six times more often than enhancers arise from promoters.

We used the frequency of regulatory signature change observed in the outgroup analysis to model regulatory signature evolution (Fig. 4e; Materials and methods). Our model predicts that active promoters are most likely to maintain their signature, and primed enhancers are about as likely to evolve to active enhancer signatures as they are to remain primed. Active enhancers have two equally likely evolutionary fates: maintaining their signature or evolving into primed enhancers.

Finally, we validated the evolutionary switching of regulatory signatures from enhancers to promoters by examining the enrichment of histone modifications and transcription around evolutionarily dynamic P/Es identified in the outgroup analysis (Fig. 4e; Additional file 1: Figures S8C and, D). We used parsimony to select regions that were most likely to represent evolutionary switches from active enhancer to active promoter (ingroups: active promoter, active enhancer; outgroup: active enhancer), and compared the ChIP-seq and RNA-seq read enrichment between regions marked as active promoters and active enhancers in the ingroups. The regions showed characteristic chromatin signatures of active enhancers and active promoters (Fig. 4f). Furthermore, ingroup active promoters had increased transcription of flanking regions compared to ingroup active enhancers (Fig. 4g), indicating that the regulatory signature change leads to higher transcriptional activity.

### Evolutionarily dynamic promoters are more likely to be tissue specific

We initially defined evolutionarily dynamic P/E regions without considering their tissue of activity (Additional file 1: Figure S5). We examined the tissue-specificity of these regions and compared it to the overall tissue-specificity pattern for promoters and enhancers (Fig. 2b). For each region, we separately characterized the tissue-specificity in species where it showed signatures of an active promoter, active enhancer, or primed enhancer (Fig. 4h). In species where evolutionarily dynamic regions had an enhancer signature, they were mostly tissue-specific, similar to the trend for all enhancers (Fig. 2b) and were only slightly more likely to be tissue-shared than all other enhancers (24% evolutionarily dynamic enhancers active across more than two tissues, compared to 20% of all enhancers; binomial test *p* value < 2.2*10^−16^). When evolutionarily dynamic regions showed promoter signatures changes to tissue-specificity were more pronounced, with only 16% of them being active across all four tissues (Fig. 4h) compared to 37% of all promoters (Fig. 2b; binomial test *p* value < 2.2*10^−16^). Interestingly, in the species where evolutionarily dynamic P/Es had promoter signature, 41% were testis-specific (Fig. 4h), which is significantly higher than the 25% observed for all promoters (Fig. 2b; binomial test *p* value < 2.2*10^−16^). These results indicate that evolutionarily dynamic promoters change both regulatory signature and tissue-specificity between species.

### LINEs are a versatile source of regulatory activity

**Fig. 5:**
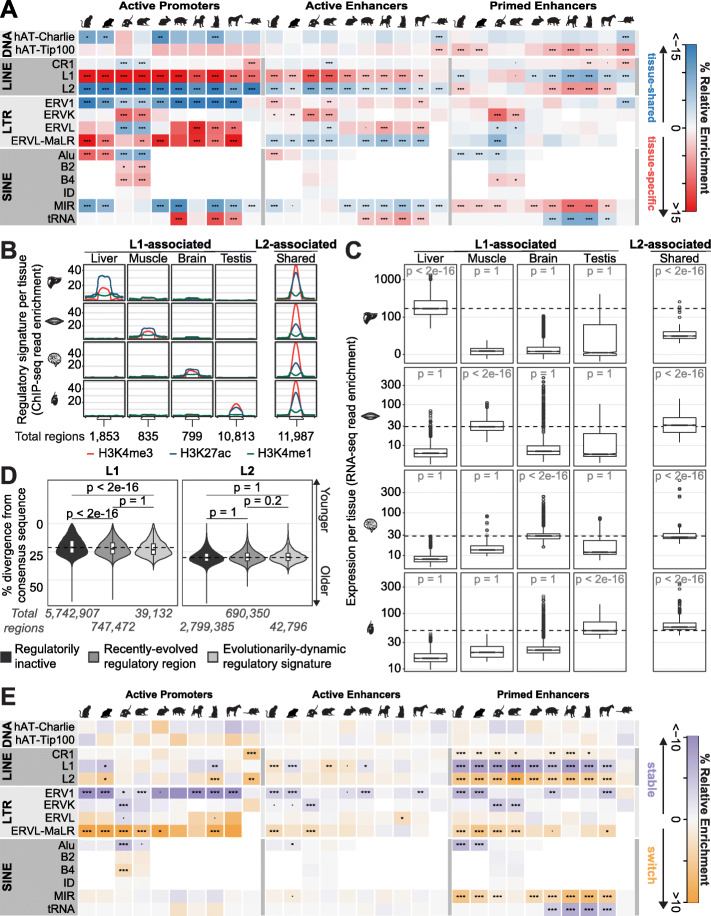
Distinct families of repetitive elements contribute to recently evolved and maintained regulatory regions. **a** Relative enrichment of recently evolved tissue-shared versus tissue-specific regulatory regions for selected transposable element families shown as a heatmap. Within each family, significance of tissue-specific vs. tissue-shared proportions calculated with the z-test and Bonferroni correction (*p* values ***< 0.001; **< 0.01; *< 0.05; − < 0.1. See Additional file 1: Figure S9B for maintained regions). **b** Validation of tissue-specific activity using the average ChIP-seq read enrichment for recently evolved active promoters associated with LINE L1s and L2s and their flanking regions. **c** Distribution of RNA-seq read counts for the promoter flanking regions in **b**. Dotted lines represent the median of tissue-specific RNA-seq enrichment for the tissue profiled. *P* values calculated using one sided the Wilcoxon test; within row test if read counts in each LINE-associated region type (column) is greater than in all other regions. **d** Estimated age of LINE L1s and L2s, as inferred by the number of substitutions from consensus sequence. LINE L1s that overlap regulatory regions (medium and light gray) are significantly older than inactive L1s (dark gray), while regulatorily active L2s are of similar age to inactive L2s. Dotted line is the median % divergence of the corresponding regulatorily inactive LINEs and *p* values calculated using one sided the Wilcoxon test for greater sequence divergence. Divergence is shown for all ten species combined; see Additional file 1: Figure S5B for per-species divergences. **e** Heatmap of relative enrichment in transposable element families for regulatory regions with evolutionarily dynamic (switch) versus stable signatures. Within each family, significance of evolutionarily dynamic vs. stable proportions calculated with the z-test and Bonferroni correction (*p* values ***< 0.001; **< 0.01; *< 0.05; − < 0.1)

We next asked how specific classes of repeat elements contribute to the evolution of tissue-specific and tissue-shared regulatory activity across mammals. We separately analyzed recently evolved and maintained regulatory regions (Fig. 3a) and identified which transposable elements they overlap. We grouped transposable elements into LINEs, SINEs, LTRs, and DNA transposons, and then compared the enrichment of annotated transposable element families between tissue-specific and tissue-shared regulatory regions (Materials and methods). Various families of transposable elements within the LTR and SINE groups such as Alu, B2, and ERVL elements contributed to tissue-specific and tissue-shared active promoters in a lineage-specific manner (Fig. 5a; Additional file 5: Table S6), in line with previous observations [26, 27]. These lineage-specific trends are not due to large differences in assembly completion: 98% of reads map to the finished quality mouse genome and, on average, 96% of reads to all other genomes (Additional file 3: Table S3).

Across all study species, we found that tissue-specific active promoters were enriched with LINE L1 family of transposons as compared to their tissue-shared counterparts despite regulatory regions not overlapping LINEs more than would be expected by chance (Additional file 1: Figure S9A). In contrast, tissue-shared active promoters were enriched in the LINE L2 family (Fig. 5a; Additional file 1: Figure S9A; Additional file 5: Table S6; Additional file 6: Table S7). This was observed for both recently evolved (Fig. 5a, Additional file 5: Table S6) and evolutionarily maintained (Additional file 1: Figure S9A; Additional file 6: Table S7) active promoters. The same trend of LINE L1 and L2 enrichment was observed in recently evolved and maintained active enhancers although the trend is weaker; this trend was not as evident for primed enhancers (Fig. 5a; Additional file 1: Figure S9A; Additional file 5: Table S6; Additional file 6: Table S7). On average 97% and 96% of quality-controlled reads (Materials and methods) mapped uniquely within LINE L1s and L2s (Additional file 7: Table S8) respectively, compared to 99% in non-repetitive genomic regions, indicating that the differences we observe are not due to mapping efficiency.

To gain insight into the impact of LINEs on transcription, we examined the histone modification enrichments (Fig. 5b) and gene expression (Fig. 5c) within 10 Kb of active regulatory regions overlapping LINEs. Recently evolved active promoters that were tissue-shared and contained L2s (7% of recently evolved promoters) had both high enrichment of H3K4me3 and H3K27ac and increased nearby transcription. The 9% of the recently evolved active promoters that were both tissue-specific and contained L1s showed enrichment only in the relevant tissue.

To assess transposable element contribution to regulatory signature dynamics, we compared their enrichment in evolutionarily dynamic P/E regions to those regions that retain stable regulatory signatures between species (Fig. 5e; Additional file 8: Table S9; Materials and methods). Among active enhancers, evolutionarily dynamic P/Es showed relative enrichment in the LINE L2 family compared to stable active enhancers. In contrast, stable active enhancers were enriched for the LINE L1 family. This trend is also evident for active promoters and primed enhancers in some lineages.

### Genomic characteristics of LINEs associated with regulatory regions

We investigated whether regulatory activity was associated with the evolutionary timing of LINE retrotransposition. The age of each LINE was estimated by its divergence from the consensus sequence. LINEs were divided into those that overlap identified regulatory regions and those that do not (Fig. 5d; Additional file 1: Figure S10A). As expected, LINE L2s were older than L1s regardless of regulatory association [31]. For all study species, the age of LINE L2s was similar for recently evolved tissue-shared regulatory regions, evolutionarily dynamic regions, and for L2s not associated with any regulatory activity.

LINE L1s that overlapped regulatory regions were significantly more diverged (Fig. 5d; *p* value < 2e^−16^) and thus older than those that were not regulatorily active. Specifically, regulatory regions that overlapped L1s were less likely to overlap the youngest L1s. This effect varied across species and was especially pronounced in rodents, primates, and opossum, where many L1s arose recently and have remained regulatorily inactive (Additional file 1: Figure S10A). Using the reported mutation rates of primate LINEs [56], we estimated that the expansion of L2s happened before the divergence of eutherian mammals (~ 100 million years ago), and the L1 expansion after the split, consistent with previous whole genome findings [57].

We sought evidence of selection in LINEs by examining their genomic characteristics. First, we compared the length of regulatorily active LINEs to those that were not regulatorily active (Additional file 1: Figure S10B). All LINEs, regardless of regulatory activity, are predominantly truncated forms of the full transposons. However, LINEs associated with recently evolved regulatory regions tend to be in longer fragments than regulatorily inactive ones suggesting selectional processes. For promoter-associated LINEs overlapping known transcription start sites, there is no correlation between LINE orientation and the direction of transcription (Additional file 1: Figure S10C), indicating that nearby transcription is not likely to be due to the transposable element’s pre-existing promoter. Next, we compared sequence constraint between tissue-specific regulatory regions overlapping L1s and L2s and found that both have constrained elements, but those overlapping L2s are significantly more constrained across their whole length (Additional file 1: Figure S10D; *p* value < 2e^−16^) and contain a larger number of constrained elements (Additional file 1: Figure S10E; *p* value < 2e^−16^). This suggests that lineage-specific genetic variation unmasks latent regulatory potential in existing LINEs.

Across the mammalian lineage, active regulatory regions consistently associated with LINE L1 transposable elements if they were tissue-specific, and with LINE L2s if they were tissue-shared (Fig. 5a; Additional file 1: Figure S9B). LINE L2s also consistently associated with evolutionarily dynamic regulatory regions (Fig. 5e), which frequently change both regulatory signature and tissue of activity, suggesting that LINE L2s provide a more versatile potential for transcriptional regulation than do LINE L1s.

## Discussion

Regulatory landscapes are composed of tissue-specific and tissue-shared regions that appear complex and evolutionarily unstable. We have created a comprehensive experimental dataset characterizing how tissue-specific transcriptional regulation has evolved from a common mammalian ancestor 159 million years ago. Using four adult primary tissues from ten species, we identified nearly 3 million regulatory regions and quantified the associated gene expression. Our analyses have given high-resolution insight into the evolutionary relationship between tissue-specificity and functional maintenance, characterized changing regulatory signatures across tissues and species, and revealed how LINE retrotransposons evolutionarily shape tissue-specificity.

Our analyses of the mechanisms of regulatory evolution between species and tissues have limitations. First, the four tissues we profiled do not represent all possible cell types, though the distinctive evolutionary mechanisms we have identified are likely robust, because our categorization of tissue-shared or tissue-specific is unlikely to substantially change with the addition of more cell types [18]. Second, our analysis does not capture all enhancers and promoters; like every method to define regulatory regions, it has specific advantages, disadvantages, and biases [2]. We used a widely-employed approach of combining three histone modification and performed all experiments in at least biological triplicates, yet this strategy cannot identify alternative promoters at high resolution as can techniques like CAGE [21]. Furthermore, H3K4me1, which differentiates active and primed enhancers, is more variable between replicates than other histone marks (Additional file 1: Figure S2A). Third, to fully explore how tissue-specific and tissue-shared regulomes interact to shape the evolution of gene expression would require the generation of high-resolution, three-dimensional contact data.

Although LINEs do not contribute to regulatory regions more than would be expected by chance genome wide, we were able to characterize their regulatory roles by comparing specific regions with each other including tissue-specific to tissue-shared and evolutionarily dynamic to stable. Indeed, we would expect to see greater evolutionary dynamism with a wider tissue or species sampling and thus a stronger association of LINE L2s with evolutionarily dynamic regions. We were not able to identify known motifs that can account for the observed differences between LINE L1s and L2s, which may be a consequence of shared mechanisms of activation for tissue-specific and tissue-shared regulatory regions. For example, a tissue-specific and tissue-shared promoter in liver, as well as any liver enhancer, would require many of the same transcription factors for activation.

### Regulatory roles change readily across evolution

Our results reveal that primed and active enhancers are frequently redeployed across evolution into different regulatory roles. Between tissues within a single species, only a small subset of promoters interchange regulatory roles with enhancers, in line with previous studies [3, 4]. Between species, there was suggestive evidence that ancestral enhancers can evolve to promoters in somatic tissues [10]. By analyzing a large number of species, characterizing a greater diversity of regulatory regions, and including a germline tissue, we discovered that changing regulatory roles is, in fact, a frequent event in mammalian evolution. One-fifth of alignments with maintained promoters and almost half of alignments with enhancers showed evidence of such interchange between species. The observed frequent evolutionary interchange of active and primed enhancers, both between all enhancers and those of comparable enrichment levels, may be the result of a birth-death balance, or potentially reflect a plasticity in the histone signatures of enhancers. We demonstrated that enhancers interchange regulatory signatures with promoters across evolution, most frequently with testis promoters. The distinct regulatory plasticity in testis supports a model wherein germline tissues have unique roles in evolution [58].

### LINE retrotransposons shape regulatory evolution across mammals

One of our most striking results is the opposing contributions of LINE L1s and L2s to regulatory evolution. Regulatorily active LINEs do not generally arise from lineage-specific insertions, suggesting that the predominant mechanisms for regulatory activation—including for those with lineage specific activity—are co-option of ancient elements. Multiple studies have characterized the contribution of lineage-specific insertions of transposable elements to regulatory evolution [24, 25, 28, 29]. In contrast, the regulatory potential of more ancient insertions of transposable elements has been less studied [27, 59]. LINE L1s are transcribed in a cell-type-specific manner [35], which corresponds to our findings that L1s are associated with tissue-specific regulatory activity. LINE L2s have been less studied, though recently shown to be ubiquitously expressed as miRNAs [38] and to have promoter and enhancer activity in human tissues [29].

Our data showed that LINEs—both L2s and L1s—are widely used across mammals as an evolutionary substrate for new promoter and enhancer regulatory activity. LINE L2s are associated with tissue-shared regulatory activity and evolutionarily dynamic promoter/enhancers. LINE L1s, in contrast, are associated with tissue-specific regulatory regions, as well as those with stable regulatory signatures that do not switch between promoter and enhancer regulatory signatures.

## Conclusions

By mapping the dynamic mammalian regulome across ten species, we reveal the complex, evolutionarily unstable regulatory landscapes underpinning stable tissue phenotypes and a role for ancient mammalian repeats in shaping their plasticity.

## Materials and methods

The published article includes all code generated or analyzed during this study in standalone ZIP file Additional file 9: Data S1.

### Species details

The ten species used in this study were rhesus macaque (*Macaca mulatta*), common marmoset (*Callithrix jacchus*), mouse (C57BL/6 J, *Mus musculus*), rat (Brown Norway, *Rattus norvegicus*), rabbit (*Oryctolagus cuniculus*), cat (*Felis catus*), dog (Beagle, *Canis familiaris*), horse (Welsh Mountain Pony, *Equus ferus*), pig (domestic pig, *Sus scrofa*), and gray short-tailed opossum (*Monodelphis domestica)*. All individuals used in this study were adults with no known health issues. Wherever possible, tissues from young adult males were used; however, some tissues were from females or older individuals. An overview of the origin, sex, and age for each animal used in the study is given in Additional file 1: Table S1. The details for each individual animal and tissue are given in Additional file 2: Table S2.

The use of all animals in this study was approved by the Animal Welfare and Ethics Review Board, under reference number NRWF-DO-02vs, and followed the Cancer Research UK Cambridge Institute guidelines for the use of animals in experimental studies. Tissues from seven species (macaque, marmoset, rabbit, cat, dog, horse, and opossum) were excess from routine euthanasia procedures (e.g., from individuals sacrificed during maintenance of research or breeding colonies). Tissues from three species (mouse, rat, and pig) were purchased commercially (e.g., from animal research supply companies.)

### Source and details of tissues

We performed ChIP-seq and RNA-seq on primary tissues isolated from 10 mammalian species. Primary tissues used were derived from the liver, skeletal muscle (from the upper hind leg), brain (whole), and testis. Brain samples were representative of the whole brain (see details below) for most animals, with the exception of macaque, in which some of the brain regions were not available (see Additional file 2: Table S2). At least three independent biological replicates from different animals were used, with the only exception being H3K4me3 from horse testis, in which two of the replicates were from the same individual (Additional file 2: Table S2). In most cases, matched tissues from the same individuals were used for all of the three ChIP-seq targets and RNA-seq (Additional file 2: Table S2).

Tissues were prepared immediately post-mortem, typically within an hour, to maximize experimental quality. Tissues were processed by extracting the organ, dicing the tissue to small pieces and mixing it to get a homogeneous mixture as a typical representation of the whole tissue, which was particularly important for whole brain samples. Tissues were either immediately snap-frozen on dry ice or liquid nitrogen for RNA-seq, or formaldehyde crosslinked (see below) and then frozen on dry ice for ChIP-seq.

### Chromatin immunoprecipitation and high-throughput sequencing (ChIP-seq)

Fresh, diced tissues were cross-linked in 1% formaldehyde in solution A (50 mM Hepes-KOH pH 7.5, 100 mM NaCl, 1 mM EDTA, 0.5 mM EGTA) for 20 min at room temperature, followed by the addition of 2.5 M glycine solution to a final concentration of approximately 250 mM glycine and incubated for a further 10 min at room temperature to neutralize the formaldehyde. Samples were washed with cold PBS then frozen on dry ice and stored at − 80 °C until use.

Tissues were homogenized by either dounce homogenization of thawed tissues in PBS (for softer tissues from smaller species), or by grinding frozen tissues with a Qiagen TissueLyser II and stainless steel grinding jars, keeping the samples frozen by cooling jars in liquid nitrogen (for tissues from larger species or for muscle). After homogenization, samples were stored at − 80 °C until use.

Chromatin immunoprecipitations were done in Nunc deepwell (1 ml) 96-well plates. Each plate was set up to contain chromatin from 24 different tissue samples—each split into three different ChIP reactions (H3K4me3, H3K4me1, and H3K27ac) plus input—for a total of 96 samples (72 ChIP reactions plus 24 inputs) per plate. As a result, all three ChIPs from the same tissue sample used the same input, except in cases where one of the ChIPs failed and needed to be repeated, in which case a new input was used for the new chromatin prep. Tissue samples were assigned to 96-well plates semi-randomly, while maintaining a fairly even representation of species and tissue-type per plate. Sample position on the plates was distributed in a semi-random fashion, while maximizing the distribution of samples with respect to species and tissue-type across the plate.

Antibodies used were H3K4me3 (Millipore 05-1339), H3K27ac (Abcam ab4729), and H3K4me1 (Abcam ab8895). Briefly, for each sample, 5 μg antibodies were pre-bound to 25 μL Protein G Dynabeads (Invitrogen) [60]. Sufficient Dynabeads and antibodies of the same type were pooled for all 24 tissue samples and incubated in 10 mL of block solution (1.5% BSA w/v in PBS) for at least 6 h at 4 °C. Immediately prior to setting up the ChIP reactions, after chromatin extracts were prepared (see below), the antibody-bound beads were washed with 3 × 10 mL block solution using a magnetic stand. Antibody-bound beads were then resuspended in block solution sufficient for 100 μL per sample and kept on ice.

Twenty-four samples at a time were lysed according to published protocols [60] to solubilize DNA-protein complexes. Typically 0.3 to 0.5 g of homogenized tissue was lysed and resuspended in a final volume of 3 mL prior to sonication. Homogenized tissue was resuspended in 10 mL of lysis buffer 1 (50 mM Hepes-KOH pH 7.5, 140 mM NaCl, 1 mM EDTA, 10% glycerol, 0.5% NP-40, 0.25% Triton X-100) and incubated with rotation for 10 min at 4 °C. Samples were centrifuged at 2500*g* for 3 min at 4 °C, and supernatants were discarded. The pelleted tissue was then resuspended in 10 mL of lysis buffer 2 (10 mM Tris-HCl pH 8.0, 200 mM NaCl, 1 mM EDTA, 0.5 mM EGTA) and incubated with rotation for 5 min at 4 °C. Samples were centrifuged at 2500 g for 3 min at 4 °C, and supernatants were discarded. Pelleted tissue was then resuspended in 3 mL lysis buffer 3 (10 mM Tris-HCl pH 8.0, 100 mM NaCl, 1 mM EDTA, 0.5 mM EGTA, 0.1% Na-Deoxycholate, 0.5% N-laurolsarcosine), transferred to a 5-mL Eppendorf tube, and incubated for at least 5 min (or up to 1 h) prior to sonication. Protease inhibitors (Complete, EDTA-free, Roche, #11873580001) were added to all lysis buffers immediately prior to use.

Chromatin was fragmented to 300 bp average size by sonication on a Qsonica Q500 with a 1/16″ microtip at 40% amplitude for a total sonication time of 6 min (12 cycles of 30 s on, 60 s off). After sonication, 10% Triton X-100 was added to each sample to bring the total concentration of Triton X-100 to 1%. Samples were spun at 16,000 g for 10 min at 4 °C, and the pellet was discarded.

Each chromatin extract was evenly split to perform three ChIP reactions: H3K4me3, H3K27ac, and H3K4me1. A small amount of extract (> 3 μL) was reserved and stored at 4 °C as input chromatin (see below). Chromatin (800 μL per well, corresponding to approximately 0.1 g of homogenized tissue) and antibody-bound-beads (100 μL of suspension, equivalent to 5 μg of antibody, per well) were loaded into a 96-well Nunc deepwell 1 mL plate, and incubated overnight at 4 °C with end-over-end rotation.

Washes and subsequent steps were carried out with an Agilent Bravo liquid handling robot according to published protocols [61]. Briefly, supernatant was discarded, and magnetic beads were washed 10x with 180 μL cold RIPA solution (50 mM Hepes-KOH pH 7.6, 500 mM LiCl, 1 mM EDTA, 1% NP-40, 0.7% Na-Deoxycholate), and then 2x with TBS. Magnetic beads were resuspended in 50 μL of elution buffer (50 mM Tris-HCl pH 8.0, 10 mM EDTA, 2% SDS) and incubated at 55 °C for 5 h in a thermocycler to reverse crosslinks and elute from beads. Supernatants were removed from beads, diluted with equal volumes of TE buffer, and treated with RNase A (1 μL, Ambion #2271), followed by Proteinase K (1 μL, Invitrogen). Alongside the ChIP samples, 3 μL of chromatin input extract (pre-ChIP) was added to elution buffer for the input samples and was reversed crosslinked, RNase and Proteinase K treated, and purified. Ampure bead purification was performed on the robot with a 1:1.8 DNA to Ampure bead ratio, and DNA was eluted in 20 μL elution buffer. DNA concentration was measured with the Quant-iT dsDNA high-sensitivity kit on the PHERAstar microplate reader and was subsequently diluted to a concentration of 1 ng/μL.

Illumina sequencing libraries were prepared from ChIP-enriched DNA or input DNA, using the ThruPLEX kit with 96 dual index adapters (Rubicon Genomics R400407) on a liquid-handling robot. Sequencing libraries were generally prepared from 10 ng (10 μL) of DNA; however, the amount of DNA ranged from 0.5 to 15 ng. Most libraries were amplified with 7 or 8 PCR cycles, but those with lower inputs of DNA into the library preparation were amplified with up to 16 PCR cycles. Libraries were run on an Agilent Tapestation 4200 with D1000 tapes for quantification. Libraries from each 96-well plate were mixed in equimolar concentrations into a single pool and sequenced on the Illumina HiSeq4000 with single end 50 base pair reads.

### Total RNA sequencing (RNA-seq)

Total RNA was extracted from approximately 25 mg of snap-frozen tissue per sample. Tissue was thawed into 700 μL TRIzol and homogenized using a Precellys 24 tissue homogenizer with cooling system and 2 mL grinding tubes with beads (soft-tissue kit CK14 for liver, brain and testis, or the hard-tissue grinding kit MK28-R for muscle) for two intervals of 30 s. RNA was purified with phenol:chloroform extraction followed by isopropanol precipitation. RNA concentration was measured on the NanoDrop, samples were diluted, and 1–10 μg of RNA was taken forward in the procedure. RNA was treated with the TURBO DNA-free kit (Invitrogen) to remove any residual DNA. Illumina sequencing libraries were prepared using the Illumina TruSeq Stranded Total RNA with Ribo Gold kit (20020598) with Illumina RNA UD Indexes (20020492) according to the manufacturer’s protocol. Samples were run on Agilent Tapestation D1000 tapes to quantify sequencing libraries. Up to 12 libraries were combined into a single pool and sequenced on the Illumina NovaSeq 6000 to generate paired-end 150 base pair reads.

### Genome resources

The genome versions used in this study can be found in Additional file 1: Table S1. All genomes were downloaded from the Ensembl version 98 [40] ftp as unmasked genomic DNA sequences, to facilitate the discovery of repetitive and transposable elements. For mouse, we used the primary assembly file, which excludes haplotypes and patches. All other species had no haplotype or patches, so we used the top-level DNA files.

### ChIP-seq mapping (Figures S1 and S2)

Reads were mapped to each species’ genome with BWA-MEM version 0.7.12 [62] using the default parameters, including the option to discard any alignment that has more than 10 thousand exact matches in the genome (−c 10,000). For all reads mapping to less than 10 thousand locations, the location with the highest mapping score was reported by BWA-MEM, or, in the case of multiple locations with the same score, a randomly selected location. Therefore, all reads with an exact repeat in 9999 other genomic locations would not have been used for downstream analyses, while reads with a smaller number of exact repeats might have been misplaced in the genome. Low-quality mapping reads were filtered out using SAMtools view version 1.3 with the -q1 flag [63]. Duplicates were removed with the Picard Tools MarkDuplicates program version 2.8.3 (https://broadinstitute.github.io/picard/). Mapping statistics were calculated using SAMtools flagstat version 1.3. To estimate the signal-to-noise ratio, we checked that the relative strand correlation (RSC) was above 0.8 for all libraries using Phantompeakqual tools version 1.14 [64]. The mapping and RSC results are available in Additional file 3: Table S3.

### ChIP-seq peak calling and signal saturation (Figures S1, S2A and B)

To ensure that we saturated the ChIP-seq signal for all libraries, we performed signal saturation tests (Additional file 1: Figure S2A). With SAMtools view version 1.3, we subsampled quality filtered and duplicate removed reads from each biological replicate starting from 5 million reads to the maximum library depth, or to a maximum of 60 million reads, with a step of 5 million. For each subsampled set, we called enriched ChIP-seq regions using MACS2 version 2.1.1 [65] using the broad peak mode (options: -q 0.05 --broad --broad-cutoff 0.1). An input library from the same individual and tissue (Additional file 3: Table S3) and subsampled to the same sequencing depth was also used with MACS2. To discover biologically reproducible peaks, we looked for ChIP-seq peaks within replicates that overlapped by 50% of their length with at least 50% of the peak of another replicate. Reproducible peaks appearing in at least two biological replicates were merged to produce the biologically reproducible set of histone enrichment peaks, while those not overlapping another replicate were not used for further analyses. The numbers of peaks per replicate and those peaks that are reproducible in at least two replicates are shown in Additional file 1: Figure S2B. Biologically reproducible H3K4me3 and H3K27ac reached ChIP-seq saturation at 20 million reads, while H3K4me1 reached saturation at 40 million reads (Additional file 1: Figure S2A).

We used the ChIP-seq libraries for H3K27ac and H3K4me3 subsampled to 20 million reads for all further analyses. Twelve of the somatic H3K4me3 libraries and one testis H3K4me3 library had less than 20 million reads after quality control and duplicate removal (Additional file 3: Table S3), so we used all the reads from these libraries instead of subsamples. This did not reduce the total H3K4me3 peak numbers because H3K4me3 saturates at a sequencing depth well below 20 million reads, especially in the somatic tissues (Additional file 1: Figure S2A). We subsampled all the H3K4me1 and matched input libraries to 40 million reads. The matched input sample for the macaque muscle library (unique identifier do17779) had around 21 million reads, which were used in MACS2 with the H3K4me1 library do17771.

### Capturing the signal across brain regions

To ensure that we are capturing the full complexity of the regulatory landscape in the brain, we compared our macaque H3K27ac ChIP-seq to a published study across three brain regions: cerebellum, cortex, and subcortical structures [49]. Across these three brain regions, Vermunt et al. identified a total of 61,795 H3K27ac peaks in the macaque genome version rheMac3 while we found 85,025 H3K27ac peaks in whole macaque brain using genome version Mmul_10, suggesting that we effectively captured the brain regulatory landscape.

### Definitions of regulatory regions (Fig. 1c and Table S4)

Within each species and tissue, we defined regulatory regions from the overlap of biologically reproducible ChIP-seq peak calling and signal saturation (Figures S1, S2A and B). H3K27ac enrichment is characteristic of active regulatory elements [52, 66, 67]. Concurrent H3K4me3 enrichment [42, 68–70] in active regulatory region is characteristic of promoters, while concurrent H3K4me1 enrichment [43, 52, 71] is characteristic of enhancers. We defined:

Active promoters as H3K4me3 enriched regions that overlapped a H3K27ac enriched region with at least 50% of their length, regardless of whether H3K4me1 enrichment is also present. We took the length of the H3K4me3 peaks as the final active promoter region, but excluded the entire joint length of H3K27ac and H3K4me3 from further regulatory region calls.

Active enhancers as H3K27ac histone enriched regions that overlap a H3K4me1 region with at least 50% of their lengths, keeping only the span of H3K27ac peaks as the final active enhancer region. We excluded the whole region marked with H3K27ac and H3K4me1 from further regulatory region calls.

Primed enhancers as H3K4me1 enriched regions that have no overlap with H3K27ac or H3K4me3 enriched regions.

### Reannotation of genomes (Fig. 1d)

For mouse and rat, we downloaded the available gene annotations from Ensembl version 98 [40]. For all other species (macaque, marmoset, rabbit, pig, cat, dog, horse, and opossum) we used a combination of our own RNA-seq data (Total RNA sequencing (RNA-seq)) and publicly available data to reannotate the genomes.

#### Transcript model generation

We generated gene annotations for each genome assembly using the previously described Ensembl annotation system [47]. Briefly, we generated transcript models from multiple evidence sources taken from the public archives, using a variety of approaches: (1) mapping publicly available short-read RNA-seq data from various tissues (search parameters: paired-end, ≥ 75 bp reads), including data generated by this study (ArrayExpress identifiers: E-MTAB-8122, E-MTAB-8118); (2) alignment of species-specific cDNAs (source: www.ebi.ac.uk/ena obtained March 2019) to the genome; and (3) protein-to-genome alignments of vertebrate UniProt (UniProt Consortium 2018) proteins with experimental evidence at protein and transcript levels. In addition, whole genome alignments against human GRCh38.p13 genome assembly were generated using LastZ [72] to identify regions of conserved synteny that subsequently guided mapping of conserved CDS exons from the GENCODE human gene set [73]. For pig and macaque, we mapped publicly available long-read transcriptome data (PRJNA351265 and PRJNA320013, respectively) to the genome using Minimap2 [74].

#### Transcript filtering and prioritization

For each locus, low-quality transcript models with suboptimal mapping, limited intron-defining short read support or non-canonical splice sites were removed before collapsing and clustering non-redundant transcripts into gene models. We prioritized models generated from transcriptome data, having strong intron supporting evidence and high sequence identity (> 90% coverage) to known vertebrate proteins. Gap filling was performed using homology data from projections to human annotations and mappings to UniProt proteins. To distinguish putative transcript isoforms from fragments, we assessed the coverage of protein alignments to each transcript relative to the size of the longest predicted open reading frame. Transcriptome data and cDNA alignments were used to extend models generated using homology data to annotate untranslated regions (UTR).

#### Gene model classification

We classified gene models into 3 main types: protein-coding, pseudogene, and long non-coding RNA (lncRNA) using alignment qualities of all supporting data for each model. Models with alignments to known proteins, having little or no overlaps with repeat regions of the genome, having high intron support and well-characterized canonical splice junctions were classified as protein-coding. Pseudogenes were annotated by identifying genes with alignments to known proteins but with evidence of frame-shifting or located in repeat regions of the genome. Single-exon models with a corresponding multi-exon copy elsewhere in the genome were classified as processed pseudogenes. Gene models generated using transcriptomic data (short and long reads), lacking any protein supporting evidence and did not overlap a protein-coding locus were classified as lncRNA.

Small non-coding RNA identification: Small non-coding (sncRNA) genes were added using annotations taken from RFAM [75] and miRbase [76]. BLAST [77] was run for these sequences to identify homologs in the genome sequence and models were evaluated for expected stem-loop structures using RNAfold [78]. Additional machine learning-based filters were applied to exclude predictions with sub-optimal alignments to the genome and non-conforming secondary structures. For other sncRNAs, models were built using the Infernal software suite [79].

### RNA-seq mapping and normalization (Fig. 1d and Table S5)

The RNA-seq reads were trimmed from adapters and for low-quality bases using Trimmomatic version 0.33 [80], using the included TrueSeq3 paired-end adapter sequences. To remove low-quality sequences from the reads, we removed those bases that had an average quality lower than 15 in a sliding window of four bases and the first and/or last three bases if below that threshold (options LEADING:3 TRAILING:3 SLIDINGWINDOW:4:15 MINLEN:36). We only kept reads with a minimum length of 36 bases, and only those that retained their paired read after trimming.

We mapped the trimmed RNA-seq reads using STAR version 2.6.0a [81], the Ensembl 98 version of the genomes (Genome resources, Additional file 1: Table S1), and gene annotation builds (Reannotation of genomes (Fig. 1d)) to map each replicate RNA-seq library to known genes and transcripts. For STAR mapping, we used the following options:

--outFilterType BySJout --outFilterMultimapNmax 100 --winAnchorMultimapNmax 100 --alignSJoverhangMin 8 --alignSJDBoverhangMin 1 --outFilterMismatchNmax 999 --outFilterMismatchNoverReadLmax 0.04 --alignIntronMin 20 --alignIntronMax 1000000 --quantMode GeneCounts --outSAMtype BAM SortedByCoordinate --outSAMstrandField intronMotif.

To normalize the RNA-seq mapped libraries across replicates and tissues of the same species, we used Cufflinks version 2.2.1 [82]. We used the Cuffquant command specifying the strandedness of the library (option --library-type=fr-firststrand), followed by the Cuffnorm program treating each tissue as a sample, and each biological replicate as a replicate for that tissue. This produced normalized expression values for each annotated gene and transcript within a species and across all tissues. In Fig. 1d, a gene or transcript was considered expressed in a tissue if this normalized value was above 0 FPKM.

### Enrichment of called regulatory regions (Figures S2C, S4 and S8)

To investigate the enrichment of peak calls underlying regulatory region calls, we compared their q-values as reported by MACS2 (ChIP-seq peak calling and signal saturation (Figures S1, S2A and B)). Specifically, for each regulatory region, we computed the average *q* value across all replicates’ peaks for each histone mark separately using the average function in bedtools merge -o mean function. To create a set that is comparable between regulatory regions we selected for comparisons on the ChIP marks which the regions share. For active promoters and active enhancers, we used H3K27ac—this selected for the strongest active enhancers and weakest active promoters on the mark they share. Similarly, to make the active and primed enhancers comparable, we used H3K4me1, which selected for the strongest primed enhancers and weakest active enhancers (Additional file 1: Figure S2C).

To compare the signal-to-input enrichment within peaks, we also used the fold-change as reported by MACS2 (ChIP-seq peak calling and signal saturation (Figures S1, S2A and B). For Additional file 1: Figure S4, we report all the fold-changes of all replicate peaks called after normalization of libraries to depth reported before and using the same *q* value cutoff (ChIP-seq peak calling and signal saturation (Figures S1, S2A and B)).

### Validation of called regulatory regions (Figures S3A, B and C)

Mouse regulatory regions identified in the current study were compared to mouse regulatory regions annotated in Ensembl version 98 [83] and NCBI RefSeq functional elements [84] (downloaded September 26, 2017). We asked how many of the active promoters identified in the current study were annotated as promoters in either the Ensembl or RefSeq database. Given that neither external databases differentiate between enhancer types (i.e., active and primed enhancers) in an analogous manner to us, we combined primed and active enhancers identified in the current study into a single set. We then overlapped these enhancers with enhancers identified in either the Ensembl or RefSeq database. Overlap of any length in the genomic coordinates between a regulatory region identified in the current study and one annotated in the other database (Ensembl or RefSeq) was interpreted to mean the regulatory regions were common between the two sets, and lack of overlap was interpreted as a regulatory region specific to either the current study or to the other database (Ensembl or RefSeq). For simplicity, only regulatory regions mapping to chromosomes were considered for this analysis (those mapping to scaffolds were not considered). The resulting analyses are shown in Additional file 1: Figure S3A.

For histone enrichment plots, local installations of deepTools version 3.3.1 [85] and WiggleTools [86] were used as follows: deepTools bamCompare was first used to subtract the corresponding input libraries from all quality controlled and duplicate removed (but not subsampled) ChIP-seq libraries and then WiggleTools mean to calculate the ChIP-seq enrichment within each mouse tissue as an average across all biological replicates for each histone mark. To create the heatmaps in Additional file 1: Figure S3C, the resulting averages of read enrichment from H3K4me3, H3K27ac, and H3K4me1 ChIP-seq libraries were compared with Ensembl Validated (i.e., overlap of our regions and Ensembl regulatory regions) and our novel regulatory mouse regions using the deepTools computeMatrix program with the options scale-regions --beforeRegionStartLength 2000 --afterRegionStartLength 2000 --missingDataAsZero --regionBodyLength 2000 --skipZeros and then plotted with the deepTools plotHeatmap program.

### Generating genome browser tracks (Figs. 1a and b)

A biological replicate from a single individual for each species and tissue was arbitrarily chosen to display in the genome browser. Files were visualized in the IGV genome browser [87] with the appropriate genome and gene annotations files for each species. The genomic region around the genes encoding myosin heavy chains 1 and 2 (*Myh1* and *Myh2*) were extracted for each species and tissue from either bedGraph files (for ChIP-seq data), which represent read pileups for that biological replicate as generated by MACS2 (ChIP-seq peak calling and signal saturation (Figures S1, S2A and B)) or wig files of uniquely mapping reads from the STAR alignments for the RNA-seq data (RNA-seq mapping and normalization (Fig. 1d and Table S5)). Bedgraph files were converted to the TDF file format with IGV tools to aid visualization in the browser. RNA-seq data are stranded; however, the signal from the coding strand greatly dominated over the non-coding strand, and therefore, only the coding strand was shown. Muscle samples visualized in Fig. 1a were do17377 (macaque H3K4me3), do17393 (macaque H3K27ac), do18035 (macaque H3K4me1), do22674 (macaque RNA), do17664 (marmoset H3K4me3), do17690 (marmoset H3K27ac), do17715 (marmoset H3K4me1), do22678 (marmoset RNA), do15511 (mouse H3K4me3), do15539 (mouse H3K27ac), do15528 (mouse H3K4me1), do22610 (mouse RNA), do15941 (rat H3K4me3), do15952 (rat H3K27ac), do15918 (rat H3K4me1), do22601 (rat RNA), do17178 (rabbit H3K4me3), do17199 (rabbit H3K27ac), do17112 (rabbit H3K4me1), do22688 (rabbit RNA), do17356 (cat H3K4me3), do17365 (cat H3K27ac), do18036 (cat H3K4me1), do22638 (cat RNA), do17647 (dog H3K4me3), do17694 (dog H3K27ac), do17725 (dog H3K4me1), do22643 (dog RNA), do15887 (horse H3K4me3), do15926 (horse H3K27ac), do15954 (horse H3K4me1), do22676 (horse RNA), do17342 (pig H3K4me3), do16006 (pig H3K27ac), do16028 (pig H3K4me1), do26160 (pig RNA), do14518 (opossum H3K4me3), do14483 (opossum H3K27ac), do14565 (opossum H3K4me1), and do22663 (opossum RNA) (Additional file 3: Table S3 and Additional file 4: Table S5). For mouse and dog, the same muscle samples from the same individuals were visualized in Fig. 1b. Brain, liver and testis samples visualized in Fig. 1b were do17085 (mouse brain H3K4me3), do17013 (mouse brain H3K27ac), do17044 (mouse brain H3K4me1), do22662 (mouse brain RNA), do15990 (mouse liver H3K4me3), do16031 (mouse liver H3K27ac), do16016 (mouse liver H3K4me1), do26179 (mouse liver RNA), do17048 (mouse testis H3K4me3), do17010 (mouse testis H3K27ac), do17079 (mouse testis H3K4me1), do22603 (mouse testis RNA), do17046 (dog brain H3K4me3), do17056 (dog brain H3K27ac), do17100 (dog brain H3K4me1), do22652 (dog brain RNA), do17397 (dog liver H3K4me3), do17324 (dog liver H3K27ac), do17341 (dog liver H3K4me1), do22650 (dog liver RNA), do17392 (dog testis H3K4me3), do17345 (dog testis H3K27ac), do17327 (dog testis H3K4me1), and do26151 (dog testis RNA).

### Intra-species cross-tissue activity (Fig. 2a, Figures S5 and S6A)

Within each species, we defined the tissue-specificity of regulatory regions by comparing the regulatory calls made within each of the tissues separately (Definitions of regulatory regions (Fig. 1c and Table S4)). Two regulatory regions were considered active across tissues if either overlapped another regulatory region of the same regulatory activity with at least 50% of its length. i.e., a tissue-shared active enhancer was considered tissue-shared only if it overlapped an active enhancer in another tissue (Additional file 1: Figure S5). All other combinations were considered intra-species dynamic (Intra-species dynamic regulatory signatures (Fig. 4a and S5)) and not included in any analyses or figures unless explicitly stated. A gene or transcript was considered expressed in a tissue according to the method outlined in RNA-seq mapping and normalization (Fig. 1d and Table S5).

Figure 2b shows the sum across all ten species for the intersections between tissue activity using an UpSetR plot version 1.4.0 [88], while Additional file 1: Figure S6A shows the data only for mouse. For all further analyses regulatory regions and gene expression were considered tissue-specific if they were only active in a single tissue and tissue-shared if active in two or more tissues (Additional file 1: Figure S5).

### Association of enhancers to promoters (Fig. 2b and Figure S6B)

We used a distance rule to associate enhancers to promoters they might regulate, given that around 70% of enhancers do act on their nearest gene [53, 54]. Within each tissue, we associated primed and active enhancers called in that tissue to the nearest active promoter also called in that tissue. If there was no active promoter within 1 Mb of the enhancer, they were not assigned to an active promoter. We next tagged each active promoter, active and primed enhancer as tissue-specific or tissue-shared using the same rules as above (Intra-species cross-tissue activity (Fig. 2a, Figures S5 and S6A)) and defined an extra category for promoters—those promoters active across all four tissues were defined as 4-tissue-shared. In Fig. 2b, we show the distribution of the numbers of active and primed enhancers associated to tissue-specific and tissue-shared active promoters in each tissue. Promoters with more than 20 associated enhancers of any type are excluded from the graph, and a regression line representing the ratio of tissue-specific to tissue-shared enhancers is shown. The data is a summary across all ten study species.

In Additional file 1: Figure S6B, we show the same data without excluding promoters with more than 20 associated enhancers. In this graph, we represent the number of tissue-shared and tissue-specific enhancers as log-transformed ratios, adding a pseudocount to avoid dividing by zero.

### Associations of regulatory regions to genes and differential gene expression analysis (Fig. 2c)

For each active promoter within each tissue, we found the closest TSS in the given species using the Ensembl gene annotation created for this project (Reannotation of genomes (Fig. 1d)). The TSS was defined as the start, i.e., most downstream coordinate, of a gene and that gene associated to a promoter if not further away than 1 Mb. Next, we used the enhancer-promoter association from above (Association of enhancers to promoters (Fig. 2b and Figure S6B)) to extend each active promoter-associated gene to apply to that promoters’ enhancers.

We performed differential gene expression analyses between all other tissues and liver in a pairwise manner using DESeq2 version 1.10.1 [89] with default parameters and a Benjamini-Hochberg adjusted *p* value threshold of 0.05 (*p*adj < 0.050000). As input for DESeq2, we used raw read counts produced with the STAR aligner (RNA-seq mapping and normalization (Fig. 1d and Table S5)). Specifically, for each species, we tested the differential expression between the muscle, brain, or testis against the liver and in Fig. 2c report log2fold changes that passed the thresholds. Within each tissue, we further created a more stringent subset from all tissue-shared regulatory regions (Intra-species cross-tissue activity (Fig. 2a, Figures S5 and S6A)) to only include those shared across all four tissues (4-tissue-shared).

### Whole genome alignments

For whole genome alignment between the eutherian mammals (macaque, marmoset, mouse, rat, rabbit, pig, cat, dog, and horse), we used the EPO eutherian mammal alignments from Ensembl version 98 [90]. For whole genome alignments between the eutherian mammals and opossum, we used the PECAN alignments also from Ensembl version 98. We aligned all species to mouse in a pairwise manner, first from all other species to mouse and then from mouse to all other species. A regulatory region was considered maintained (*P*_*Mi*_; see Eq. 1 and Additional file 1: Figure S5) if it overlapped another regulatory region of any type with at least one base. A regulatory region was considered recently evolved (*P*_*Ri*_; see Eq. 2 and Additional file 1: Figure S5) if it was aligned to other species but did not overlap a regulatory region in any of them, or if it was not aligned to any other species. Any regulatory region aligned to multiple locations was excluded from further analyses, i.e., only 1-to-1 alignments were kept.

Equations demonstrating the computation of cross-species conservation of promoters are shown in the following sections. The same operations were computed for active and primed enhancers but are not shown here.

### The recently evolved and maintained regulomes across species (Fig. 3a)

1 $$ {P}_{Ai}={P}_{Ni}+{P}_{Li}+{P}_{Mi} $$

1.1 $$ {P}_{Mi}={P}_{Ai}-{P}_{Ni}-{P}_{Li} $$

*P*_*Ai*_—number of all active promoters in species *i*

*P*_*Ni*_—number of active promoters in species *i* with no alignment to any other species

*P*_*Li*_—number of active promoters in species *i* with an alignment to any other species, but no regulatory region aligned in any other species

*P*_*Mi*_—number of active promoters in species *i* with an alignment of at least one base length to any regulatory region in any other species

2 $$ {P}_{Ri}={P}_{Ni}+{P}_{Li} $$

*P*_*Ri*_—number of recently evolved active promoters in species *i*

Figure 3a shows the ∑***P***_***Mi***_ (Eq. 1.1) and ∑***P***_***Ri***_ (Eq. 2) across all ten species for active promoters, and analogous calculation for active and primed enhancers

### Pairwise comparisons between species (Figs. 3b and c)

We performed two pairwise comparisons, the first stratifying regulatory regions by tissue-shared and tissue-specific (Fig. 3b), and the second further stratifying tissue-specific regulatory regions by their tissue of activity (i.e., liver-specific, muscle-specific, brain-specific, and testis-specific) (Fig. 3c). The stratification was limited to the identity of the query regulatory region, but the query region was considered maintained if it aligned to any regulatory region in the other species (regardless of tissue-specificity). For example, a liver-specific mouse active promoter was consider maintained and counted as tissue-specific (Fig. 3b) and liver-specific (Fig. 3c) in all the following cases: (1) if it aligned to a liver-specific active promoter, (2) if it aligned to a tissue-shared active promoter, (3) if it aligned to a muscle-specific active promoter, or (4) if it aligned to active or primed enhancers of any tissue-specificity in the other species. For definitions of tissue-specificity, see the “Materials and methods” section Intra-species cross-tissue activity (Fig. 2a, Figures S5 and S6A).

3 $$ {P}_{Mi,j}=\left(\frac{P_{Mi\to j}}{P_{Mi\to j}+{P}_{Li\to j}}+\frac{P_{Mj\to i}}{P_{Mj\to i}+{P}_{Lj\to i}}\right)\times 100 $$

*P*_*Mi*, *j*_—fraction of alignable active promoters with activity in both species *i* and *j*, i.e. aligned to a regulatory active region, defined as maintained regulatory regions. See also Eq. 6

*P*_*Mi* → *j*_—number of active promoters in species *i* with an alignment of at least one base length to any regulatory region in species *j*

*P*_*Li* → *j*_—number of active promoters in species *i* with an alignment of at least one base length to species *j,* but not aligned to a regulatory region

To calculate the zero point, we generated a fourth ChIP-seq replicate for all histone modifications for mouse and cat (Additional file 2: Table S3) and called peaks using the same methods as for other replicates (ChIP-seq peak calling and signal saturation (Figures S1, S2A and S2B)). We then used all possible combinations of three replicates to estimate interindividual reproducibility (Definitions of regulatory regions (Fig. 1c and Table S4)) as a measure of both the variation between individuals and biases introduced by our analyses.

4 $$ {P}_{Ik,l}=\left(\frac{P_{Ik\to l}}{P_{Ak}} + \frac{P_{Il\to k}}{P_{Al}\ }\right)\times 100 $$

*P*_*Ik*, *l*_—fraction of active promoters with regulatory activity between a pair of biological replicates *k* and *l*, i.e. reproducible between individuals

*P*_*Ik* → *l*_ —number of active promoters in individual *k* that overlap a regulatory active region in individual*l*by at least one base

*P*_*Ak*_ —total number of active promoters in individual *k*

Figure 3b and c show *P*_*Mi*, *j*_ (Eq. 3) for every pair of species at divergence > 0 MYA (45 comparisons) and *P*_*Ik*, *l*_ (Eq. 4) at divergence = 0 for every pair of 4 mouse and every pair of 4 cat biological replicates (12 comparisons). For Fig. 3b, we first plotted regions identified as tissue-shared in species *i* and species *j*, and then regions identified as tissue-specific in species *i* and species *j.* Similarly, for Fig. 3c, we plotted separately all tissue-specific regions depending on which tissue they were active in. We plotted the resulting graphs in R version 3.6.2 [91] using ggplot2 version 3.1.1 [92] and performed linear regression using the geom_smooth() ggplot2 method. To test for statistical significance, we fitted the data to a linear model using the R function lm() and tested the resulting linear models using the built-in anova() function for the interaction of divergence time and tissue-specificity. Specifically, for Fig. 3b, we report the two-way ANOVA *p* value for the interaction of divergence time (factor 1) and regions identified as active promoters and active enhancers (factor 2), for the interaction of divergence time (factor 1) and regions identified as active promoters and primed enhancers (factor 2), and for the interaction of divergence time (factor 1) and regions identified as active enhancers and primed enhancers (factor 2). For Fig. 3c, we first report the two-way ANOVA *p* value for the interaction of divergence time (factor 1) and active promoters identified as testis-specific or any other tissue-specific region (factor 2). We then report the two-way ANOVA *p* value for the interaction of divergence time (factor 1) and active promoters being identified as tissue-specific or tissue-shared (factor 2). All divergence times between species were taken from Ensembl Compara version 98 [90].

### Intra-species dynamic regulatory signatures (Fig. 4a and Figure S5)

To define regulatory regions that change regulatory identity between the tissues of a species, we performed cross-tissue overlap as described for determining tissue-specific and tissue-shared regions (Intra-species cross-tissue activity (Fig. 2a, Figures S5 and S6A)). Briefly, if regulatory regions of different identities overlapped each other with at least 50% of their length between tissues, we called these regions intra-species dynamic (Additional file 1: Figure S5). Specifically, overlap of an active promoter in one tissue to either an active or primed enhancer in another tissue was called a dynamic promoter/enhancer (dynamic P/E), while the overlap of an active enhancer in one tissue to a primed enhancer in another tissue was called a dynamic enhancer (dynamic E). The sum of all dynamic regions, and non-dynamic regions, across all ten species is shown in Fig. 4a.

### Evolutionary dynamic regulatory signatures (Fig. 4b and Figure S5)

For all maintained regulatory regions (*P*_*Mi*, *j*_ Eq. 3, The recently evolved and maintained regulomes across species (Fig. 3a), Additional file 1: Figure S5), we next asked how the regulatory signature changes through evolution. For each pairwise comparison between species, we counted how many regulatory regions of one identity aligned with at least one base to a regulatory region of all other signatures. These calculations were limited only to maintained regulatory regions which only align to one other regulatory region between species. The text below shows the calculation for active promoters as an example, but all other regulatory regions were calculated similarly.

5 $$ {NP}_{Mi\to j}={P}_{Pi\to j}+{P}_{AEi\to j}+{P}_{PEi\to j}+{P}_{DPi\to j}+{P}_{DEi\to j} $$

*NP*_*Mi* → *j*_—total number of maintained promoters in species *i* when compared to species *j*

*P*_*Pi* → *j*_—Total number of active promoters in species *i* with a 1-to-1 alignment to at least one base of an active promoter in species *j;* these represent evolutionarily stable promoter signatures

*P*_*AEi* → *j*_—Total number of active promoters in species *i* with a 1-to-1 alignment to at least one base of an active enhancer in species *j;* these represent evolutionarily dynamic promoter signatures

*P*_*PEi* → *j*_—Total number of active promoters in species *i* with a 1-to-1 alignment to at least one base of a primed enhancer in species *j;* these represent evolutionarily dynamic promoter signatures

*P*_*DPi* → *j*_—Total number of active promoters in species *i* with a 1-to-1 alignment to at least one base of an intra-species dynamic promoter region in species *j;* see also the Intra-species dynamic regulatory signatures (Figs. 4a and S5) section

*P*_*DEi* → *j*_—Total number of active promoters in species *i* with a 1-to-1 alignment to at least one base of an intra-species dynamic enhancer region in species *j;* see also the Intra-species dynamic regulatory signatures (Figs. 4a and S5) section

Figure 4b shows the ∑(*P*_*Pi* → *j*_ + *P*_*AEi* → *j*_ + *P*_*PEi* → *j*_ + *P*_*DPi* → *j*_ + *P*_*DEi* → *j*_) for all pairs of species for the active promoters, and analogous calculations for the other regulatory regions, in a Circos plot [93]

### Evolutionary dynamic regulatory identities across divergence time (Fig. 4c)

Next, we asked if evolutionary dynamics of promoter and enhancer signatures is correlated to the divergence time between species. For this, we focused on all maintained regulatory regions (The recently evolved and maintained regulomes across species (Fig. 3a)) between pairs of species and asked how often they align to a regulatory region with another regulatory signature (Evolutionary dynamic regulatory signatures (Fig. 4b and Figure S5)). The comparisons were limited to 1-to-1 aligned regulatory regions.

6 $$ {P}_{Wi,j}=\left(\frac{P_{Wi\to j}}{P_{Mi\to j}} + \frac{P_{Wj\to i}}{P_{Mj\to i}\ }\right)\times 100 $$

*P*_*Wi*, *j*_—fraction of maintained active promoters switching regulatory activity between species *i* and *j*, i.e., aligned to a regulatory active region, defined as evolutionarily dynamic promoter signatures. See also Eq. 5

*P*_*Wi* → *j*_—number of active promoters in species *i* aligned to an active or primed enhancer in species *j*

*P*_*Mi* → *j*_—number of active promoters in species *i* aligned to any regulatory region in species *j;* see also Eq. 3

*P*_*Wj* → *i*_—number of active promoters in species *j* aligned to an active or primed enhancer in species *i*

*P*_*Mj* → *i*_—number of active promoters in species *j* aligned to any regulatory region in species *i;* see also Eq. 3

6.1 $$ {AE}_{Wi,j}=\left(\frac{AE_{Wi\to j}}{AE_{Mi\to j}\ }\right)\times 100 $$

*AAE*_*Wi*, *j*_—fraction of maintained active enhancers switching regulatory activity between species *i* and *j*, i.e., aligned to a regulatory active region*,* defined as evolutionarily dynamic enhancer signatures. See also Eq. 5

*AE*_*Wi* → *j*_—number of active enhancers in species *i* aligned to a primed enhancer in species *j*

*AE*_*Mi* → *j*_—number of active enhancers in species *i* aligned to any regulatory region in species *j;* see also Eq. 3

Figure 4c shows the *P*_*W*, *j*_ and *AE*_*Wi*, *j*_ between all pairs of species (45 comparisons) for every pair of species at divergence > 0 MYA (45 comparisons) and at divergence = 0 the average intra-species dynamic activity (Intra-species dynamic regulatory signatures (Figs. 4a and S5)). All divergence times between species were taken from Ensembl version 98 [90]. We plotted the resulting graphs in R version 3.6.2 [91] using ggplot2 version 3.1.1 [92] and performed linear regression using the geom_smooth() ggplot2 method

### Outgroup analysis (Fig. 4d, Figures S8B and S8C)

Whole genome alignments of regulatory regions were parsed to get all 1-to-1 alignments for mouse/rat/rabbit and separately for cat/dog/horse (see the Evolutionary dynamic regulatory signatures (Fig. 4b and Figure S5) section). Only genomic regions that were maintained as either an active promoter, active enhancer, or primed enhancer in all three of the species in the triad (mouse/rat/rabbit and cat/dog/horse) were considered in this analysis. Genomic regions identified as an intra-species dynamic region in any of the three species were excluded from this analysis for simplicity. Analyses were done separately for each triad. The overall proportion of active promoters, active enhancers, and primed enhancers in the outgroup species (rabbit or horse) for the genomic regions considered is shown as “All” in Fig. 4d and Additional file 1: Figure S8. Given the combination of regulatory signatures in the ingroup species (mouse/rat or cat/dog), we asked what the identity was in the outgroup species (rabbit or horse, respectively). For example, in the AP/AE situation, this could represent either an active promoter in mouse and an active enhancer, or vice versa. The regulatory signature of the genomic region in rabbit would then be queried. Percentages and raw numbers are shown separately for each triad in Additional file 1: Figure S8B and Additional file 1: Figure S8C. The combined numbers and percentages are also shown in the bottom panels of Additional file 1: Figure S8B and C and in Fig. 4d. Chi-square two-tailed tests (degrees of freedom = 2) were used to test whether outgroup distributions of active promoters, active enhancers, and primed enhancers for each ingroup combination differed statistically from the background (“All”) distribution (Fig. 4d). Expected values were calculated based on the percentages of active promoters, active enhancers, and primed enhancers in the background.

### Model of evolutionary dynamics between regulatory regions (Fig. 4e)

For the model of all possible evolutionary dynamics between active promoters (*AP*), active enhancers (*AE*) and primed enhancers (*PE*), we extracted probabilities using the observed frequencies in the triad analysis above (Outgroup analysis (Fig. 4d, Figures S8B and C)). For each regulatory region, we calculated the relative probabilities of retaining the same signature (*AP* → *AP*, *AE* → *AE*, and *PE* → *PE*), and changing to a regulatory region of another signature (for example, *AP* → *AE* or *AE* → *AP*). The probabilities were calculated from the observed frequencies in both outgroup analyses combined (mouse/rat/rabbit and cat/dog/horse), using only those evolutionary relationships where parsimony could be used to determine the ancestral state as a single regulatory region signature.

Specifically, for active promoters:

7 $$ \frac{P\left( AP\to AP\right)+P\left( AP\to AE\right)+P\left( AP\to PE\right)}{P_{\mathrm{total}}}=1 $$

7.1 $$ {P}_{\mathrm{total}}=\sum P\left( AP\to AP\right)+P\left( AP\to AE\right)+P\left( AP\to PE\right) $$

*P*(*AP* → *AP*)—probability of an ancestral active promoter remaining an active promoter. Observed frequency from triad relationship ingroups AP/AP and outgroup AP

*P*(*AP* → *AE*)—probability of an ancestral active promoter evolving to an active enhancer. Observed frequency from triad relationship ingroups AP/AE and outgroup AP

*P*(*AP* → *PE*)—probability of an ancestral active promoter evolving to a primed enhancer. Observed frequency from triad relationship ingroups AP/PE and outgroup AP

Specifically, for active enhancers:

8 $$ \frac{P\left( AE\to AE\right)+P\left( AE\to AP\right)+P\left( AE\to PE\right)}{AE_{\mathrm{total}}}=1 $$

8.1 $$ {AE}_{\mathrm{total}}=\sum P\left( AE\to AE\right)+P\left( AE\to AP\right)+P\left( AE\to PE\right) $$

*P*(*AE* → *AE*)—probability of an ancestral active enhancer remaining an active enhancer. Observed frequency from triad relationship ingroups AE/AE and outgroup AE

*P*(*AE* → *AP*)—probability of an ancestral active enhancer evolving to an active promoter. Observed frequency from triad relationship ingroups AE/AP and outgroup AE

*P*(*AE* → *PE*)—probability of an ancestral active enhancer evolving to a primed enhancer. Observed frequency from triad relationship ingroups AE/PE and outgroup AE

Specifically, for primed enhancers:

9 $$ \frac{P\left( PE\to PE\right)+P\left( PE\to AP\right)+P\left( PE\to AE\right)}{PE_{\mathrm{total}}}=1 $$

9.1 $$ {PE}_{\mathrm{total}}=\sum P\left( PE\to PE\right)+P\left( PE\to AP\right)+P\left( PE\to AE\right) $$

*P*(*PE* → *PE*)—probability of an ancestral primed enhancer remaining a primed enhancer. Observed frequency from triad relationship ingroups PE/PE and outgroup PE

*P*(*PE* → *AP*)—probability of an ancestral primed enhancer evolving to an active promoter. Observed frequency from triad relationship ingroups PE/AP and outgroup PE

*P*(*PE* → *AE*)—probability of an ancestral primed enhancer evolving to an active enhancer. Observed frequency from triad relationship ingroups PE/AE and outgroup PE

Figure 4e shows the resulting calculations for all evolutionary relationships as numbers above the arrows.

### ChIP-seq and RNA-seq read enrichment around evolutionarily dynamic regulatory regions (Figs. 4f and g)

To validate evolutionary switching from active enhancer to active promoter (evolutionary dynamic P/Es, Additional file 1: Figure S5), we selected a subset of regulatory regions from the outgroup analysis (Outgroup analysis (Fig. 4d, Figures S8B and S8C)) that are most likely to represent a true evolutionary switch. Specifically, we only chose those regions that had an active promoter signature in one ingroup, an active enhancer signature in the other ingroup, and an active enhancer signature in the outgroup as the most parsimonious conclusion is that the ancestral state was an active enhancer.

For Fig. 4f, we separated these regions within each outgroup species into sets that showed active promoter signature (“AP Dynamic”) and active enhancer signature (“AE Dynamic”). We then selected from the same species the same number of control regions as those that never show evolutionarily dynamic activity. We next generated the per-species averages of ChIP-seq enrichment across these regions for all replicates of a species as described before (Enrichment of called regulatory regions (Figures S2C, S4 and S8)) but extending the flanking regions to 10 Kb. Finally, we averaged across all per-species averages to generate the graphs in Fig. 4f.

For Fig. 4g, we used the same AP Dynamic and AE Dynamic sets as described above, but only the active enhancers as control regions. For RNA-seq enrichment plots, we used local installations of deepTools version 3.3.1 [85] and WiggleTools mean [86] to calculate the maximum RNA-seq enrichment across all biological replicates across all tissues in a species. For Fig. 4g, the resulting maximum RNA-seq values were compared between evolutionarily dynamic and control regions using the deepTools computeMatrix program with the options scale-regions --beforeRegionStartLength 10000 --afterRegionStartLength 10000 --missingDataAsZero --regionBodyLength 2000 –skipZeros within each species. To generate the resulting boxplots in Fig. 4g, all species’ values were combined. The *p* values were calculated with the ggpubr package in R [94], using the stat_compare_means function using a *t* test testing if active promoters (AP) had higher expression than active enhancers (AE) than control regions.

### Tissue-specificity of evolutionarily dynamic regions (Fig. 4h)

To create the tissue-specificity UpSetR version 1.4.0 plots [88] in Fig. 4h, we extracted all regions corresponding to evolutionary dynamic promoter signatures (Additional file 1: Figure S5, Evolutionary dynamic regulatory signatures (Fig. 4b and Figure S5)). Specifically, we extracted those active promoters that have a 1-to-1 alignment to an active or primed enhancer in any other species and active and primed enhancers that have a 1-to-1 alignment to an active promoter in another species. We then considered the tissue-specificity of the regions categorizing them according to the regulatory identity in the species they were extracted from. For example, for a region identified as an active promoter in rat and an active enhancer in rabbit, we considered its tissue-specificity only in rat for the active promoter category and tissue-specificity only in rabbit for the active enhancer category. We plotted the within species tissues specificity of those selected regulatory regions as outlined in the “Materials and methods” section Intra-species cross-tissue activity (Fig. 2a, Figures S5 and S6A). We performed all statistical tests using the binom.test() function in R version 3.6.2 [91].

### Repeat masking and classification of transposable elements

To identify transposable elements in all genomes, we used RepeatMasker version open-4.0.7 [95] using the crossmatch search engine and RepBase Release 20,170,127 [96]. For each species we ran RepeatMasker in the default mode, specifying the species’ scientific name (macaque, “*Macaca mulatta*”; narmoset, “*Callithrix jacchus*”; mouse, “*Mus musculus*”; rat, “*Rattus norvegicus*”; rabbit, “*Oryctolagus cuniculus*”; pig, “*Sus scrofa*”; dog, “*Canis familiaris*”; cat, “*Felis catus*”; horse, “*Equus caballus*”; opossum, “*Monodelphis domestica*”). For figures, we used DNA, LINE, LTR, and SINE categories from RepBase annotation. These correspond to different levels of transposable element classification hierarchies [23], but still represent exclusive non-overlapping sets of the hierarchy. Namely, DNA corresponds to the DNA transposons class, while all other groups belong to the retrotransposon classes. The LTRs are a subclass of retrotransposons, while the LINEs and SINEs are superfamilies of the non-LTR subclass of retrotransposons.

### Relative transposable element enrichment of tissue-specific and tissue-shared recently evolved regulatory and maintained regions (Fig. 5a and Figure S9B, Tables S6 and S7)

We first extracted all recently evolved and maintained regulatory regions (Additional file 1: Figure S5, The recently evolved and maintained regulomes across species (Fig. 3a)). Next, within each species, we calculated the number of tissue-specific recently evolved regions that overlap half the length of transposable elements within subgroups as defined by RepBase (Repeat masking and classification of transposable elements). For example, for tissue-specific active promoters overlapping any LINE with at least one base, we counted the number occurring in all possible subgroups (for example, L1, L2, and CR1). We next repeated the same process for tissue-shared active promoters overlapping any LINE. To generate the relative enrichment shown in Fig. 5a and Additional file 1: Figure S9B, we calculated the percent of tissue-specific and tissue-shared regulatory regions overlapping specific subgroups by dividing each subgroup count by the total counts for that group and multiplying by 100. For example, for L1, we divided the number of tissue-specific active promoters overlapping an L1 by the total number of tissue-specific active promoters overlapping any LINE. Similarly, for the tissue-shared, we divided the number of tissue-shared active promoters overlapping an L1 by the total number of tissue-shared active promoters overlapping any LINE. Finally, we subtracted the tissue-shared portions with the tissue-specific to generate a relative enrichment. Consequently, positive values indicate a higher proportion of that subgroup in the tissue-specific than in the tissue-shared. For example, pig has a value of 16 for L1 active promoters because 47% of all tissue-specific active promoters overlapping a LINE belonged to the L1 subgroup, compared to 31% of the tissue-shared active promoters. The heatmap of all relative enrichments in Fig. 5a and Additional file 1: Figure S9B were generated using the heatmap.2() function in gplots package version 3.0.1.1 [97]. The *p* values were calculated on the original counts, using the *Z*-test in R base function prop.test and Bonferonni correction using the total number of tests across the matrix implemented in R base function p.adjust. For generating the heatmaps images, we filtered all possible subgroups to include only those that have at least 100 tissue-specific and 100 tissue-shared occurrences in any species and manually refined the selection to only include those that are informative for multiple lineages, but total counts across all subgroups were used for the *p* value calculations.

Figure 5a shows the relative transposable element enrichments for recently evolved regulatory regions, while Additional file 1: Figure S9B shows the enrichments for maintained regulatory regions.

### LINEs in random genomic regions (Figure S9)

To compare LINE overlap of regulatory regions to random regions, within each species, we randomly selected the same number and length of random regions as there were regulatory regions using bedtools shuffle and excluding those regions that we found to be regulatorily active. We then overlapped these random regions with LINEs, requiring 50% overlap with LINEs, and compared the portion of random and regulatory regions that overlapped a LINE.

### ChIP-seq and RNA-seq read enrichment around LINE-associated recently evolved regulatory regions (Fig. 5b and c)

To examine the raw signal surrounding LINE-associated active promoters, we first extracted recently evolved active promoters overlapping a LINE L1 or LINE L2 with at least one base (overlap defined in the “Relative transposable element enrichment of tissue-specific and tissue-shared recently evolved regulatory and maintained regions (Fig. 5a and Figure S9B, Tables S6 and S7)” section). We next chose those active promoters that had overlap with LINE L2s and have tissue-shared activity (as defined in the Intra-species cross-tissue activity (Fig. 2a, Figures S5 and S6A) section). For those active promoters that had overlap with LINE L1s and were tissue-specific, we further subdivided them by the tissue of activity. For Fig. 5b, we generated the per-tissue averages of ChIP-seq enrichment across all replicates of a species as described before (Enrichment of called regulatory regions (Figures S2C, S4 and S8)) but making an average across all replicates of a tissue within a species and extending the flanking regions to 10 Kb. Finally, we combined averaged across all per-species averages to generate the graphs in Fig. 5b.

For RNA-seq enrichment plots, we used local installations of deepTools version 3.3.1 [85] and WiggleTools max [86] to calculate the maximum RNA-seq enrichment across all biological replicates of the tissue. For Fig. 5c, the resulting maximums RNA-seq values were compared to LINE associated active promoters using the deepTools computeMatrix program with the options scale-regions --beforeRegionStartLength 10000 --afterRegionStartLength 10000 --missingDataAsZero --regionBodyLength 2000 –skipZeros within each species. To generate the resulting boxplots in Fig. 5c, all species’ values were combined. The *p* values were calculated with the ggpubr package in R [94], using the stat_compare_means function using a *t*-test testing if the mean of each boxplot is significantly different from all other expressed in that tissue (i.e., within rows).

### Age of LINEs (Fig. 5d and Figure S10A)

To estimate the age of LINEs, we used the percent mutations from RepBase consensus sequences of each element as reported by RepeatMasker (Repeat masking and classification of transposable elements). We extracted all LINE L2 and L1 matches in the genome and characterized them as regulatorily inactive if they did not overlap a regulatory region we identified in this project, as recently evolved regulatory region if they were recently evolved (The recently evolved and maintained regulomes across species (Fig. 3a)), and evolutionarily dynamic regulatory signature if we had found them to align to a regulatory region of another signature (Evolutionary dynamic regulatory signatures (Fig. 4b and Figure S5)). For Fig. 5d, we plotted the mutations for all species combined, while Additional file 1: Figure S10A shows the same data but split by the species it was identified in. The *p* values were calculated with the ggpubr package in R [94], using the stat_compare_means function and a one-sided Wilcoxon test between all pairs of categories (regulatorily inactive, recently evolved, and evolutionarily dynamic regulatory region).

### Relative transposable element enrichment of evolutionarily dynamic and stable regulatory signatures (Fig. 5e)

We first extracted all evolutionarily dynamic regulatory regions, i.e., evolutionarily dynamic promoters and evolutionarily dynamic enhancers (Evolutionary dynamic regulatory signatures (Fig. 4B and Figure S5)). To calculate the relative enrichment of evolutionarily dynamic regions (switch regulatory regions) to those not found to be evolutionarily dynamic (stable regulatory regions), we performed calculations similar to the recently evolved relative enrichment (Relative transposable element enrichment of tissue-specific and tissue-shared recently evolved regulatory and maintained regions (Fig. 5a and Figure S9B, Tables S6 and S7)) but changing the groups of regulatory regions being compared. To generate the relative enrichment shown in Fig. 5e, for each category of regulatory region, we subtracted the percentage of stable regulatory regions belonging to a subgroup from the percentage of evolutionarily dynamic regulatory regions. Consequently, positive values indicate a higher proportion of that subgroup in the evolutionarily dynamic than in stable regulatory regions. For example, rat has a value of − 11 for L1 active enhancers because 63% of all evolutionarily dynamic active enhancers overlapping a LINE belonged to the L1 subgroup, compared to 74% of the stable active enhancers. The heatmap of all relative enrichments in Fig. 5e was generated using the heatmap.2() function in gplots package version 3.0.1.1 [97]. The *p* values were calculated on the original counts, using the *Z*-test in R base function prop.test and Bonferroni correction using the total number of tests across the matrix implemented in R base function p.adjust. For generating the heatmaps images, we filtered all possible subgroups to include only those that have at least 100 tissue-specific and 100 tissue-shared occurrences in any species and manually refined the selection to only include those that are informative for multiple lineages, but total counts across all subgroups were used for the *p* value calculations.

### Multimapping and unique reads (Additional file 7: Table S8)

To compare the proportion of reads mapping uniquely in the non-repetitive genome and within LINEs, we found all multimapping reads in three genomic categories. For the non-repetitive genome, we found all reads that were not masked by RepeatMasker as simple repeats or transposable elements (Repeat masking and classification of transposable elements). Next, we found all reads overlapping LINE L1s and LINE L2 fragments as determined by overlap with RepeatMasker annotations. We counted those duplicate removed reads that were marked by bwa with the “XA:Z” flag as unique (ChIP-seq mapping (Figures S1 and S2)).

### Constrained element content in tissue-specific LINEs (Figures S10D and E)

To examine the difference in sequence constraint within regulatorily active LINE transposable elements and avoid bias, we focused on those LINE elements that overlapped tissue-specific regulatory regions in all species. For Additional file 1: Figure S10D for each tissue-specific regulatory region associated with a LINE L1 or L2, we extracted the GERP rejected substation scores [98] from Ensembl version 98 [90]. Briefly, unalignable genomic regions do not have a GERP score, while a negative score in alignable indicates more sequence constraint than expected and a positive score indicates less. We plotted the resulting graphs in R version 3.6.2 [91] using ggplot2 version 3.1.1 [92] and calculated *p* values using the base R wilcox.test.

For Additional file 1: Figure S10E, we extracted the number of constrained elements, i.e., short genomic regions that have more sequence constraint than expected [98], for each tissue-specific regulatory region associated with a LINE L1 or L2 from Ensembl version 98 [90]. Unlike the analysis reported in Fig. 5c, this includes also unalignable genomic regions. We plotted the resulting graphs in R version 3.6.2 [91] using ggplot2 version 3.1.1 [92] and calculated *p* values using the base R chisq.test.

## Supplementary Information

**Additional file 1.** Supplementary figures and supplementary Table S1 and S4.

**Additional file 2: Table S2.** All tissues used in the study. Detailed description of all tissues samples used in the study, including the unique identifiers for three histone ChIP-seq libraries, the corresponding input libraries and RNA-seq libraries sequenced from each individual.

**Additional file 3: Table S3.** ChIP-seq mapping statistics. The number of reads sequenced, mapped, passing quality control and after duplicate removal for all ChIP-seq and input libraries used in this study.

**Additional file 4: Table S5.** RNA-seq mapping statistics. The number of sequenced, mapped, and multimapping RNA-seq reads for all libraries used in this study.

**Additional file 5: Table S6.** Recently evolved regulatory regions in transposable elements. Counts of recently evolved regulatory regions overlapping different repeat groups use for enrichment analyses in Fig. 5a.

**Additional file 6: Table S7.** Maintained regulatory regions in transposable elements. Counts of maintained regulatory regions overlapping different repeat groups used for enrichment analyses in Figure S10A.

**Additional file 7: Table S8.** Multimapping reads. The percentage of quality controlled reads, i.e. MAPQ > 1 and duplicate removed, that map uniquely within non-repetitive genomic regions and LINE L1 and L2 genomic regions.

**Additional file 8:** **Table S9.** Evolutionarily dynamic regulatory regions in transposable elements. Counts of evolutionarily dynamic regulatory regions overlapping different repeat groups use for enrichment analyses in Fig. 5e.

**Additional file 9: Data S1.** The standalone ZIP file containing custom scripts and pipelines produced for this study.

**Additional file 10.** Review history.

## Data Availability

The datasets supporting the conclusions of this article are available in the ArrayExpress repository (https://www.ebi.ac.uk/arrayexpress/). The ChIP-seq datasets have accession number E-MTAB-7127 (https://www.ebi.ac.uk/arrayexpress/E-MTAB-7127) [99], and matched RNA-seq experiments E-MTAB-8122 (https://www.ebi.ac.uk/arrayexpress/E-MTAB-8122) [100]. Direct links to all datasets and other relevant material is available from https://www.ebi.ac.uk/research/flicek/publications/FOG29.

## References

[1] Aguet F, Barbeira AN, Bonazzola R, Brown A, Castel SE, Jo B, Kasela S, Kim-Hellmuth S, Liang Y, Oliva M, et al. The GTEx Consortium. The GTEx Consortium atlas of genetic regulatory effects across human tissues. Science. 2020;369:1318–30. 10.1126/science.aaz1776 PMC7737656 32913098

[2] Andersson R, Sandelin A (2020). Determinants of enhancer and promoter activities of regulatory elements. Nat Rev Genet.

[3] Leung D, Jung I, Rajagopal N, Schmitt A, Selvaraj S, Lee AY, Yen CA, Lin S, Lin Y, Qiu Y (2015). Integrative analysis of haplotype-resolved epigenomes across human tissues. Nature.

[4] Dao LTM, Galindo-Albarran AO, Castro-Mondragon JA, Andrieu-Soler C, Medina-Rivera A, Souaid C, Charbonnier G, Griffon A, Vanhille L, Stephen T (2017). Genome-wide characterization of mammalian promoters with distal enhancer functions. Nat Genet.

[5] Jung I, Schmitt A, Diao Y, Lee AJ, Liu T, Yang D, Tan C, Eom J, Chan M, Chee S (2019). A compendium of promoter-centered long-range chromatin interactions in the human genome. Nat Genet.

[6] Andersson R, Gebhard C, Miguel-Escalada I, Hoof I, Bornholdt J, Boyd M, Chen Y, Zhao X, Schmidl C, Suzuki T (2014). An atlas of active enhancers across human cell types and tissues. Nature.

[7] De Santa F, Barozzi I, Mietton F, Ghisletti S, Polletti S, Tusi BK, Muller H, Ragoussis J, Wei CL, Natoli G (2010). A large fraction of extragenic RNA pol II transcription sites overlap enhancers. PLoS Biol.

[8] Kim TK, Hemberg M, Gray JM, Costa AM, Bear DM, Wu J, Harmin DA, Laptewicz M, Barbara-Haley K, Kuersten S (2010). Widespread transcription at neuronal activity-regulated enhancers. Nature.

[9] Kowalczyk MS, Hughes JR, Garrick D, Lynch MD, Sharpe JA, Sloane-Stanley JA, McGowan SJ, De Gobbi M, Hosseini M, Vernimmen D (2012). Intragenic enhancers act as alternative promoters. Mol Cell.

[10] Carelli FN, Liechti A, Halbert J, Warnefors M, Kaessmann H (2018). Repurposing of promoters and enhancers during mammalian evolution. Nat Commun.

[11] Brawand D, Soumillon M, Necsulea A, Julien P, Csardi G, Harrigan P, Weier M, Liechti A, Aximu-Petri A, Kircher M (2011). The evolution of gene expression levels in mammalian organs. Nature.

[12] Cardoso-Moreira M, Halbert J, Valloton D, Velten B, Chen C, Shao Y, Liechti A, Ascencao K, Rummel C, Ovchinnikova S (2019). Gene expression across mammalian organ development. Nature.

[13] Barbosa-Morais NL, Irimia M, Pan Q, Xiong HY, Gueroussov S, Lee LJ, Slobodeniuc V, Kutter C, Watt S, Çolak R (2012). The evolutionary landscape of alternative splicing in vertebrate species. Science.

[14] Villar D, Berthelot C, Aldridge S, Rayner TF, Lukk M, Pignatelli M, Park TJ, Deaville R, Erichsen JT, Jasinska AJ (2015). Enhancer evolution across 20 mammalian species. Cell.

[15] Swain-Lenz D, Berrio A, Safi A, Crawford GE, Wray GA (2019). Comparative analyses of chromatin landscape in White adipose tissue suggest humans may have less Beigeing potential than other Primates. Genome Biol Evol.

[16] Glinsky G, Barakat TS (2019). The evolution of Great Apes has shaped the functional enhancers’ landscape in human embryonic stem cells. Stem Cell Res.

[17] Danko CG, Choate LA, Marks BA, Rice EJ, Wang Z, Chu T, Martins AL, Dukler N, Coonrod SA, Tait Wojno ED (2018). Dynamic evolution of regulatory element ensembles in primate CD4(+) T cells. Nat Ecol Evol.

[18] Cheng Y, Ma Z, Kim BH, Wu W, Cayting P, Boyle AP, Sundaram V, Xing X, Dogan N, Li J (2014). Principles of regulatory information conservation between mouse and human. Nature.

[19] Vierstra J, Rynes E, Sandstrom R, Zhang M, Canfield T, Hansen RS, Stehling-Sun S, Sabo PJ, Byron R, Humbert R (2014). Mouse regulatory DNA landscapes reveal global principles of cis-regulatory evolution. Science.

[20] Donnard E, Vangala P, Afik S, McCauley S, Nowosielska A, Kucukural A, Tabak B, Zhu X, Diehl W, McDonel P (2018). Comparative analysis of immune cells reveals a conserved regulatory lexicon. Cell Syst.

[21] Forrest AR, Kawaji H, Rehli M, Baillie JK, de Hoon MJ, Haberle V, Lassmann T, Kulakovskiy IV, Lizio M, Itoh M (2014). A promoter-level mammalian expression atlas. Nature.

[22] Young RS, Hayashizaki Y, Andersson R, Sandelin A, Kawaji H, Itoh M, Lassmann T, Carninci P, Bickmore WA, Forrest AR, Taylor MS (2015). The frequent evolutionary birth and death of functional promoters in mouse and human. Genome Res.

[23] Bourque G, Burns KH, Gehring M, Gorbunova V, Seluanov A, Hammell M, Imbeault M, Izsvák Z, Levin HL, Macfarlan TS (2018). Ten things you should know about transposable elements. Genome Biol.

[24] Jacques PE, Jeyakani J, Bourque G (2013). The majority of primate-specific regulatory sequences are derived from transposable elements. PLoS Genet.

[25] Chuong EB, Elde NC, Feschotte C (2016). Regulatory evolution of innate immunity through co-option of endogenous retroviruses. Science.

[26] Franke V, Ganesh S, Karlic R, Malik R, Pasulka J, Horvat F, Kuzman M, Fulka H, Cernohorska M, Urbanova J (2017). Long terminal repeats power evolution of genes and gene expression programs in mammalian oocytes and zygotes. Genome Res.

[27] Trizzino M, Kapusta A, Brown CD (2018). Transposable elements generate regulatory novelty in a tissue-specific fashion. BMC Genomics.

[28] Trizzino M, Park Y, Holsbach-Beltrame M, Aracena K, Mika K, Caliskan M, Perry GH, Lynch VJ, Brown CD (2017). Transposable elements are the primary source of novelty in primate gene regulation. Genome Res.

[29] Cao Y, Chen G, Wu G, Zhang X, McDermott J, Chen X, Xu C, Jiang Q, Chen Z, Zeng Y (2019). Widespread roles of enhancer-like transposable elements in cell identity and long-range genomic interactions. Genome Res.

[30] Platt RN, Vandewege MW, Ray DA (2018). Mammalian transposable elements and their impacts on genome evolution. Chromosom Res.

[31] Chalopin D, Naville M, Plard F, Galiana D, Volff JN (2015). Comparative analysis of transposable elements highlights mobilome diversity and evolution in vertebrates. Genome Biol Evol.

[32] Elbarbary RA, Lucas BA, Maquat LE (2016). Retrotransposons as regulators of gene expression. Science.

[33] Belancio VP, Roy-Engel AM, Pochampally RR, Deininger P (2010). Somatic expression of LINE-1 elements in human tissues. Nucleic Acids Res.

[34] Guffanti G, Bartlett A, Klengel T, Klengel C, Hunter R, Glinsky G, Macciardi F (2018). Novel bioinformatics approach identifies transcriptional profiles of lineage-specific transposable elements at distinct loci in the human dorsolateral prefrontal cortex. Mol Biol Evol.

[35] Philippe C, Vargas-Landin DB, Doucet AJ, van Essen D, Vera-Otarola J, Kuciak M, Corbin A, Nigumann P, Cristofari G (2016). Activation of individual L1 retrotransposon instances is restricted to cell-type dependent permissive loci. eLife.

[36] Deininger P, Morales ME, White TB, Baddoo M, Hedges DJ, Servant G, Srivastav S, Smither ME, Concha M, DeHaro DL (2017). A comprehensive approach to expression of L1 loci. Nucleic Acids Res.

[37] Yang Z, Boffelli D, Boonmark N, Schwartz K, Lawn R (1998). Apolipoprotein(a) gene enhancer resides within a LINE element. J Biol Chem.

[38] Petri R, Brattas PL, Sharma Y, Jonsson ME, Pircs K, Bengzon J, Jakobsson J (2019). LINE-2 transposable elements are a source of functional human microRNAs and target sites. PLoS Genet.

[39] Huda A, Tyagi E, Marino-Ramirez L, Bowen NJ, Jjingo D, Jordan IK (2011). Prediction of transposable element derived enhancers using chromatin modification profiles. PLoS One.

[40] Yates AD, Achuthan P, Akanni W, Allen J, Allen J, Alvarez-Jarreta J, Amode MR, Armean IM, Azov AG, Bennett R (2020). Ensembl 2020. Nucleic Acids Res.

[41] Shen Y, Yue F, McCleary DF, Ye Z, Edsall L, Kuan S, Wagner U, Dixon J, Lee L, Lobanenkov VV, Ren B (2012). A map of the cis-regulatory sequences in the mouse genome. Nature.

[42] Bernstein BE, Kamal M, Lindblad-Toh K, Bekiranov S, Bailey DK, Huebert DJ, McMahon S, Karlsson EK, Kulbokas EJ, Gingeras TR (2005). Genomic maps and comparative analysis of histone modifications in human and mouse. Cell.

[43] Creyghton MP, Cheng AW, Welstead GG, Kooistra T, Carey BW, Steine EJ, Hanna J, Lodato MA, Frampton GM, Sharp PA (2010). Histone H3K27ac separates active from poised enhancers and predicts developmental state. Proc Natl Acad Sci U S A.

[44] Calo E, Wysocka J (2013). Modification of enhancer chromatin: what, how, and why?. Mol Cell.

[45] Schoenfelder S, Fraser P (2019). Long-range enhancer-promoter contacts in gene expression control. Nat Rev Genet.

[46] Wang A, Yue F, Li Y, Xie R, Harper T, Patel NA, Muth K, Palmer J, Qiu Y, Wang J (2015). Epigenetic priming of enhancers predicts developmental competence of hESC-derived endodermal lineage intermediates. Cell Stem Cell.

[47] Aken BL, Ayling S, Barrell D, Clarke L, Curwen V, Fairley S, Fernandez Banet J, Billis K, Garcia Giron C, Hourlier T, et al. The Ensembl gene annotation system. Database (Oxford). 2016;baw093. 10.1093/database/baw093 PMC4919035 27337980

[48] Darmanis S, Sloan SA, Zhang Y, Enge M, Caneda C, Shuer LM, Hayden Gephart MG, Barres BA, Quake SR (2015). A survey of human brain transcriptome diversity at the single cell level. Proc Natl Acad Sci U S A.

[49] Vermunt MW, Tan SC, Castelijns B, Geeven G, Reinink P, de Bruijn E, Kondova I, Persengiev S, Bontrop R, Cuppen E (2016). Epigenomic annotation of gene regulatory alterations during evolution of the primate brain. Nat Neurosci.

[50] Soumillon M, Necsulea A, Weier M, Brawand D, Zhang X, Gu H, Barthes P, Kokkinaki M, Nef S, Gnirke A (2013). Cellular source and mechanisms of high transcriptome complexity in the mammalian testis. Cell Rep.

[51] Xia B, Yan Y, Baron M, Wagner F, Barkley D, Chiodin M, Kim SY, Keefe DL, Alukal JP, Boeke JD, Yanai I (2020). Widespread transcriptional scanning in the testis modulates gene evolution rates. Cell.

[52] Heintzman ND, Hon GC, Hawkins RD, Kheradpour P, Stark A, Harp LF, Ye Z, Lee LK, Stuart RK, Ching CW (2009). Histone modifications at human enhancers reflect global cell-type-specific gene expression. Nature.

[53] Mifsud B, Tavares-Cadete F, Young AN, Sugar R, Schoenfelder S, Ferreira L, Wingett SW, Andrews S, Grey W, Ewels PA (2015). Mapping long-range promoter contacts in human cells with high-resolution capture Hi-C. Nat Genet.

[54] Hait TA, Amar D, Shamir R, Elkon R (2018). FOCS: a novel method for analyzing enhancer and gene activity patterns infers an extensive enhancer-promoter map. Genome Biol.

[55] Fish A, Chen L, Capra JA (2017). Gene regulatory enhancers with evolutionarily conserved activity are more pleiotropic than those with species-specific activity. Genome Biol Evol.

[56] Thybert D, Roller M, Navarro FCP, Fiddes I, Streeter I, Feig C, Martin-Galvez D, Kolmogorov M, Janousek V, Akanni W (2018). Repeat associated mechanisms of genome evolution and function revealed by the Mus caroli and Mus pahari genomes. Genome Res.

[57] Lovsin N, Gubensek F, Kordi D (2001). Evolutionary dynamics in a novel L2 clade of non-LTR retrotransposons in Deuterostomia. Mol Biol Evol.

[58] Necsulea A, Kaessmann H (2014). Evolutionary dynamics of coding and non-coding transcriptomes. Nat Rev Genet.

[59] Simonti CN, Pavlicev M, Capra JA (2017). Transposable element exaptation into regulatory regions is rare, influenced by evolutionary age, and subject to pleiotropic constraints. Mol Biol Evol.

[60] Schmidt D, Wilson MD, Spyrou C, Brown GD, Hadfield J, Odom DT (2009). ChIP-seq: using high-throughput sequencing to discover protein-DNA interactions. Methods.

[61] Aldridge S, Watt S, Quail MA, Rayner T, Lukk M, Bimson MF, Gaffney D, Odom DT (2013). AHT-ChIP-seq: a completely automated robotic protocol for high-throughput chromatin immunoprecipitation. Genome Biol.

[62] Li H, Durbin R (2009). Fast and accurate short read alignment with Burrows-Wheeler transform. Bioinformatics.

[63] Li H, Handsaker B, Wysoker A, Fennell T, Ruan J, Homer N, Marth G, Abecasis G, Durbin R (2009). The sequence alignment/map format and SAMtools. Bioinformatics.

[64] Landt SG, Marinov GK, Kundaje A, Kheradpour P, Pauli F, Batzoglou S, Bernstein BE, Bickel P, Brown JB, Cayting P (2012). ChIP-seq guidelines and practices of the ENCODE and modENCODE consortia. Genome Res.

[65] Zhang Y, Liu T, Meyer CA, Eeckhoute J, Johnson DS, Bernstein BE, Nusbaum C, Myers RM, Brown M, Li W, Liu XS (2008). Model-based analysis of ChIP-Seq (MACS). Genome Biol.

[66] Heintzman ND, Stuart RK, Hon G, Fu Y, Ching CW, Hawkins RD, Barrera LO, Van Calcar S, Qu C, Ching KA (2007). Distinct and predictive chromatin signatures of transcriptional promoters and enhancers in the human genome. Nat Genet.

[67] Rada-Iglesias A, Bajpai R, Swigut T, Brugmann SA, Flynn RA, Wysocka J (2011). A unique chromatin signature uncovers early developmental enhancers in humans. Nature.

[68] Santos-Rosa H, Schneider R, Bannister AJ, Sherriff J, Bernstein BE, Emre NC, Schreiber SL, Mellor J, Kouzarides T (2002). Active genes are tri-methylated at K4 of histone H3. Nature.

[69] Schneider R, Bannister AJ, Myers FA, Thorne AW, Crane-Robinson C, Kouzarides T (2004). Histone H3 lysine 4 methylation patterns in higher eukaryotic genes. Nat Cell Biol.

[70] Kim TH, Barrera LO, Zheng M, Qu C, Singer MA, Richmond TA, Wu Y, Green RD, Ren B (2005). A high-resolution map of active promoters in the human genome. Nature.

[71] Wang Z, Zang C, Rosenfeld JA, Schones DE, Barski A, Cuddapah S, Cui K, Roh TY, Peng W, Zhang MQ, Zhao K (2008). Combinatorial patterns of histone acetylations and methylations in the human genome. Nat Genet.

[72] Harris RS. Improved pairwise alignment of genomic data [Doctoral dissertation]. College Park: Pennsylvania State University; 2007.

[73] Frankish A, Diekhans M, Ferreira AM, Johnson R, Jungreis I, Loveland J, Mudge JM, Sisu C, Wright J, Armstrong J (2019). GENCODE reference annotation for the human and mouse genomes. Nucleic Acids Res.

[74] Li H (2018). Minimap2: pairwise alignment for nucleotide sequences. Bioinformatics.

[75] Griffiths-Jones S, Bateman A, Marshall M, Khanna A, Eddy SR (2003). Rfam: an RNA family database. Nucleic Acids Res.

[76] Griffiths-Jones S, Grocock RJ, van Dongen S, Bateman A, Enright AJ (2006). miRBase: microRNA sequences, targets and gene nomenclature. Nucleic Acids Res.

[77] Altschul SF, Gish W, Miller W, Myers EW, Lipman DJ (1990). Basic local alignment search tool. J Mol Biol.

[78] Lorenz R, Bernhart SH, Honer Zu Siederdissen C, Tafer H, Flamm C, Stadler PF, Hofacker IL (2011). ViennaRNA Package 2.0. Algorithms Mol Biol.

[79] Nawrocki EP, Eddy SR (2013). Infernal 1.1: 100-fold faster RNA homology searches. Bioinformatics.

[80] Bolger AM, Lohse M, Usadel B (2014). Trimmomatic: a flexible trimmer for Illumina sequence data. Bioinformatics.

[81] Dobin A, Davis CA, Schlesinger F, Drenkow J, Zaleski C, Jha S, Batut P, Chaisson M, Gingeras TR (2013). STAR: ultrafast universal RNA-seq aligner. Bioinformatics.

[82] Trapnell C, Williams BA, Pertea G, Mortazavi A, Kwan G, van Baren MJ, Salzberg SL, Wold BJ, Pachter L (2010). Transcript assembly and quantification by RNA-Seq reveals unannotated transcripts and isoform switching during cell differentiation. Nat Biotechnol.

[83] Zerbino DR, Johnson N, Juetteman T, Sheppard D, Wilder SP, Lavidas I, Nuhn M, Perry E, Raffaillac-Desfosses Q, Sobral D, et al. Ensembl regulation resources. Database (Oxford). 2016;bav119. 10.1093/database/bav119 PMC4756621 26888907

[84] O'Leary NA, Wright MW, Brister JR, Ciufo S, Haddad D, McVeigh R, Rajput B, Robbertse B, Smith-White B, Ako-Adjei D (2016). Reference sequence (RefSeq) database at NCBI: current status, taxonomic expansion, and functional annotation. Nucleic Acids Res.

[85] Ramirez F, Ryan DP, Gruning B, Bhardwaj V, Kilpert F, Richter AS, Heyne S, Dundar F, Manke T (2016). deepTools2: a next generation web server for deep-sequencing data analysis. Nucleic Acids Res.

[86] Zerbino DR, Johnson N, Juettemann T, Wilder SP, Flicek P (2014). WiggleTools: parallel processing of large collections of genome-wide datasets for visualization and statistical analysis. Bioinformatics.

[87] Thorvaldsdóttir H, Robinson JT, Mesirov JP (2012). Integrative Genomics Viewer (IGV): high-performance genomics data visualization and exploration. Brief Bioinform.

[88] Conway JR, Lex A, Gehlenborg N (2017). UpSetR: an R package for the visualization of intersecting sets and their properties. Bioinformatics.

[89] Love MI, Huber W, Anders S (2014). Moderated estimation of fold change and dispersion for RNA-seq data with DESeq2. Genome Biol.

[90] Herrero J, Muffato M, Beal K, Fitzgerald S, Gordon L, Pignatelli M, Vilella AJ, Searle SMJ, Amode R, Brent S, et al. Ensembl comparative genomics resources. Database. 2016;bav096 10.1093/database/bav096 PMC4761110 26896847

[91] R Core Team (2019). R: A Language and Environment for Statistical Computing.

[92] Wickham H (2016). ggplot2: Elegant Graphics for Data Analysis.

[93] Krzywinski M, Schein J, Birol I, Connors J, Gascoyne R, Horsman D, Jones SJ, Marra MA (2009). Circos: an information aesthetic for comparative genomics. Genome Res.

[94] Kassambara A (2020). ggpubr: 'ggplot2' Based Publication Ready Plots.

[95] Smit A, Hubley R, Green P. RepeatMasker Open-4.0. 2013-2015.

[96] Bao W, Kojima KK, Kohany O (2015). Repbase update, a database of repetitive elements in eukaryotic genomes. Mob DNA.

[97] Warnes GR, Bolker B, Bonebakker L, Gentleman R, Huber W, Liaw A, Lumley T, Maechler M, Magnusson A, Moeller S (2019). gplots: various R programming tools for plotting data.

[98] Cooper GM, Stone EA, Asimenos G, Green ED, Batzoglou S, Sidow A (2005). Distribution and intensity of constraint in mammalian genomic sequence. Genome Res.

[99] Roller M, Stamper E, Villar D, Izuogu O, Martin F, Redmond AM, Ramachanderan R, Harewood L, Odom DT, Flicek P: H3K4me3, H3K27ac and H3K4me1 ChIP-seq in 4 tissues of 10 mammals. E-MTAB-7127. ArrayExpress. https://www.ebi.ac.uk/arrayexpress/experiments/E-MTAB-7127 (2020).

[100] Roller M, Stamper E, Villar D, Izuogu O, Martin F, Redmond AM, Ramachanderan R, Harewood L, Odom DT, Flicek P: RNA-seq in 4 tissues of 10 mammals. E-MTAB-8122. ArrayExpress. https://www.ebi.ac.uk/arrayexpress/E-MTAB-8122 (2019).

