# Supplementary material for: LINE retrotransposons characterize mammalian tissue-specific and evolutionarily dynamic regulatory regions
Source: Genome Biol. 2021 Feb 18;22:62. doi: 10.1186/s13059-021-02260-y (PMC7890895; doi:10.1186/s13059-021-02260-y)
Supplement: Supplementary file 1 — Additional file 1. Supplementary figures and supplementary Table S1 and S4. [file 13059_2021_2260_MOESM1_ESM.docx]

**SUPPLEMENTAL FIGURES AND LEGENDS**


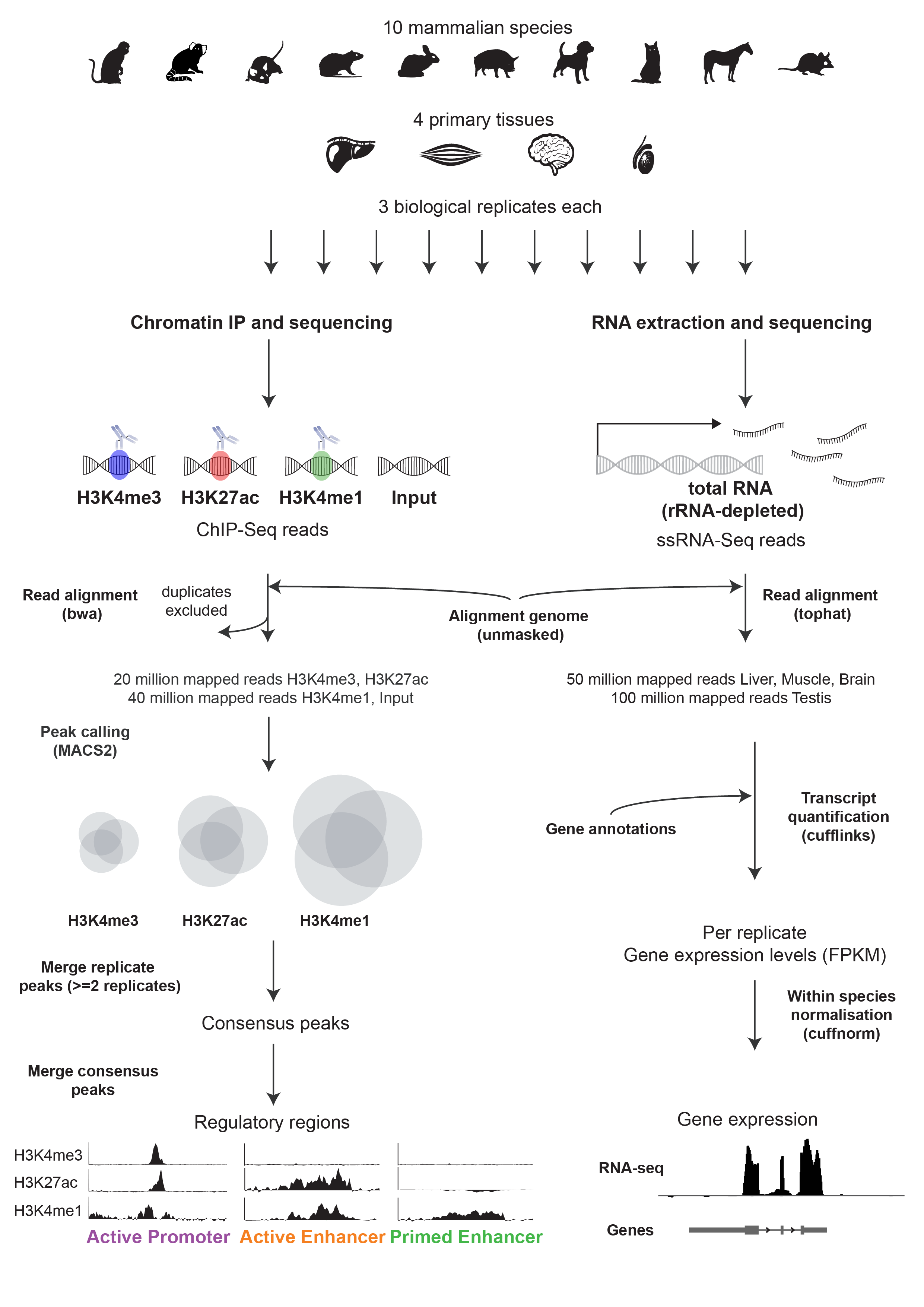
**Fig. S1: Experimental Overview.**

Functional genomics experiments were performed on 10 mammalian species (macaque, marmoset, mouse, rat, rabbit, pig, dog, cat, horse, opossum) and 4 primary adult tissues (liver, muscle, brain, and testis), with 3 biological replicates (individuals) for each. ChIP-seq for 3 different histone modifications (H3K4me3, H3K27ac, and H3K4me1) was used these to identify regulatory regions genome wide (active promoters, active enhancers, and primed enhancers). Within each biological replicate, ChIP-seq and input libraries were mapped, duplicate reads were removed, but multi-maping reads were retained to aid mapping across transposable elements, and then randomly subsampled to the same depth before peak calling. For each histone modification, only those peaks present in at least two replicates (consensus) were merged and kept for further analyses. Finally, regulatory regions were defined from the overlap of consensus peaks for the three histone modifications. Total RNA-seq was performed on matched tissue samples and mapped to the respective genome. Within each biological replicate, RNA-seq libraries were subsampled to the same depth per tissue. The subsampled sets were used to quantify gene expression levels within each replicate, and these were normalized within each species. (See Figure 1 for data overview.)


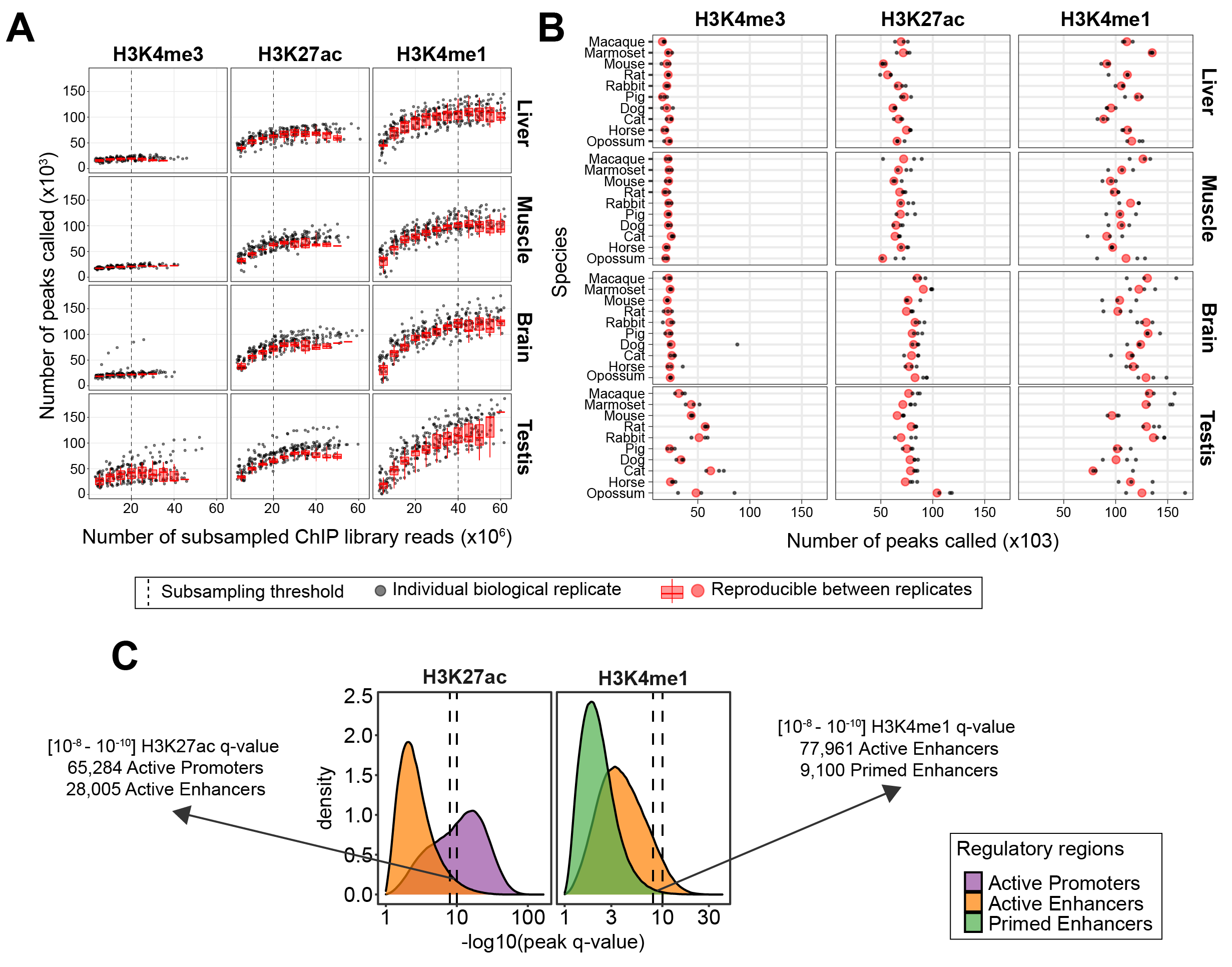


**Fig. S2: Saturating ChIP-seq signal ensures high reproducibility between replicates**.

A) To choose an appropriate subsampling threshold for all species, tissues and histone marks, ChIP-seq libraries were randomly subsampled across all replicates, and peaks per replicate called. Within each species and tissue, we also calculated the number of reproducible regions (those which appear in two or more replicates) called for each histone modification. H3K4me3 and H3K27ac signal saturated at 20 million reads in all tissues, while 40 million reads were needed to saturate H3K4me1 signal. ChIP-seq signal reached saturation in all species and tissues.

B) Peaks were called within each biological replicate using ChIP-seq samples subsampled to the appropriate thresholds for each histone modification (Figure S1, S2A). Biologically reproducible peaks were then defined as those that were found in at least two biological replicates. H3K4me3 and H3K27ac had almost no variability between replicates, while H3K4me1 was more variable but still very reproducible.

C) To compare the enrichment of histone peaks underlying regulatory region calls in this study, we compared the MACS2 q-vaules by computing the average q-value across all biological replicates overlapping a regulatory region. We focused our comparisons on those histone peaks that are shared between regulatory regions (see also Figure S1). Specifically, we compared the q-values of H3K27ac between active promoters and enhancers, and H3K4me1 peaks between active and primed enhancers. Active promoters overlap H3K27ac histone peaks with higher enrichment than active enhancers, and active enhancer similarly overlap H3K4me1 histone peaks with higher enrichment than primed enhancers. However, there is still a significant overlap of peaks with comparable enrichment between both active promoters and active enhancers, and between active and primed enhancers.

­­

**
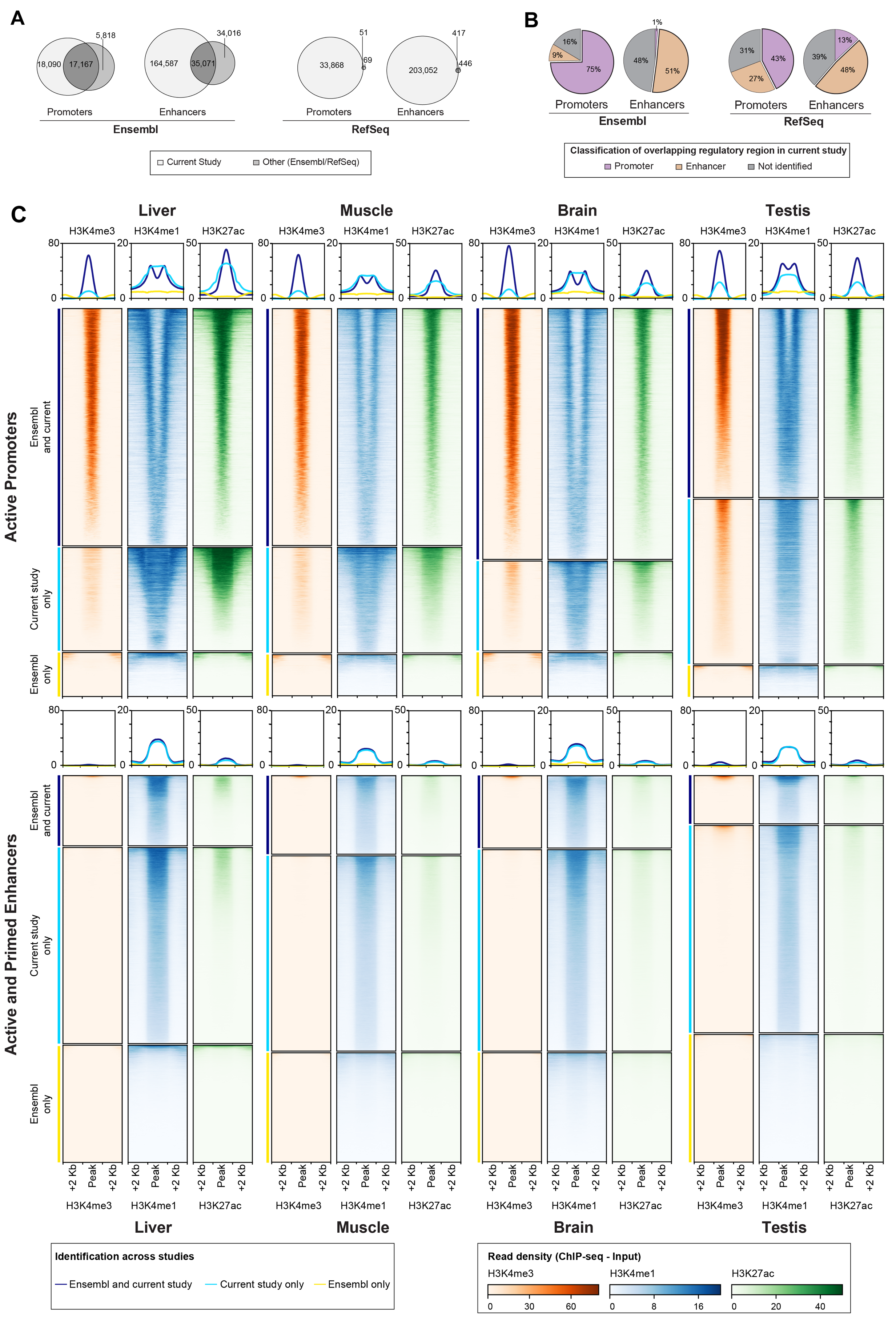
**

**Fig. S3: Validation of ChIP-seq and regulatory regions in Mouse** (See also Figure 1).

A) Venn diagrams showing overlap between: (left) the promoters and enhancers called in the current study and promoters and enhancers in the Ensembl regulatory build [77],); and (right) promoters and enhancers experimentally validated and listed in RefSeq [78]. For this analysis, we grouped active and primed enhancers together to make them comparable to the previous studies. The regulatory landscape of four mouse tissues from the current study recovers 75% of known mouse promoters and enhancers.

B) Further details on regulatory regions from external databases shown in B). The recovered regulatory regions are mostly consistently called as the same regulatory type in external datasets and our own data. 75% of Ensembl promoters and 51% of Ensembl enhancers are consistent with our own calls, while the agreement with RefSeq is 43% for promoters and 48% RefSeq for enhancers.

C) We compared the ChIP-seq read enrichments for promoters and enhancers that were uniquely identified here (Current study only, panel B) to those that overlap regulatory regions called in the Ensembl regulatory build (Ensembl and current study, panel A), and those called in the Ensembl regulatory build but not identified here (Ensembl only, panel A). ChIP-seq read enrichments are shown for regions we defined as active promoters, and jointly for regions we describe as active and primed enhancers, given that Ensembl does not make this distinction. The top panel shows density plots of average fold enrichment (over input) for all regions in each category for all regions in each category; the bottom panel shows fold enrichment where each row is a regulatory region identified by either this study or other studies defined in S3A and B. The length of each regulatory region was normalized before adding uniform 2 Kb flanking sequences upstream and downstream of regulatory regions. ChIP-seq read enrichments are shown on a uniform scale across tissues and regulatory regions for each histone mark.


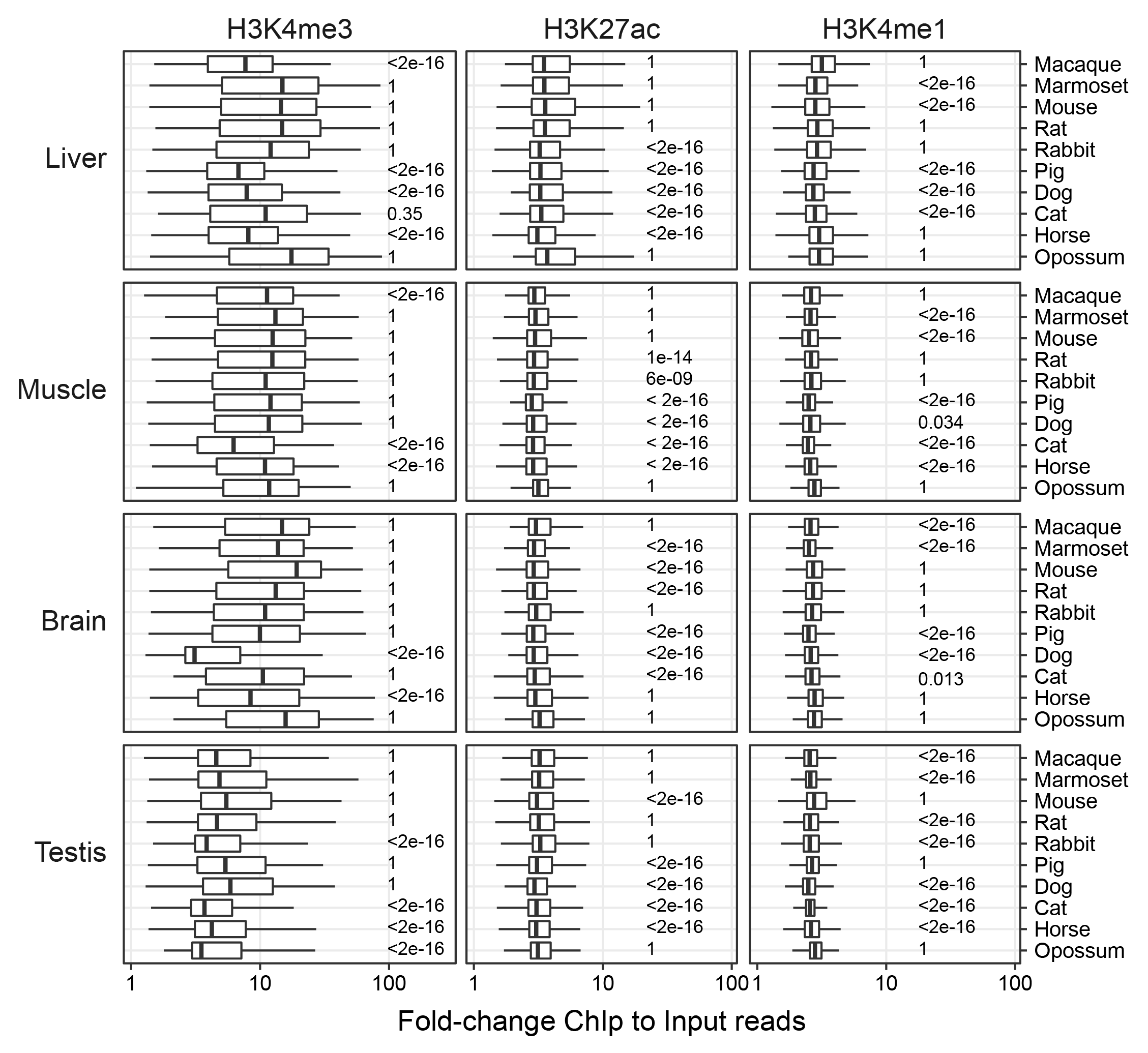


**Fig. S4: Fold-change of the number of ChIP-seq to input reads for all biological replicates**

For all peaks across all biological replicates we show the distribution of the fold-change in read counts between the ChIP-seq and input library as computed by MACS2. The libraries have been subsampled to the thresholds defined before (Figure S1, S2A, S2B). p-values shown were calculated using the Wilcoxon test for significantly lower means of fold-change for each species and histone mark compared to the average across all species. Fold-change is closely related to the total number of peaks called per replicate (Figure S2B); it is highest for H3K4me3 in somatic tissues, followed by testis H3K4me3, H3K27ac in all tissues, and finally H3K4me1 in all tissues.

**
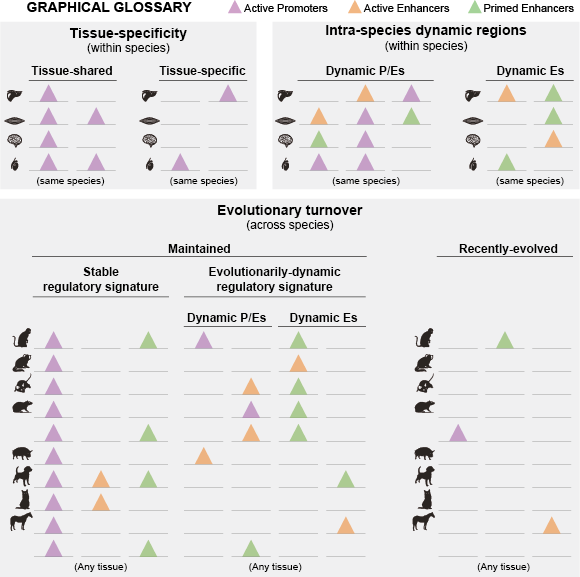
**

**Fig. S5: Graphical glossary**

Graphical glossary showing cartoon examples of how regulatory regions were categorized within a species (top panels) and across species (bottom panel). Stacked lines indicate the same genomic region across tissues (top panels) or aligned genomic regions across species (bottom panel). Triangles indicate a regulatory assignment of active promoter (purple), active enhancer (orange), or primed enhancer (green). Specifically, within a species regulatory regions were defined as either **tissue-shared** if they were identified as a regulatory region with the same signature (i.e. active promoter, active enhancer, or primed enhancer) in two or more tissues; or **tissue-specific** if they were identified as a regulatory region in only one of the four study tissues. Within a species, regulatory regions were defined as **intra-species dynamic** regions if they were identified as one type of regulatory region in one tissue, and another type of regulatory region in another tissue of the same species. Intra-species dynamic promoter/enhancers (**dynamic P/Es**) were defined as any genomic region identified as an active promoter in at least one of the four tissues, and an enhancer (active and/or primed) in at least one other tissue of the same species. Similarly, intra-species dynamic enhancers (**dynamic Es**) were defined as any genomic region identified as an active enhancer in one or more tissues and as a primed enhancer in at least one other tissue of the same species, but not if it was also found to be an active promoter in a tissue. By aligning genomic regions across species, regulatory regions were identified as either **maintained** (identified as a regulatory region in two or more species, regardless of the type of regulatory region or tissue of activity) or **recently-evolved** (identified as a regulatory region in only one of the ten study species). Maintained regions were further classified either as those with a **stable regulatory signature** (identified as the same type of regulatory region in all species where regulatorily active) or those with an **evolutionarily dynamic regulatory signature** (identified as one type of regulatory region in one or more species, and another type of regulatory region in at least one other species). Evolutionarily dynamic regulatory regions were defined as either **evolutionarily dynamic P/Es** (identified as a promoter in at least one species and an active and/or primed enhancer in at least one other species) or **evolutionarily dynamic Es** (identified as an active enhancer in at least one species and a primed enhancer in at least one other species, but never an active promoter). Multiple possible examples are shown for each regulatory region category, however these are not exclusive, as many additional scenarios are possible.

**
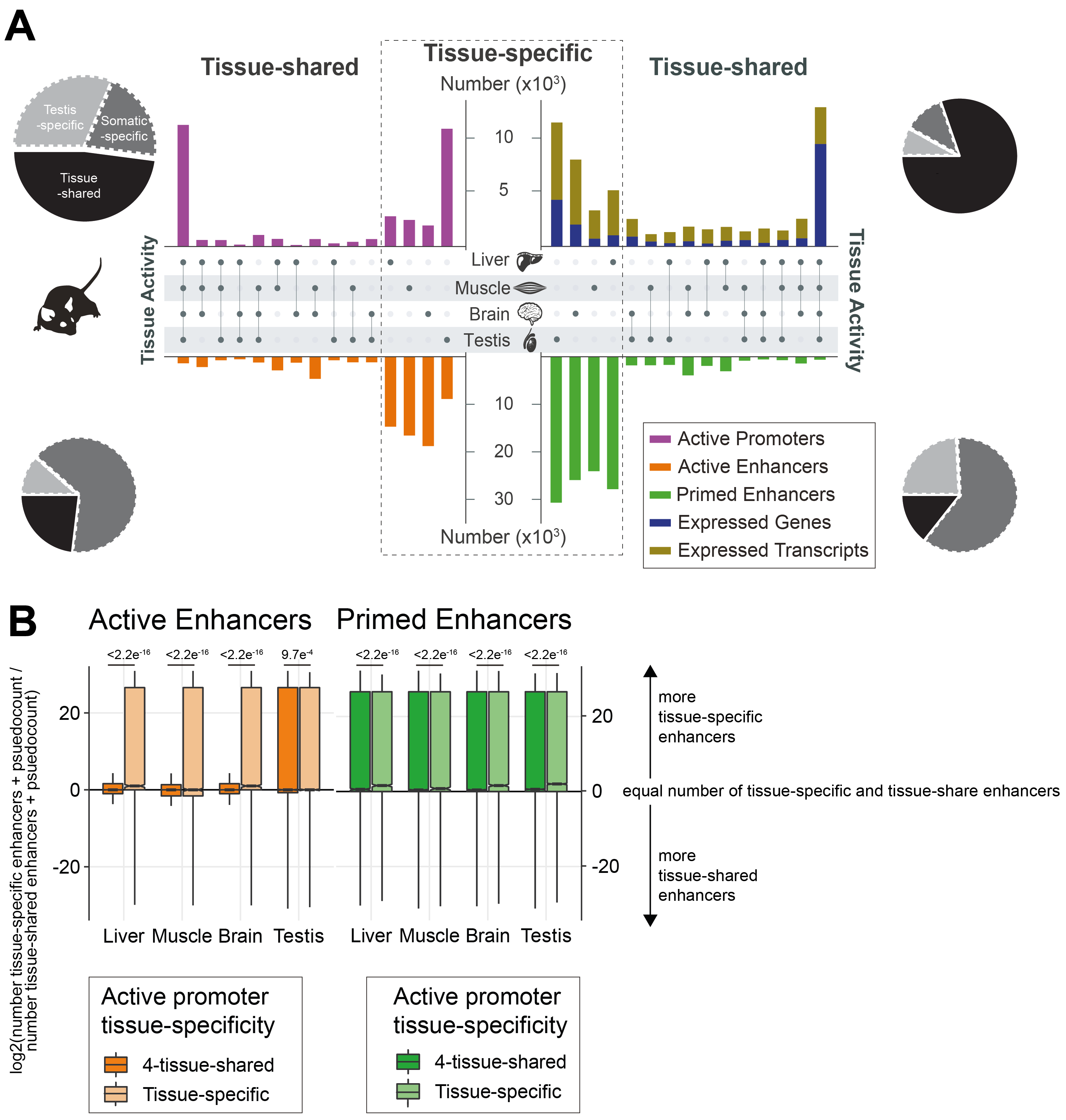
**

**Fig. S6: Tissue-specificity of regulatory regions and enhancer landscapes**

A)Tissue-specificity of regulatory regions and gene expression is shown for mouse. The bars are a summation of numbers across all ten study species while and the pie charts show the portions of tissue-shared (across any two tissues) and tissue-specific regions split by testis-specific and somatic-specific. Tissue-specific bars and pie section are outlined with a dotted line. The ratio of tissue-specific transcripts compared to genes expressed is higher than the ratio of tissue-shared transcripts to genes expressed. Analysis is the same as for Figure 2B, which shows combined values for all ten study species.

B) The ratio of the number of tissue-specific to tissue-shared active enhancers and primed enhancers associated with each active promoter are shown for the four tissues. Within each tissue, we counted the number of tissue-specific and tissue-shared enhancers associated with each active promoter and added a pseudocount of 10^-8^ to avoid dividing with zero. Next, we computed the log2 of the ratio of tissue-specific to tissue-shared enhancers. Those active promoters with an equal number of tissue-specific and tissue-shared enhancers have a log2(ratio) of 0, while those associated with more tissue-specific than tissue-shared enhancers will have an increasingly positive value, and those with more tissue-shared enhancers an increasingly negative value The Wilcoxon test was used to test whether the tissue-specific promoters within each tissue have a larger ratio than tissue-shared promoters. See also Figure 2C for an alternative graphical representation of this data.

**
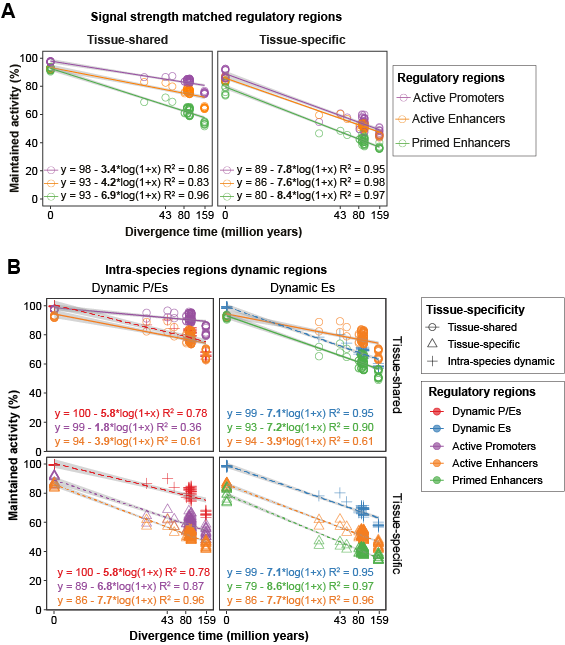
**

**Fig. S7: Additional evolutionary rates of regulatory regions**

A) To match ChIP-seq enrichment between different regulatory regions, we selected all regulatory regions that were called using a peak with a q-value between 10^-8^ and 10^-10^, selecting on H3K27ac peaks for active promoters and enhancers, and on H3K4me1 peaks for active and primed enhancers (See also Figure S2C). For these signal matched regulatory regions, we performed the same evolutionary analysis as in Figure 3B (see also Methods) and found that the tissue-shared evolve faster than their tissue-specific counterparts as evidenced through their lower slope coefficients (Two-way ANOVA of linear regression between tissue-shared and tissue-specific: active promoters p-value 1.3*10^-10^; active enhancers p-value 2.3*10^-09^), though the difference between linear regressions is not significant for primed enhancers (Two-way ANOVA of linear regression between tissue-shared and tissue-specific: p-value 0.1).

B) Similar to Figure 3B, we determined the functional conservation of intra-species dynamic regulatory regions. We compared evolutionarily dynamic promoters (red) to tissue-shared (top left) and tissue-specific (bottom left) active promoters and enhancers, and found that their evolutionary rate is intermediate between the rate of tissue-shared active promoters and enhancers. We also compared evolutionarily dynamic enhancers (blue) to tissue-shared (top right) and tissue-specific (bottom right) active and primed enhancers, and found that the rates are intermediate between tissue-shared active and primed enhancers. Evolutionary turnover rates were estimated by linear regression of activity conservation between all pairs of species for the evolutionarily dynamic regions.


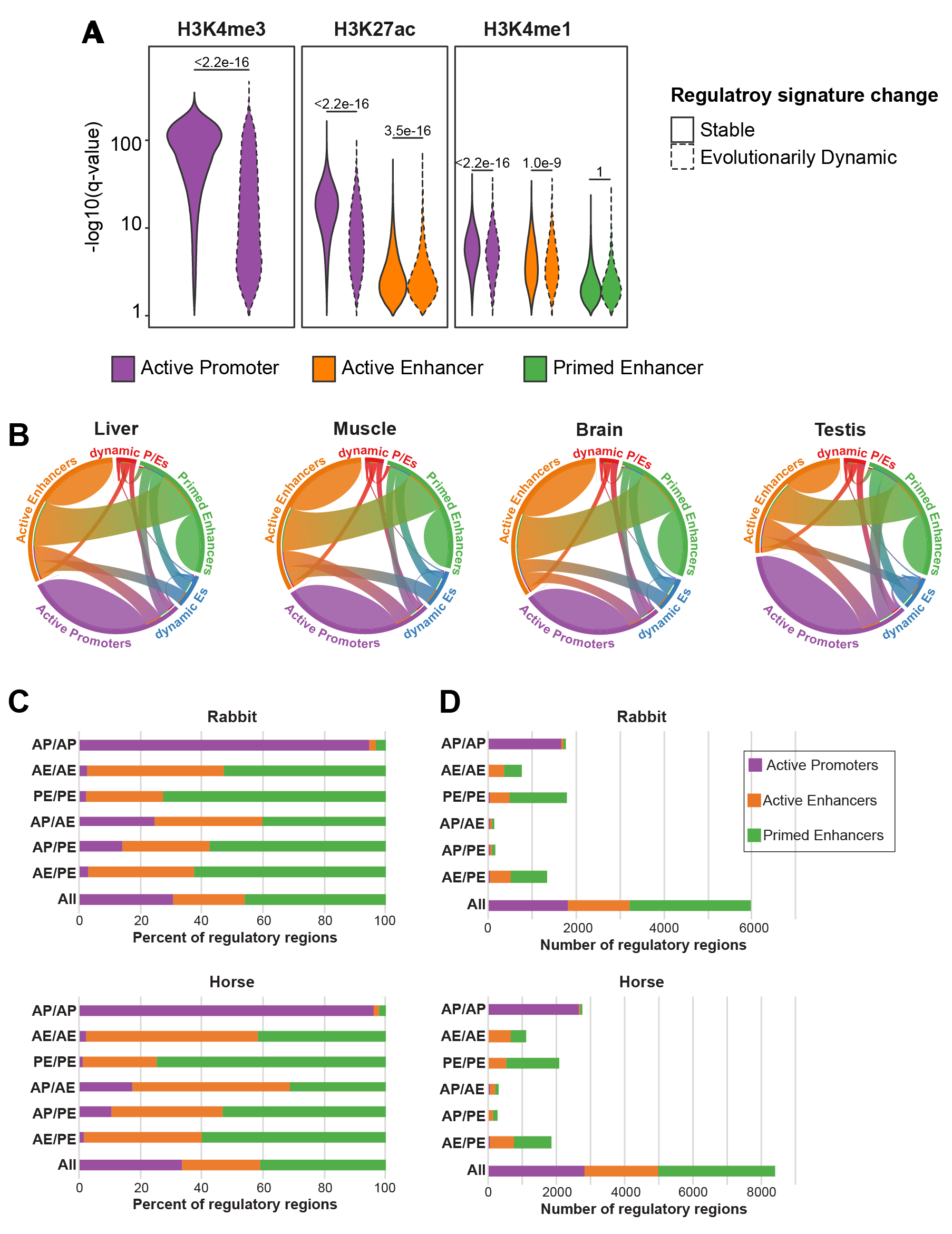


**Fig. S8: Evolutionary turnover of intra-species dynamic and evolutionarily dynamic regulatory regions.**

A) We compared the ChIP-seq signal strength of regulatory regions the switch regulatory signature between species (regulatorily dynamic), and those that are maintained but do not switch signatures (stable). We used the q-value as computed by MACS2 as a proxy for signal strength. Active promoters have weaker H3K4me3 and H3K27ac signal when evolutionarily dynamic than when they are stable. Evolutionarily dynamic and stable active enhancers have more similar H3K27ac and H3K4me1 signal strengths, while evolutionarily dynamic primed enhancers have stronger H3K4me1 signal than stable regions. P-values between evolutionary dynamic and stable regions were calculated using the z-test, testing whether stable regions have a higher mean.

B) To quantify how often regulatory signatures change between species when considering only a single tissue, we performed an analysis similar to that in Figure 2B. However, for these analyses we only considered a regulatory signature change if it occurred within the same tissue between species. For example, in a pairwise comparison between mouse and rat a signature change is when an active promoter in liver in one species is maintained as an active enhancer in liver in the other species. Within a single tissue, comparable proportion of regulatory regions align to a region with a different regulatory signature in another species (Figure 2B). On average across the tissues, 23% of pairwise comparisons with active promoters, 51% with active enhancers and 54% with primed enhancers are evolutionarily dynamic.

C) Outgroup analysis of triad species, showing results separately for mouse/rat/rabbit and cat/dog/horse (combined results are shown in Figure 4D). Given the combination of regulatory signatures in the ingroups (cat/dog or mouse/rat), we tested the signature in the outgroup species (horse or rabbit, respectively) to assay the directionality of the evolutionary change. The background distribution (All) corresponds to all regulatory regions maintained across all three species. Active enhancers often evolve from primed enhancers, and vice versa. Active promoters are more stable, but when a genomic region does change between active promoter in one species and enhancer in another, the direction is more often from enhancer to promoter. Values are shown as percentages.

D) Same as in C, but with values shown as raw numbers, rather than percentages.


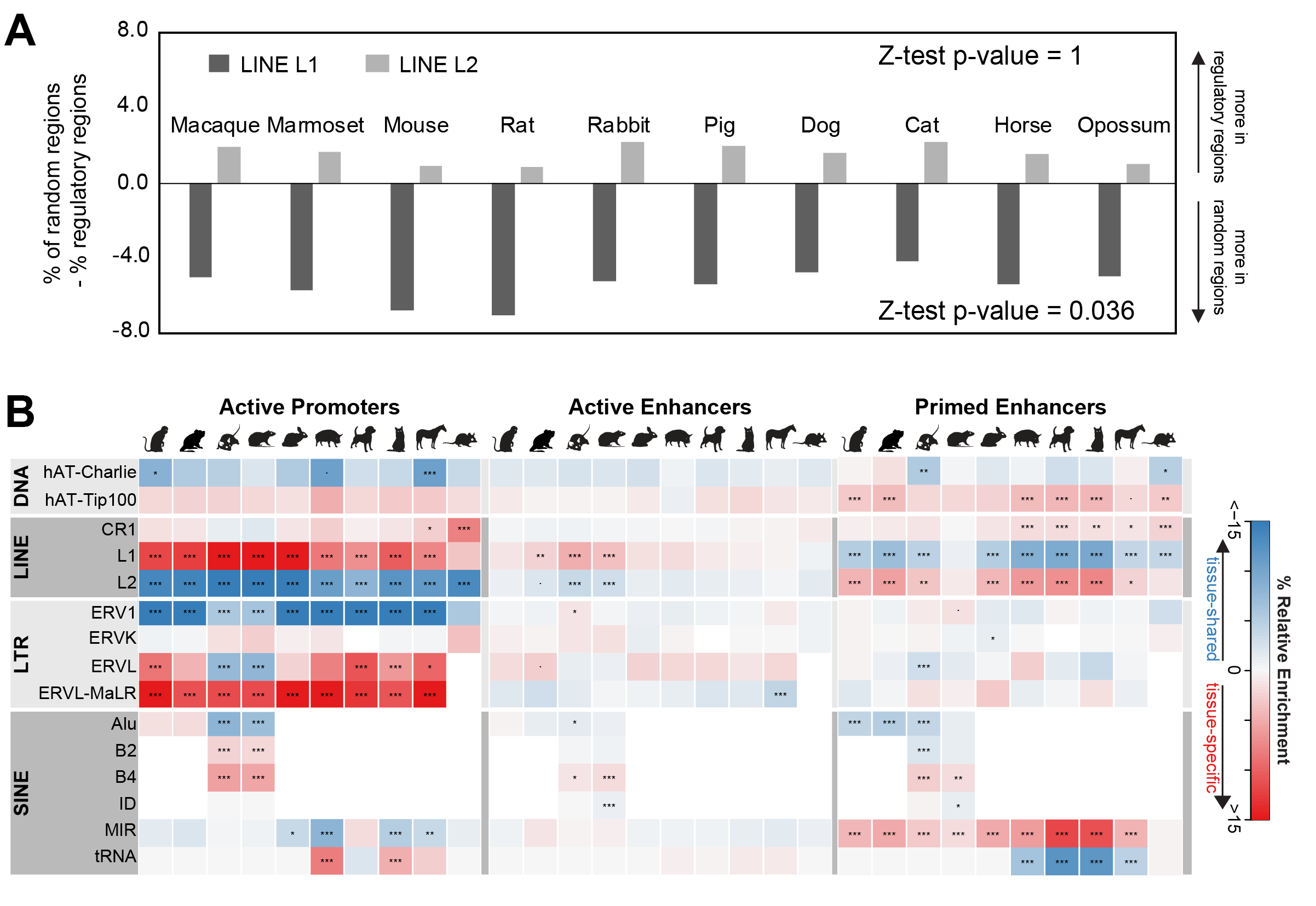


**Fig. S9: Genomic characteristics of LINEs.**

A) To compare the overlap of LINE L1s and L2s in regulatory regions to random overlap in the genome, within each species we randomly selected the same number and length of regions as was the total number of regulatory regions identified in this study (Figure 1C). We then overlapped the random regions and regulatorily active regions with LINE L1 and L2 transposable elements requiring at least 50% of LINE element length overlap. For each species, the figure shows the percent of all regulatory regions falling within LINEs minus the percent of all random regions falling within LINEs. The resulting numbers represent more than expected by random overlap when they are positive, and less than expected when they are negative. The difference between the random overlap and regulatory overlap was tested using the z-test for LINE L1s and L2s separately.

B) The relative enrichment of maintained tissue-shared versus tissue-specific regulatory regions for transposable element families is shown as a heatmap [red=enriched in tissue-specific; blue=enriched in tissue-shared; white = comparable contribution to tissue-specific and tissue-shared]. LINE L1s are enriched in tissue-specific regulatory regions, while LINE L2s are enriched in tissue-shared. Within each family, significance of tissue-specific vs. tissue-share proportions calculated with the Z-test and Bonferroni correction (P-values *** < 0.001; ** < 0.01; * < 0.05; - < 0.1). A similar heatmap for recently-evolved tissue-shared versus tissue-specific regulatory regions is shown in Figure 5A.


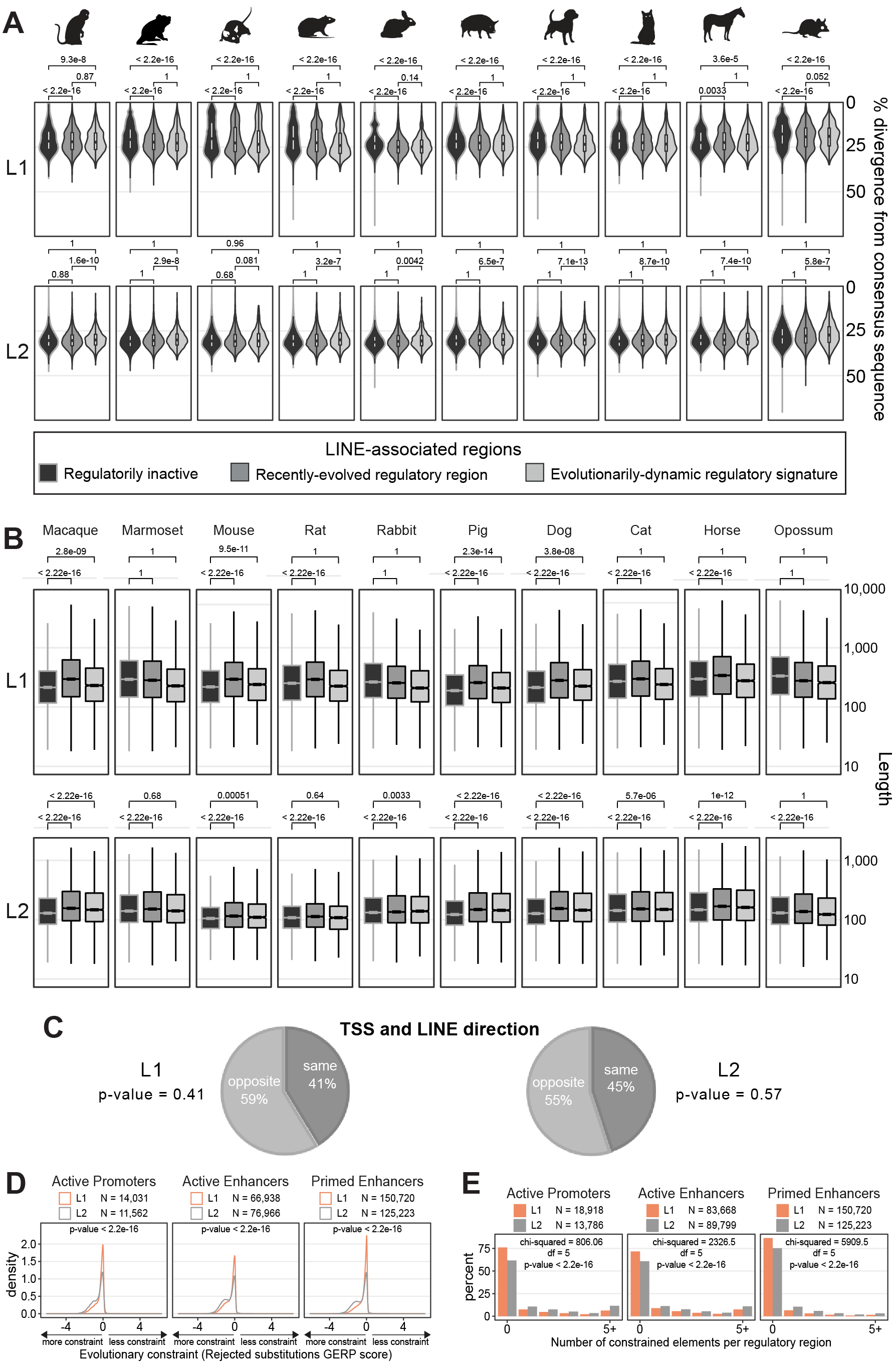


**Fig. S10: Regulatory active LINE L1s are not represented in the most recent whole genome expansions.** (Related to Figure 5).

A) For every regulatory region associated (medium and light greys) and inactive (dark grey) LINE L1 and L2 within each species, we calculated the number of mutations from consensus sequence as a proxy for transposable element age. Analysis is the same as for Figure 5D, which showed divergence for all ten species combined. p-values were calculated using one sided the Wilcoxon test for greater mean sequence divergence between all groups.

B) For every regulatory region associated (medium and light greys) and inactive (dark grey) LINE L1 and L2 within each species, we calculated the length of transposable elements. p-values were calculated using one sided the Wilcoxon test for greater length of regulatorily associated regions than inactive regions.

C) We grouped every regulatorily active LINE L1 and L2 that overlapped a known TSS across all species according to whether the TSS and LINE were on the same strand (same) or on different strands (opposite). We calculated the p-value using the t-test, testing the real distribution across all ten species compared to an expected random distribution (50% each category).

D) The distribution of GERP scores for alignable tissue-specific regulatory regions associated with LINE L1s and L2s. A negative GERP scores indicates more rejected substitutions than expected and is evidence of evolutionary selection. LINE L2s, even when tissue-specific in activity, are more constrained than their L1 associated tissue-specific counterparts. P-values calculated using the Wilcoxon test.

E) Distribution of the percent of tissue-specific regulatory regions associated with LINE L1s and L2s according to the total counts of constrained elements they contain, regardless of alignability. Tissue-specific LINE L2s less commonly have no constrained elements, and more commonly have 1 or more constrained elements than LINE L1s. The Chi-square test was performed on raw counts of total LINE L1 and L2 regions.

**SUPPLEMENTAL TABLES AND LEGENDS**

**Table S1: Species used in this study.**

| **Species (strain/breed)** | **Species scientific name** | **Assembly Version Ensembl (GenBank)** | **Age of sexual maturity / lifespan** | **Provider** | **Provider class** | **Num. of individuals used** | **Sex** | **Age** |
| --- | --- | --- | --- | --- | --- | --- | --- | --- |
| Rhesus Macaque | *Macaca mulatta* | Mmul_10 (GCA_003339765.3) | 4 years /  20 years | MRC Harwell Centre for Macaques (UK) | Research colony | 5 | All M | 5 - 21 years |
| Common Marmoset | *Callithrix jacchus* | ASM275486v1 (GCA_002754865.1) | 1.5 years /  12 years | Cambridge University (UK)  and Dstl (UK) | Research colony | 7 | 1 F  6 M | 1.5 - 19 years |
| Mouse (C57BL/6J) | *Mus musculus* | GRCm38.p6 (GCA_000001635.8) | 6-8 weeks /  1-3 years | Charles River (UK) | Commercial | 11 | 1 F  10 M | 9 - 14 weeks |
| Rat  (Brown Norway) | *Rattus norvegicus* | Rnor_6.0 (GCA_000001895.4) | 5 weeks /  1-3 years | Charles River (UK) | Commercial | 6 | All M | 10 weeks |
| Rabbit | *Oryctolagus cuniculus* | OryCun2.0 (GCA_000003625.1) | 5-6 months /  8-12 years | Envigo (UK) | Commercial | 6 | All M | 5 - 12 months |
| Pig  (Domestic) | *Sus scrofa* | Sscrofa11.1 (GCA_000003025.6) | 6 months /  10-15 years | Harlan ltd (UK) | Commercial | 3 | All M | 2 years |
| Dog  (Beagle) | *Canis familiaris* | CanFam3.1 (GCA_000002285.2) | 1 year /  12-15 years | Harlan ltd (UK) | Commercial | 4 | All M | 1 - 2.5 years |
| Cat | *Felis catus* | Felis_catus_9.0 (GCA_000181335.4) | 5-10 months /  15 years | Isoquimen ltd (Spain) | Commercial | 6 | 2 F  4 M | 1 - 2.5 years |
| Horse  (Welsh Mountain Pony) | *Eq­­uus caballus* | EquCab3.0 (GCA_002863925.1) | 12-15 months /  25-30 years | Animal Health Trust (UK) | Research colony | 4 | All M | 2 - 2.5 years |
| Grey Short-tailed Opossum | *Monodelphis domestica* | ASM229v1 (GCA_000002295.1) | 4-5 months /  4-8 years | Francis Crick Institute (UK) | Research colony | 5 | All M | 1 year |

Overview of the experimental samples and genomic versions used in the study, for more details see also **Table S2.**

**Table S4: Numbers and definitions of regulatory regions**

| **Peaks** | **Regulatory Region** | **Liver** | **Muscle** | **Brain** | **Testis** |
| --- | --- | --- | --- | --- | --- |
| H3K4me3 only | - | 3,011 | 5,595 | 6,183 | 86,551 |
| H3K27ac only | - | 84,991 | 82,290 | 128,536 | 164,347 |
| H3K4me1 only | Primed Enhancers | 504,838 | 477,974 | 513,496 | 528,369 |
| H3K4me1 + H3K27ac  (no H3K4me3) | Active Enhancers | 355,625 | 345,545 | 407,264 | 272,789 |
| H3K4me3 + H3K4me1  (no H3K27ac) | - | 3,802 | 6,874 | 5,072 | 19,540 |
| H3K4me3 + H3K27ac  (no H3K4me1) | Active Promoters | 65,688 | 61,745 | 80,845 | 94,771 |
| H3K4me3 + H3K27ac + H3K4me1 | Active Promoters | 111,902 | 118,936 | 120,461 | 145,839 |

Regulatory regions were defined within each tissue using the overlap between biologically reproducible peaks (see Methods). Active promoters were defined first as regions with H3K4me3 and H3K27ac peaks, and quite often also overlapped H3K4me1 peaks. Active enhancers were defined next as regions with H3K4me1 and H3K27ac peaks, but excluding and H3K4me3 peaks, and lastly primed enhancers were defined as regions with only H3K4me1 peaks. All other combinations of peak overlaps were not defined as regulatory regions (-).
