# Supplementary material for: LINE retrotransposons characterize mammalian tissue-specific and evolutionarily dynamic regulatory regions
Source: Genome Biol. 2021 Feb 18;22:62. doi: 10.1186/s13059-021-02260-y (PMC7890895; doi:10.1186/s13059-021-02260-y)
Supplement: Supplementary file 10 — Additional file 10. Review history. [file 13059_2021_2260_MOESM10_ESM.docx]

Review History

**First round of review**

**Reviewer 1**

**Are you able to assess all statistics in the manuscript, including the appropriateness of statistical tests used?**

Yes, and I have assessed the statistics in my report.

**Comments to author:**

The manuscript entitled "LINE elements are a reservoir of regulatory potential in mammalian genomes" by Roller et.al. describes a comprehensive analysis of active promoters and enhancers from 4 different organs collected from 10 different species representing several mammalian clades. The authors use RNA-Seq and CHIP-Seq approaches to identify these regulatory elements and explore their evolutionary relationships across different tissues and clades as well as between species of the same clades. Their CHIP-Seq approach is designed to distinguish between active and primed enhancers, which, in turn, allows them to perform a refined analysis of tissue- and species-specific evolutionary trends of promoter/enhancer transitions.

Among the multiple strengths of this manuscript are: (i) identification and analysis of functional regulatory elements in multiple organs (with biological replicates) from 10 species, (ii) ingroup and outgroup analyses, (iii) independent analysis of active and primed enhancers. The manuscript is clearly written, easy to follow, and provides important details of experimental procedures in the materials and methods section. Among novel findings are an in depth understanding on a much broader scale than ever before of the evolutionary ties between tissue-specific or tissues-shared promoters and enhancers as well as different types of enhancers and the contribution of repetitive elements, such as LINE L1 and L2, to functional promoters and enhancers. Overall, the findings are of broad interest to the scientific community and are expected to significantly improve our knowledge regarding regulatory landscapes important for normal tissue function and to set a baseline for identifying changes that maybe important during transition of these tissues to various disease states.

Some concerns that should be addressed:

1. With only a few exceptions, the results section does not contain clear wording on which differences were statistically significant and which were not, even though the authors regularly state that some values or frequencies were less/more than others (one of many such examples is p9, second paragraph). Even though this information is provided in figures, it would be helpful if it was also proved in the text to support all relevant statements with the names of performed tests and corresponding p values or by stating that the difference is statistically significant.

2. The observed variation between individual samples collected from the same species needs to be reflected in the manuscript to give the reader an appropriate understanding of the extent of differences within each species as well as between different groups. This would significantly improve the quality of the manuscript.

3. CHIP-Seq analysis was performed using Illumina sequencing to generate 50bp single reads. The alignment of these reads was done using default conditions. It is not clear whether L1s that overlap with promoters/enhancer regions were identified by this method or only by finding the overlap of boundaries between these two categories using genome annotation of LINEs. If any L1s were identified by alignment there needs to be some validation that these alignment parameters are appropriate for unambiguous identification of individual loci.

4. L1 and L2 loci were a part of regulatory elements if there was a 1 bp overlap in coordinates. It is not clear whether the orientation and the size (full-length or truncated) of LINE loci were considered as they may be important to understand whether the contribution to the evolution of regulatory elements is from the LINE promoter or other LINE sequences. As LINEs are abundant in most genomes, is the observed frequency any different from their random occurrence near regulatory elements? It is not clear whether this was tested.

5. The authors state that similar analysis between human and mouse regulatory elements was performed in the past. It would be helpful to clearly justify why human tissues were not included into this study.

6. P7, 2nd paragraph (we investigated the association between promoters and enhancers by assigning enhancers to the nearest promoter …"). This is a rather linear approach given that an enhancers' action is not distance dependent. This approach needs to be justified. Would it be possible to use available (if any) 3D analysis of any of the organs used in this study to understand how it may/would change the outcome?

Minor suggestions/comments:

1. The authors discuss the strengths and weaknesses of their approaches in the discussion. It would be helpful to expand this discussion to provide better understanding of how the results could be influenced if different assumptions were made.

2. LINE is Long INterspersed Element, not Long Interspersed Nuclear Element same is for SINEs

3. P4 a citation is listed in a different format - as a name not a number

4. Citation Deininger et.al., NAR 2017 should be added to the 31-33 citations

5. The title only reflects one of the findings in the manuscript and could be broadened

6. It is more appropriate to refer to liver, testis, etc. as organs instead of tissues

**Reviewer 2**

**Are you able to assess all statistics in the manuscript, including the appropriateness of statistical tests used?**

Yes, and I have assessed the statistics in my report.

**Comments to author:**

This paper surveyed on the role of Long Interspersed Nuclear Elements (LINEs) elements, L1 and L2, in the evolvement of tissue-shared and tissue-specific regulatory regions (promoters and enhancers) across mammalian genomes. Specifically, the generated dataset spanned ChIP-seq data (H3K4me3, H3K27ac, and H3K4me1) and RNA-seq data of 4 tissues (liver, muscle, brain, and testis) from 10 mammals. A total of 2.8 million regulatory regions were defined in four adult tissues across 10 species. The authors elucidated that the genomic regions switching between promoters and enhancers were more common across species, and less common across tissues within a single species. LINE L1s showed association with tissue-specific regulatory regions, while LINE L2s showed association with tissue-shared regulatory regions and those evolutionarily "dynamic" regions with signatures switching between promoter and enhancer across species.

As a whole, it's an impressive study with nearly 500 matched experiments. I particularly liked the fact that the authors were able to look at the association between tissue specificity and evolutionary stability for their enhancers and promoters regions.

Overall, I really liked the study but have a number of major and minor comments. In particular, I had some concerns around the choices of thresholds and their impact on results and so suggested some additional analyses (points 5-7). I also had some concerns on the final results on LINEs that could have used some additional controls or analyses (point 9).

Major comments:

1)	The authors emphasize in the introduction section that some genomic regions can show both promoter and enhancer activities. I think it would be helpful if the authors could summarize the peaks detected across species into a supplementary figure or table, showing how many genomic regions are overlapped with all three types of peaks, etc.

2)	In Fig 1C, could some of the variability you observe between species be attributed to genome size?

3)	In Fig 2C, what are the lines in the figure? A regression? I couldn't get it from the caption. Overall, I found this figure a bit complicated to read and I wonder if you could get the same point across with a simpler figure (while still showing this one in the supplements perhaps)? Maybe by showing the distribution of ratios shared/specific?

4)	Difference between testis and somatic tissue. There were several observations showing difference between testis and somatic tissue (such as Figure 2D and Figure 3C). The distinction was obviously seen. However, has the higher threshold of minimum reads set for testis than other tissues been ruled out from contributing to the discrepancy between testis and somatic tissues? Was the fold enrichment for identified enhancers in testis higher than that of somatic tissues? Perhaps comparing the testis-version and somatic-tissue version of Figure S2D would be informative regarding this concern?

5)	Fig 3B but this comment also applies to other sections of the manuscript. Do you potentially have an ascertainment bias? Promoters are detected by the presence of 3 marks, active enhancers by 2 marks and poised enhancers by 1 mark. Because of this, I would assume you are likely to have weaker, noisier, signal in your poised enhancers. If you were to partition your poised enhancers in 4 quartiles (from strongest to weakest) and did the same with your active enhancers, would you still see such a strong separation, or would the "weaker" active enhancers overlap the "stronger" poised enhancers?

6)	Related to that, the identification of promoters and enhancers is the foundation of this paper. The identification of enhancers is typically more challenging than promoters and is worth discussion. In Figure S2D, specifically for the Enhancers subpanel on the right, some regions (specifically in the "ensembl and current" subset) were classified as enhancers when all three histone marks showed similar levels of fold enrichment. Two concerns arise here. (1) The majority of classified enhancers indeed showed enrichment of H3K27ac and H3K4me1 but not H3K4me3 as expected. However, the top of the "ensembl and current" portion of the bottom panel for enhancers contained some regions clearly showed high read density of all three histone marks. Shouldn't these regions with enrichment of all three histone marks classified as promoters based on the defined criteria? (2) The average fold enrichment (top panel in Figure S2D) for enhancers was about 1/10 of that of promoters. Would this discrepancy introduce a lower confidence level for the estimation of enhancers than that of active promoters?

7)	Figure 4 and associated sections. A similar comment applies here. You're converting quantitative signals of histone marks into specific discrete calls (promoters, active enhancers, poised enhancers). How much of what you present here is associated with thresholds effect versus truly dynamic elements? You should expand this section with an analysis of threshold effects. I would still expect (and be suspicious) if your dynamic calls are systematically weaker. One approach you may be able to use to estimate this is the amount of inconsistencies you find on the evolutionary tree for your calls.

8)	When analyzing the promoter and enhancer signature switches between species. The authors considered all switches between species in any tissues (Figure S3A). As the authors showed in the Figure 4H, Figure 2B, and Figure S3B, almost all the promoters and enhancers were tissue-specific, especially for enhancers, including the dynamic regulatory regions as well (Figure 4H). I am wondering if the tissue-specific enhancers dominate the evolutionary dynamic regulatory signatures. I would suggest to do another analysis considering the same tissues only to achieve similar trend.

9)	Figure 5A and the results on LINEs. How do you know that some of this is not an effect of: 1) poorer genome assembly in those regions in some of the species and 2) mapping problems in those regions (given that mapping of short reads in LINEs in particular can be challenging)? As additional confirmation that these effects are real, were you able to identify motifs enriched in these repetitive regions confirming/explaining this activation?

10)	The current TE enrichment analysis is based on the number of regulatory genomic regions that overlap with a minimum of one base with TEs. I think this threashold is potentially too low. Have you tried the peak summits that overlapped with TEs? Or genomic regions that overlap with 50% of TEs as you use to compare between peaks?

Minor comments:

Page 5, line 23 - 24: "more than 2.8 million regulatory regions were annotated". It would be better to specify is it consensus genomic regions, or the number of each tissue and each species were added up.

Page 6, line 1: "thousands of novel regulatory regions were found". I am curious is it due to more species you included or is it mainly because of multiple samples per species were used, or more tissues were included.

Page 6, Line 4: "The number of regulatory regions", is it refer to the average number of regulatory regions per sample, or the total number of consensus genomic regions.

Page 6, line 13, it can be specified to be "primate" brain regions here.

Page 7, first paragraph. It would be clearer if a pie chart or bar chart can be added in Figure 2B to show the percentage as introduced in this paragraph.

Page 7, line 13, regarding this conclusion "tissue-specific promoters modulate alternative transcript usage". It would be more convincing if you can truly detect the alternative transcripts from the RNA-seq data compare to the use of the transcripts and genes counts only.

Page 8, line 7, active enhancers also closely associated with tissue-specific gene expression?

Page 8, line 16: "maintained in evolution" maybe specify in this group of species

Page 13, line 6, "promoters arise from enhancers six times more often than enhancers arise from promoters". Is this conclusion based on the analysis of species with shorter evolutionary distances by using the house and rabbit as outgroup? Will it be true as well if you include the species with longer evolutionary distances?

Figure 1: C panel, the x-axis is confused. Is it proportion, what does it refer to? Is it stack bars? D panel, can be improved by using the confident interval instead of mean value only.

Figure 4: panel B, it shows the proportion of each regulatory signature can be mapped to others. The authors point out that promoter and enhancer signature switching is more common between species, but with no real data here for comparison. Is it possible to also include a figure like panel A as a supplementary figure? We can easily check the total number of dynamic P/Es, and Es, which I have not found somewhere yet.

Figure 5, we observed more consistently relative enrichment compare between tissue-shared and tissue-specific active promoters and active enhancers in L1 and L2, but it is not consistent between switch and stable genomic signatures for active promoters. Are there any better explanations for it? It would be better to include the counts of each TE family overlap with each regulatory signature as a supplementary table.

FigureS2C, numbers shown in the figure seemed inconsistent with that described in the legend.

FigureS2C, perhaps modify the legend title from "Classification of overlapping regulatory region" to "Classification of overlapping regulatory region in current study" (check if this is correct) to increase clarity.

Figure S2D, it would be helpful to clarify in the caption about what each row represented for the bottom panel.

Figure 5E appeared before Figure 5D in the main text.

**Authors Response**

**Point-by-point responses to the reviewers’ comments:**

Reviewer #1: ===

The manuscript entitled "LINE elements are a reservoir of regulatory potential in mammalian genomes" by Roller et.al. describes a comprehensive analysis of active promoters and enhancers from 4 different organs collected from 10 different species representing several mammalian clades. The authors use RNA-Seq and CHIP-Seq approaches to identify these regulatory elements and explore their evolutionary relationships across different tissues and clades as well as between species of the same clades. Their CHIP-Seq approach is designed to distinguish between active and primed enhancers, which, in turn, allows them to perform a refined analysis of tissue- and species-specific evolutionary trends of promoter/enhancer transitions.

Among the multiple strengths of this manuscript are: (i) identification and analysis of functional regulatory elements in multiple organs (with biological replicates) from 10 species, (ii) ingroup and outgroup analyses, (iii) independent analysis of active and primed enhancers. The manuscript is clearly written, easy to follow, and provides important details of experimental procedures in the materials and methods section. Among novel findings are an in depth understanding on a much broader scale than ever before of the evolutionary ties between tissue-specific or tissues-shared promoters and enhancers as well as different types of enhancers and the contribution of repetitive elements, such as LINE L1 and L2, to functional promoters and enhancers. Overall, the findings are of broad interest to the scientific community and are expected to significantly improve our knowledge regarding regulatory landscapes important for normal tissue function and to set a baseline for identifying changes that maybe important during transition of these tissues to various disease states.

*We thank the reviewer for recognising the contributions of our manuscript and impact of the results, as well as helping to improve our analyses and their presentation.*

Some concerns that should be addressed:

1. With only a few exceptions, the results section does not contain clear wording on which differences were statistically significant and which were not, even though the authors regularly state that some values or frequencies were less/more than others (one of many such examples is p9, second paragraph). Even though this information is provided in figures, it would be helpful if it was also proved in the text to support all relevant statements with the names of performed tests and corresponding p values or by stating that the difference is statistically significant.

*We agree with the reviewer and have now added p-values to the text when significance is mentioned.*

2. The observed variation between individual samples collected from the same species needs to be reflected in the manuscript to give the reader an appropriate understanding of the extent of differences within each species as well as between different groups. This would significantly improve the quality of the manuscript.

*We thank the reviewer for this suggestion. We have now added a supplementary Figure S2B which shows the variability between individual replicates for all species and tissues and refer to it in the Results page 5. This analysis shows that H3K4me3 and H3K27ac peaks are mostly highly reproducible between replicates, and while there is slightly more variation in total peak counts between replicates for H3K4me1 most peaks are still found in at least two replicates.*

3. CHIP-Seq analysis was performed using Illumina sequencing to generate 50bp single reads. The alignment of these reads was done using default conditions. It is not clear whether L1s that overlap with promoters/enhancer regions were identified by this method or only by finding the overlap of boundaries between these two categories using genome annotation of LINEs. If any L1s were identified by alignment there needs to be some validation that these alignment parameters are appropriate for unambiguous identification of individual loci.

*The initial mapping was done using default parameters, but we later used a number of quality filters including eliminating all alignments below MAPQ 1. Though this does usually eliminate most multimapping reads, we agree with the reviewer that additional checks for unambiguous read alignments would benefit the manuscript. We have now partitioned the mapped quality filtered reads into those that do not map to repeats, those mapping in LINE L1s and mapping in LINE L2s (Table S8 Results page 16) and checked how many of them have unique alignments in all genomes (Methods). We found that our quality filters are appropriate for unambiguous alignments across LINEs and comparable to those reads aligning to non-repetitive parts of the genome. Namely, on average 1% of our reads mapped in the non-repetitive genome are multimappers, compared to around 3% for L2s and 4% for L1s. This shows that a minimum of 96% of all quality passed reads that we mapped to LINEs are mapping unambiguously, probably to the more diverged parts of the transposable sequence and is in line with our finding that more ancient LINEs contribute to regulatory landscapes.*

4. L1 and L2 loci were a part of regulatory elements if there was a 1 bp overlap in coordinates. It is not clear whether the orientation and the size (full-length or truncated) of LINE loci were considered as they may be important to understand whether the contribution to the evolution of regulatory elements is from the LINE promoter or other LINE sequences. As LINEs are abundant in most genomes, is the observed frequency any different from their random occurrence near regulatory elements? It is not clear whether this was tested.

*We thank the reviewer for this suggestion, we have now compared LINE occurrence in regulatory regions compared to random genomic regions in Figure S10B, and mention these in Results page 16 and Discussion page 20. This analysis helps underline that LINE occurrence in regulatory regions is not drastically different from the whole genome, and that the only way to see differing contributions of LINEs to tissue-specific, tissue-shared or evolutionarily dynamic regions is to carefully compare one of the groups to the other. If we were only looking for those transposable elements that are in regulatory regions a lot more than random, we might have missed these trends.*

*Additionally, we have also checked the orientation of LINEs with regards to regulatory regions identified in the study. Given that ChIP-seq histone peaks do not have directionality, we limited our analyses to LINEs overlapping transcription start sites (Figure S10C, discussion Results page 18), and find that the directionality is random. This means that the LINEs are not likely to be contributing their own promoters or TSSs to new regulatory regions. The additional analyses performed, together with the previous results of the age of LINEs (Figure 5D, S10B) help underline that most young LINEs do not have regulatory signature in the current study, but that additional divergence might necessary for regulatory functions.*

*According to advice offered by both reviewers, we have now also increased LINE elements overlap to be of half the transposable segment length for Figures 5A, 5E and S9B.*

5. The authors state that similar analysis between human and mouse regulatory elements was performed in the past. It would be helpful to clearly justify why human tissues were not included into this study.

*We agree that it would be very interesting to have performed the same experiments in human. However, acquiring matching healthy human tissues using similar or identical collection procedures to ensure sufficient quality and large enough amounts is extremely difficult especially for the testis and muscle. Thus, in order to prepare all tissues in controlled conditions and avoid batch effects that could bias the analyses, we chose to only perform the study on tissues from species that we had personally acquired.*

6. P7, 2nd paragraph (we investigated the association between promoters and enhancers by assigning enhancers to the nearest promoter …"). This is a rather linear approach given that an enhancers' action is not distance dependent. This approach needs to be justified. Would it be possible to use available (if any) 3D analysis of any of the organs used in this study to understand how it may/would change the outcome?

*To the best of our knowledge there is no available matched 3D genome data, such as capture HiC, at the resolution necessary to connect promoters to enhancers. However, studies have shown that around 70% of enhancers do act on their nearest gene/promoter (references 52 and 53). We have now clarified this in Results page 8 and comment on the limitations in Discussion page 19.*

Minor suggestions/comments:

1. The authors discuss the strengths and weaknesses of their approaches in the discussion. It would be helpful to expand this discussion to provide better understanding of how the results could be influenced if different assumptions were made.

*We have now added in the Discussion statements about how the choice of different confidence intervals for calling promoters would have impacted our results (see also answer to Reviewer 2). We have also added in the Discussion how looking for only those transposable elements that are differently associated to regulatory regions than random regions would have not revealed the trends we have seen (see also point 4 of these comments).*

2. LINE is Long INterspersed Element, not Long Interspersed Nuclear Element same is for SINEs

*We recognise that both acronym expansions are in use. We believe that the one we used, which is used both by Wikipedia and the recent review by Bourque, et al, Genome Biology 2018 "Ten thing you should know about transposable elements" is in more common usage. We have added this additional expansion as an alternative in the main text where the acronym is first defined.*

3. P4 a citation is listed in a different format - as a name not a number

*Thank you, the reference is now properly formatted.*

4. Citation Deininger et.al., NAR 2017 should be added to the 31-33 citations

*This citation has now been added as citation 35.*

5. The title only reflects one of the findings in the manuscript and could be broadened

*We thank the reviewer for this suggestion and have now broadened the title.*

6. It is more appropriate to refer to liver, testis, etc. as organs instead of tissues

*We agree that liver, testis, brain and muscle are technically organs, however it is common in the field to refer to them as “tissues” to reflect that usually only parts, i.e. tissue samples, of the entire organs are used for experiments. We have clarified this in the Results section page 5.*

===

Reviewer #2: This paper surveyed on the role of Long Interspersed Nuclear Elements (LINEs) elements, L1 and L2, in the evolvement of tissue-shared and tissue-specific regulatory regions (promoters and enhancers) across mammalian genomes. Specifically, the generated dataset spanned ChIP-seq data (H3K4me3, H3K27ac, and H3K4me1) and RNA-seq data of 4 tissues (liver, muscle, brain, and testis) from 10 mammals. A total of 2.8 million regulatory regions were defined in four adult tissues across 10 species. The authors elucidated that the genomic regions switching between promoters and enhancers were more common across species, and less common across tissues within a single species. LINE L1s showed association with tissue-specific regulatory regions, while LINE L2s showed association with tissue-shared regulatory regions and those evolutionarily "dynamic" regions with signatures switching between promoter and enhancer across species.

As a whole, it's an impressive study with nearly 500 matched experiments. I particularly liked the fact that the authors were able to look at the association between tissue specificity and evolutionary stability for their enhancers and promoters regions.

Overall, I really liked the study but have a number of major and minor comments. In particular, I had some concerns around the choices of thresholds and their impact on results and so suggested some additional analyses (points 5-7). I also had some concerns on the final results on LINEs that could have used some additional controls or analyses (point 9).

*We thank the reviewer for recognising the scope of the study and helpful comments on additional clarifications and analyses.*

Major comments:

1) The authors emphasize in the introduction section that some genomic regions can show both promoter and enhancer activities. I think it would be helpful if the authors could summarize the peaks detected across species into a supplementary figure or table, showing how many genomic regions are overlapped with all three types of peaks, etc.

*We thank the reviewer for this suggestion. We have now provided Table S4 which summarises all possible histone peak combinations, their numbers, and the resulting regulatory region assignments. The figure is referenced in the Results section on page 5.*

2) In Fig 1C, could some of the variability you observe between species be attributed to genome size?

*We have added the size of the golden path of the underlying assemblies to Figure 1C to show that most eutherian mammals do not have drastically different genome sizes. For example, rabbit and mouse have very similar assembly sizes but different numbers of peaks called. Different total peak calls are more likely to be a technical effect, either in the efficiency of the experimental protocols, different tissue collection time points, or the quality of the underlying genome assembly.*

3) In Fig 2C, what are the lines in the figure? A regression? I couldn't get it from the caption. Overall, I found this figure a bit complicated to read and I wonder if you could get the same point across with a simpler figure (while still showing this one in the supplements perhaps)? Maybe by showing the distribution of ratios shared/specific?

*We have now clarified in the legend of Figure 2C that the line is indeed a regression fitted to the distribution of the number of tissue-specific and tissue-shared enhancers per promoter within each graph facet. We have also added a supplementary Figure S6B which shows the same data as ratios, as per the reviewer’s suggestion.*

4) Difference between testis and somatic tissue. There were several observations showing difference between testis and somatic tissue (such as Figure 2D and Figure 3C). The distinction was obviously seen. However, has the higher threshold of minimum reads set for testis than other tissues been ruled out from contributing to the discrepancy between testis and somatic tissues? Was the fold enrichment for identified enhancers in testis higher than that of somatic tissues? Perhaps comparing the testis-version and somatic-tissue version of Figure S2D would be informative regarding this concern?

*Our previous incomplete description of the experimental data has likely led to confusion. We did not use a different number of reads for ChIP-seq in testis. We use the same number of ChIP-seq reads for all tissue with differences for the histone modification based on peak saturation analyses (Figure S2A; 20 million for H3K4me3; 20 million for H3K27ac; and 40 million for H3K4me1). We did use a different number of reads in testis for the RNA-seq data. We have now clarified this in the manuscript.*

*We have also added to the results section an analysis of ChIP-seq read thresholds (Results page 9), as well as made a dedicated figure S4B to focus on signal saturation across all tissues and histone modifications. Figure S4B and the related discussion in Results page 7 now illustrates that, though fold enrichment for H3K4me3 is lower in the testis due to more peaks being called compared to somatic tissues, the fold enrichment for H3K27ac is comparable across the somatic tissues and testis. Considering that we required enrichment of both H3K4me3 and H3K27ac to call promoters, we believe that it is extremely unlikely that the lower enrichment of H3K4me3 alone can be responsible for the faster evolutionary turnover or greater tissue-specificity in the testis.*

5) Fig 3B but this comment also applies to other sections of the manuscript. Do you potentially have an ascertainment bias? Promoters are detected by the presence of 3 marks, active enhancers by 2 marks and poised enhancers by 1 mark. Because of this, I would assume you are likely to have weaker, noisier, signal in your poised enhancers. If you were to partition your poised enhancers in 4 quartiles (from strongest to weakest) and did the same with your active enhancers, would you still see such a strong separation, or would the "weaker" active enhancers overlap the "stronger" poised enhancers?

*We thank the reviewer for suggesting this additional control analysis. In the most basic interpretation of our work, in this study we measure the evolution of histone marks across species while the categorisations as types of regulatory regions is an interpretation of those results. Though promoters can have an overlapping H3K4me1 peak and are quite often flanked by H3K4me1 (Figure S3C), we did not require this when calling active promoters and only around 2/3 of promoters called did have a H3K4me1 peak (Table S4). To create a set that is more comparable between the different regulatory regions, we selected the subset of regions that have comparable q-values as computed by MACS2 from the difference in ChIP-seq and input signal in each library. To make active promoters and enhancers comparable we used H3K27ac, which selected for the strongest active enhancers and weakest active promoters on the mark they share. Similarly, to make the active and primed enhancers comparable we used H3K4me1, which selected for the strongest primed enhancers and weakest active enhancers (Figure S2C). Using the above defined regions we repeated the analysis in Figure 3B (Figure S7A and Result text page 9), and show that the same evolutionary trends are still observable as when using the whole regulatory regions set, even when controlling for signal strength.*

6) Related to that, the identification of promoters and enhancers is the foundation of this paper. The identification of enhancers is typically more challenging than promoters and is worth discussion. In Figure S2D, specifically for the Enhancers subpanel on the right, some regions (specifically in the "ensembl and current" subset) were classified as enhancers when all three histone marks showed similar levels of fold enrichment. Two concerns arise here. (1) The majority of classified enhancers indeed showed enrichment of H3K27ac and H3K4me1 but not H3K4me3 as expected. However, the top of the "ensembl and current" portion of the bottom panel for enhancers contained some regions clearly showed high read density of all three histone marks. Shouldn't these regions with enrichment of all three histone marks classified as promoters based on the defined criteria?

*We thank the reviewer for careful inspection of histone enrichment graphs presented in old figure S2D, S3C in the current manuscript version. The high enrichment of H3K4me3 in a small number of enhancers was a consequence of performing the underlying histone enrichment analyses using averages across all tissues and was capturing those regions we defined later in the manuscript as “inter-species dynamic regions”. Additionally, the analysis was structured so that inter-species dynamic region would have been shown both in the “Promoter” and the “Enhancer” plots. Consequently, the enhancer histone enrichment graphs were capturing the high H3K4me3 enrichment which classified them as promoters in one tissue, despite there not being enough H3K4me3 enrichment in another tissue for promoter classification.*

*We have now performed the signal enrichment plots within each tissue, instead of as average across all tissues, and provide these graphs in the new Figure S3C. Specifically, we also now show the read enrichment across all regulatory regions and tissues to the same scale for each histone mark to ease comparisons. The graphs show that the H3K4me3 signal in regulatory regions called as enhancers is extremely low within the tissue where it is an enhancer, and consequently insufficient to call a peak of a q-value threshold of 0.05 using MACS2 (see Methods and Scripts). Without averaging across tissues, the H3K4me1 signal is now comparably enriched between promoters and enhancers. Given that Ensembl and RefSeq do not make the distinction between active and primed enhancers, the H3K27ac enrichment is shown in this validation figure across both active enhancers (defined as having a H3K4me1 and H3K27ac peak) and primed enhancers (defined as having an H3K4me1 peak only) but is still around 1/5 in all enhancers compared to promoters. The higher H3K27ac read enrichment in promoters is not surprising given that they are less likely to be variable across individuals or cells (Figure S2B), which we incorporated into our subsequent evolutionary analyses by computing a separate 0 years divergence point for promoters and enhancers (Figure 3B, Figure S7A), and for each tissue separately where needed (Figure 3C, Methods).*

(2) The average fold enrichment (top panel in Figure S2D) for enhancers was about 1/10 of that of promoters. Would this discrepancy introduce a lower confidence level for the estimation of enhancers than that of active promoters?

*To further investigate read threshold and the confidence of peak calls across different regulatory regions called in our study, we compared the q-values of the underlying peaks called in Figure S2C and discuss these in Results page 5. The q-values of H3K27ac peaks within active promoter are higher than those in active enhancers, and similarly H3K4me1 q-values are higher in active compared to primed enhancers. However, there is significant overlap between those distributions that we used for further investigations of the effect on evolutionary rates (see above in Comment 5).*

7) Figure 4 and associated sections. A similar comment applies here. You're converting quantitative signals of histone marks into specific discrete calls (promoters, active enhancers, poised enhancers). How much of what you present here is associated with thresholds effect versus truly dynamic elements? You should expand this section with an analysis of threshold effects. I would still expect (and be suspicious) if your dynamic calls are systematically weaker. One approach you may be able to use to estimate this is the amount of inconsistencies you find on the evolutionary tree for your calls.

*We have now included an analysis of signal strength of underlying histone peaks used to call evolutionarily dynamic regions and compared them to those regions that are stable between species in Figure S8A (similar to analyses in Comment 5 and 7), which we discuss on Results page 12 and Discussion page 20. Though active promoters that are evolutionarily dynamic are indeed called using peaks with lower confidence levels than stable promoters, the confidence levels for the active enhancers overlap between evolutionarily dynamic and stable regions, and for primed enhancers evolutionarily dynamic peaks are in fact called with more confident peaks than for stable counterparts. We believe that this means that threshold effects are not sufficient to explain the evolutionary dynamics that we see, especially for enhancers which make up a larger portion of the dynamic regions (Figure 4B, S8B).*

8) When analyzing the promoter and enhancer signature switches between species. The authors considered all switches between species in any tissues (Figure S3A). As the authors showed in the Figure 4H, Figure 2B, and Figure S3B, almost all the promoters and enhancers were tissue-specific, especially for enhancers, including the dynamic regulatory regions as well (Figure 4H). I am wondering if the tissue-specific enhancers dominate the evolutionary dynamic regulatory signatures. I would suggest to do another analysis considering the same tissues only to achieve similar trend.

*The extent of the contribution of tissue-specific enhancers to dynamic regulatory signatures is indeed a very interesting question. We have now subsetted the analysis of chromatin signature dynamics which allowed all switches between any tissues (Figure 4B) so that we only consider those regulatory regions active in the same tissue across species, i.e. separately for liver, muscle, brain and testis (Figure S8B), and included the Results on manuscript page 12 of the manuscript. Even though a smaller number of active and primed enhancers are involved in evolutionary dynamic mappings within the same tissue, on average 52% still do map to another signature.*

9) Figure 5A and the results on LINEs. How do you know that some of this is not an effect of: 1) poorer genome assembly in those regions in some of the species and 2) mapping problems in those regions (given that mapping of short reads in LINEs in particular can be challenging)? As additional confirmation that these effects are real, were you able to identify motifs enriched in these repetitive regions confirming/explaining this activation?

*We have now added additional analyses and clarification around LINE assembly and mapping issues. All the species chosen for this study have relatively well assembled genomes overall, approaching to the quality of the most well assembled genome in the set – the mouse genome. This is most clearly evidence by an average of 98% of reads across all mouse ChIP-seq libraries having an alignment, compared to 96% in all other species (Table S3, Results page 16). Given that all of the LINE transposable elements we see as regulatorily active are not particularly young (Figure 5D), it is not surprising that we are able to unambiguously map reads within them almost as efficiently as within the non-repetitive part of the genome (Table S8, Results page 16, see also Reviewer 1 comment 3).*

*In differential analyses of LINE L1s and L2s we were not able to identify motifs that would explain the differences in regulatory activity observed. We agree with the reviewer that finding such motifs would help confirm their regulatory identity, but do not find it surprising that we could not identify them. The differences we see are in 1) tissue-specific vs. tissue-shared activation and 2) in activation as different regulatory signatures. In the first instance for activation in a tissue-specific or a tissue-shared manner, the within-tissue activation requires assembly of the same transcriptional activity regardless of tissue-specificity. For example, both a liver-specific promoter and a ubiquitous promoter would require binding of liver-specific transcription factors to be expressed in liver. Similarly, in the second instance of regulatory signature changes, active promoters, active enhancers and primed enhancers all share a set of transcription factors need for their activity. We have now included a discussion on motifs in Discussion page 20.*

10) The current TE enrichment analysis is based on the number of regulatory genomic regions that overlap with a minimum of one base with TEs. I think this threashold is potentially too low. Have you tried the peak summits that overlapped with TEs? Or genomic regions that overlap with 50% of TEs as you use to compare between peaks?

*Given that most genomic LINEs are truncated (Figure S10B) and old insertions (Figure 5D and S10A), be they regulatorily active or not, they are significantly shorter than regulatory regions called by ChIP-seq experiments. This makes the requirement that the regulatory regions we called be covered 50% with LINEs too restrictive. We do agree with the reviewer that 1 bp overlap might be too relaxed. Therefore we have now required for all the heatmaps analysing differential overlap of regulatory regions to have 50% of the transposable element covered with a regulatory region. Figures 5A, 5E and S9B have been replaced.*

Minor comments:

Page 5, line 23 - 24: "more than 2.8 million regulatory regions were annotated". It would be better to specify is it consensus genomic regions, or the number of each tissue and each species were added up.

*We have now clarified that this is a summation. Now on page 6, lines 7-8.*

Page 6, line 1: "thousands of novel regulatory regions were found". I am curious is it due to more species you included or is it mainly because of multiple samples per species were used, or more tissues were included.

*We have now added clarification on page 6.*

Page 6, Line 4: "The number of regulatory regions", is it refer to the average number of regulatory regions per sample, or the total number of consensus genomic regions.

*We have clarified on page 6 line 15 that this refers to consensus peaks, and directed the reader to schematic outline of the pipeline in Figure S1.*

Page 6, line 13, it can be specified to be "primate" brain regions here.

*Primate has been added to current page 6 line 25.*

Page 7, first paragraph. It would be clearer if a pie chart or bar chart can be added in Figure 2B to show the percentage as introduced in this paragraph.

*We have now added pie charts to Figure 2B and Figure S6A.*

Page 7, line 13, regarding this conclusion "tissue-specific promoters modulate alternative transcript usage". It would be more convincing if you can truly detect the alternative transcripts from the RNA-seq data compare to the use of the transcripts and genes counts only.

*We agree that this would be more convincing, but short-read RNA-seq data alone, such as that generated in this study, is not well suited to discovering new full length transcripts.*

Page 8, line 7, active enhancers also closely associated with tissue-specific gene expression?

*It might not have been clear enough that we were referring to the total numbers of tissue-specific active promoters and tissue-specific active genes, we have now clarified on page 9, line 2-3.*

Page 8, line 16: "maintained in evolution" maybe specify in this group of species

*This has been clarified on page 9.*

Page 13, line 6, "promoters arise from enhancers six times more often than enhancers arise from promoters". Is this conclusion based on the analysis of species with shorter evolutionary distances by using the house and rabbit as outgroup? Will it be true as well if you include the species with longer evolutionary distances?

*We have now clarified that the statement refers only to the evolutionary distances examined in Results page 14.*

Figure 1: C panel, the x-axis is confused. Is it proportion, what does it refer to? Is it stack bars? D panel, can be improved by using the confident interval instead of mean value only.

*We have now added additional explanation to the figure legend and hope it is clearer.*

Figure 4: panel B, it shows the proportion of each regulatory signature can be mapped to others. The authors point out that promoter and enhancer signature switching is more common between species, but with no real data here for comparison. Is it possible to also include a figure like panel A as a supplementary figure? We can easily check the total number of dynamic P/Es, and Es, which I have not found somewhere yet.

*Figure 4A shows the overlap of regulatory signatures within a species, which we named “intra-species” dynamic, and is a much easier concept to apply. For regulatory signatures across species, named “evolutionarily dynamic”, the dynamic assignment is more complicated and would require an arbitrary threshold. For example, if a region is an active promoter in 5 species and active enhancer in 1, should this be a “dynamic” or “stable” region? How would this compare to a region that is an active promoter in 3 species and an active enhancer in 3 others? Furthermore, as panel C shows, evolutionary distance increases the number of regions that switch regulatory signature so the number of “dynamic” calls made at large evolutionary distances could appear artificially high. For the reasons above, instead of a unified call across all we based all of our analyses in panel B and C on all possible pairwise comparisons between species.*

Figure 5, we observed more consistently relative enrichment compare between tissue-shared and tissue-specific active promoters and active enhancers in L1 and L2, but it is not consistent between switch and stable genomic signatures for active promoters. Are there any better explanations for it? It would be better to include the counts of each TE family overlap with each regulatory signature as a supplementary table.

*We have now included TE overlap as a table for recently-evolved regions in table S6 and maintained in S7. For the evolutionarily dynamic regions, we were limited to discovering only those regions that switch signatures between the species and tissues investigated – which is in no way comprehensive. We believe that this might weaken our ability to discover LINE associations as a wider sampling would reveal that regions with seemingly stable regulatory signature are indeed dynamic. We now discuss the limitations of identify evolutionary dynamic regions in the Discussion page 20.*

FigureS2C, numbers shown in the figure seemed inconsistent with that described in the legend.

*We thank the reviewer for pointing out this mistake. The legend has now been corrected in the revised manuscript it is Figure S3B.*

FigureS2C, perhaps modify the legend title from "Classification of overlapping regulatory region" to "Classification of overlapping regulatory region in current study" (check if this is correct) to increase clarity.

*That is correct, and has now been revised in Figure S3B.*

Figure S2D, it would be helpful to clarify in the caption about what each row represented for the bottom panel.

*We have provided clarification in the legend of the submitted Figure S2D, in the revised manuscript it is Figure S3C.*

Figure 5E appeared before Figure 5D in the main text.

*Though it is customary to cite panels in the text in alphabetical order, panel E appears before D in the text because we configured Figure 5 so as to avoid unnecessary whitespace in the design. It is not an ideal solution, but we would prefer to keep all the repeat analyses together in the current figure layout.*

**Second round of review**

**Reviewer 1**

Given the short length of single reads (50bp), it is surprising that the authors have identified that only 4% of their L1 reads were multimapped. Taking 15 consecutive 50bp sequences from a random L1PA5 sequence in the human genome and using BLAT (which considers all L1s) identified that only 5 out of 15 reads tested mapped uniquely. A concern that remains regarding the authors response to point 3 is that often the default parameters for alignment only command to search through some, instead of all L1 loci, to find a unique match and/or to randomly assign a read to a locus when a read maps to multiple loci. This means that some reads that may appear to align uniquely under the default parameters would fall into the multimapped category, if all L1 loci and/or custom alignment parameters were used. I understand the authors’ explanation that more divergent L1 sequences are likely to be contributing to the observed phenomenon, which would also increase their chances to map uniquely. The authors should check the default parameters that were used and add a description and limitations (if any) of these parameters to the materials and methods section.

**Reviewer 2**

All in all, I think both reviewers were very positive about the study and the manuscript even its original presentation. That said, a number of points were raised that would potentially further clarify and strengthen the presentation of the results.

Having read the response carefully I feel that the authors have now very carefully addressed all the points that were raised. I found the additional controls, figures, tables and text to have improved what was already a very noteworthy study. I have no further concerns and would like to congratulate the authors on a well-executed and interesting study.

**Authors Response**

**Point-by-point responses to the reviewers’ comments:**

Comment

*Response*
